# Biomechanical assessment of mandibular fracture fixation using finite element analysis validated by polymeric mandible mechanical testing

**DOI:** 10.1038/s41598-024-62011-4

**Published:** 2024-05-23

**Authors:** Omid Daqiq, Charlotte Christina Roossien, Frederik Wilhelm Wubs, Baucke van Minnen

**Affiliations:** 1 Department of Oral and Maxillofacial Surgery, University Medical Center Groningen, University of Groningen, Hanzeplein 1, 9713 GZ Groningen, The Netherlands; 2 Engineering and Technology Institute Groningen, Department of Bio-Inspired MEMS and Biomedical Devices, University of Groningen, Nijenborgh 4, 9747 AG Groningen, The Netherlands; 3 Bernoulli Institute for Mathematics, Computer Science and Artificial Intelligence, University of Groningen, Nijenborgh 9, 9747 AG Groningen, The Netherlands

**Keywords:** Translational research, Biomedical engineering, Fracture repair, Trauma

## Abstract

The clinical finite element analysis (FEA) application in maxillofacial surgery for mandibular fracture is limited due to the lack of a validated FEA model. Therefore, this study aims to develop a validated FEA model for mandibular fracture treatment, by assessing non-comminuted mandibular fracture fixation. FEA models were created for mandibles with single simple symphysis, parasymphysis, and angle fractures; fixated with 2.0 mm 4-hole titanium miniplates located at three different configurations with clinically known differences in stability, namely: superior border, inferior border, and two plate combinations. The FEA models were validated with series of Synbone polymeric mandible mechanical testing (PMMT) using a mechanical test bench with an identical test set-up. The first outcome was that the current understanding of stable simple mandibular fracture fixation was reproducible in both the FEA and PMMT. Optimal fracture stability was achieved with the two plate combination, followed by superior border, and then inferior border plating. Second, the FEA and the PMMT findings were consistent and comparable (a total displacement difference of 1.13 mm). In conclusion, the FEA and the PMMT outcomes were similar, and hence suitable for simple mandibular fracture treatment analyses. The FEA model can possibly be applied for non-routine complex mandibular fracture management.

## Introduction

In Oral & Maxillofacial surgery (OMF), mandibular fracture management is based on two essential principles: (1) Champy’s load sharing principle of placing semi-rigid miniplates corresponding to the ideal line of osteosynthesis to neutralise the tensile forces. The accompanying compression forces are compensated by the interfragmentary stability of the fracture. (2) The load bearing principle involves positioning a rigid solid plate at the lower border to avoid fragment displacement during functional movement of the mandible^1–4^.

In the last few decades after Champy’s principle was introduced, many studies have been conducted to evaluate mandibular fracture fixation, e.g., to develop new osteosynthesis materials, using time consuming and expensive polymeric or cadaveric model testing^5–7^. Nowadays, it might be favourable to replace physical mandible model assessments with computer aided three dimensional (3D) modelling and finite element analysis (FEA) tools for new developments^5–8^. FEA is a computational method to evaluate stress, strain, and displacement distribution within an assembly or structure applied in different fields (e.g., engineering or medicine)^9–11^. It is a non-invasive, flexible, valid, and precise instrument that can be used in OMF surgery for assessing the distribution of forces in different types of fractures or fixation methods (e.g., mandibular reconstruction and treatment in traumatology or oncological settings)^11–17^.

In recent years, there have been more FEA studies in the OMF area^14–23^ However, the application of FEA in clinical cases is limited in OMF surgery due to non-availability of a validated FEA model that can be routinely applied to mandibular fracture treatment. In principle, regarding complex fractures (e.g., comminuted or extremely resorbed mandibles), FEA can lead to a better understanding of fracture management (e.g., by determining and the visualisation of stress, strain, or displacements) and perhaps to new types of implants (e.g., patient tailored osteosynthesis). This means that there is a need for a validated FEA computation for mandibular fracture analysis. In a previous study, we investigated the Champy principle for unilateral simple mandibular body fracture management, using a simplified geometrical FEA model, as a proof of principle^24^. Our ultimate goal is to use a validated FEA model for analysis of more complex fractures, thereby avoiding the expensive and time consuming in vitro or in vivo tests.

The purpose of this study was to develop a validated FEA model for mandibular fracture management. This was achieved by assessing common mandibular fracture fixation with an innovative in silico FEA model, verified by a series of polymeric mandible mechanical testing (PMMT). We hypothesised that the mechanical behaviour of different types of simple mandibular fracture fixations correspond to the PMMT verified FEA model.

## Material and methods

### Study outline

The study applied the FEA simulation principle to analyse simple mandibular symphysis, parasymphysis, and angle fracture fixation with varying plate positions, using a 2.0 mm 4-hole miniplate system (1.0 mm miniplate thickness) with the maxDrive 2.0 × 6 mm screws (KLS Martin, Gebrüder Martin GmbH & Co., Tuttlingen, Germany). The FEA simulations were conducted in the Solidworks software computer simulation program (version SP5.0, 2021, 3D Modelling and Simulation, Waltham, Massachusetts, USA).

The FEA outcomes were validated by a series of polymeric mandible models fixated onto a custom-built apparatus in a mechanical test bench (Instron 3400, 34TM-5 dual column table model, Norwood, USA). The FEA simulations were conducted in an identical setting as the polymeric mandible mechanical testing (PMMT), namely: identical miniplate positioning, load application, fixation, and boundary conditions.

### 3D model

**Figure 1:**
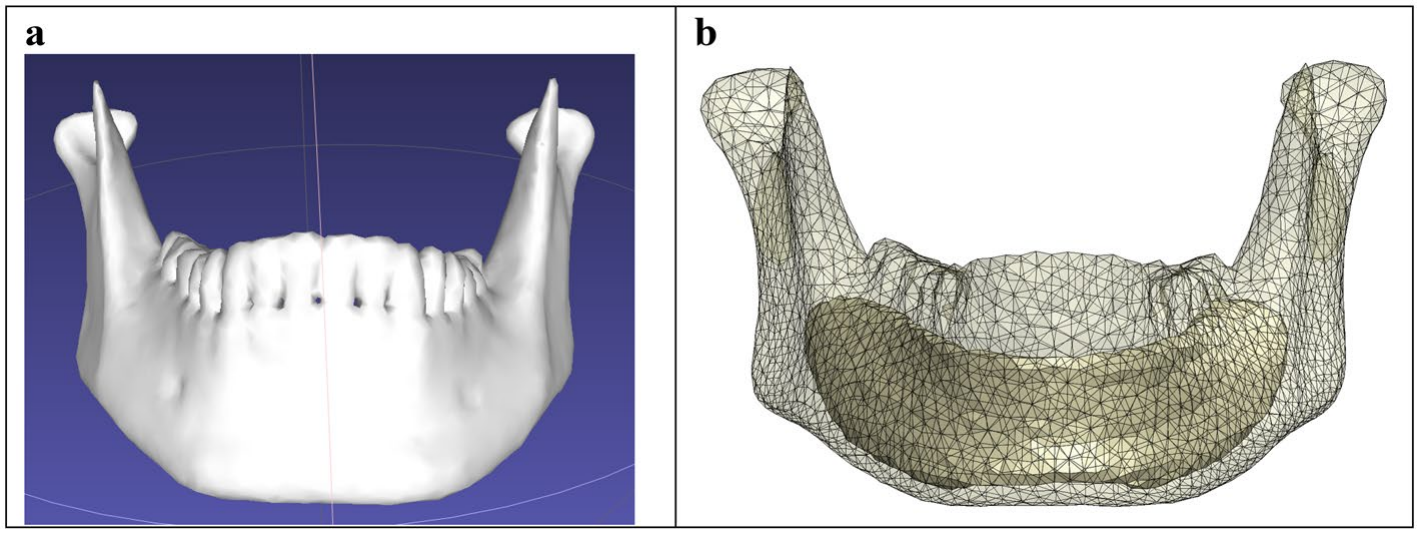
3D modelling of the mandible: (**a**) Synbone mandible segmentation in Mimics, (**b**) 3D mandible model containing the cortical and trabecular bones.

**Figure 2:**
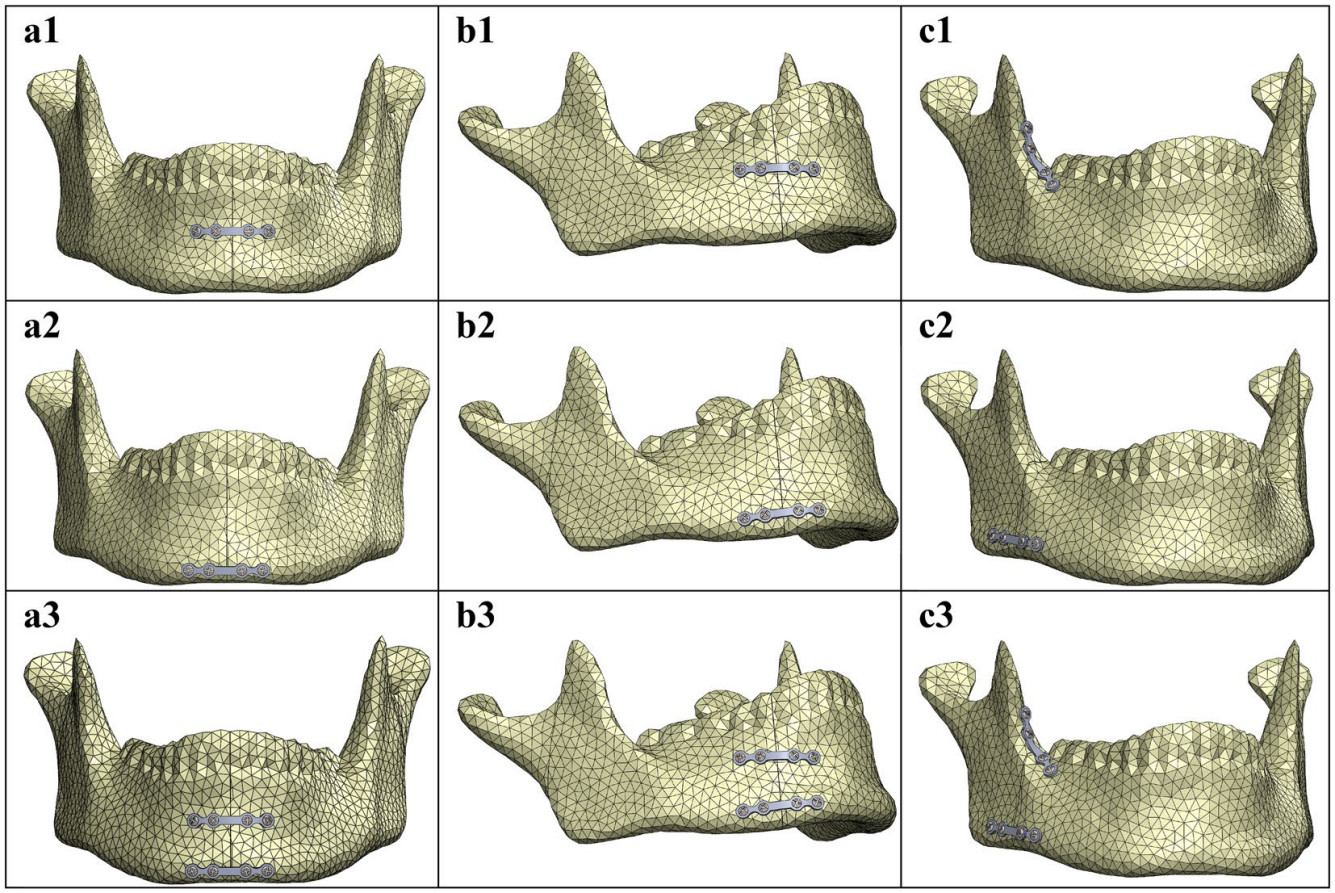
Plate positioning for mandibular (**a**) symphysis, (**b**) parasymphysis, and (**c**) angle fractures: (**1**) miniplate positioned at the superior border, (**2**) at the inferior border, and (**3**) the two plate combination.

Three mandibles with symphysis, parasymphysis, and angle fractures (Synbone, zizers, Switzerland) were selected to create 3D models of the mandible. Digital imaging and communication in medicine (DICOM) files of the mandibles were obtained from cone beam computed tomography (CBCT) scans (Planmeca Promax, 3D-Max ProFace, Helsinki, Finland). The CBCT scans were performed according to bone setting with 400 µm voxel size, 120 kV tube voltage, and 2.5 mA tube current. Mandible segmentation was performed by using the Mimics software (version 20.0, Materialise, Leuven, Belgium) (Fig. 1a). The trabecular bone volume segmentation was assigned by using a new mask, changing the Hounsfield unit (HU) threshold, and using multiple slice editors in Mimics. The segmented mandible was wrapped and smoothened with the 3-Matic software (version 15, Materialise, Leuven, Belgium). The cortical and trabecular bone sections were then combined into a single mandible assembly file using Geomagic (Solidworks 2021 add-in, 3D systems, Rock Hill, South Carolina, USA). Geomagic was used to solve the segmentation geometrical mesh errors and to create a workable organic mandible assembly file that could be flawlessly imported and used in the Solidworks software. The 3D mandible model (containing the cortical and trabecular bone) was exported to the Solidworks computer simulation software in a stereolithography (STL) file format (Fig. 1b). The study used Solidworks software for 3D modelling and FEA simulation analysis. The 3D mandibles contained a single simple fracture at the symphysis, parasymphysis, and angle region, identical to the Synbone polyurethane models, with a fracture surface distance of 0.1 mm (Fig. 2). The 0.1 mm distance was based on the measured fracture surface distances from the fixated mandible replicas used in the PMMT.

A 2.0 mm 4-hole osteosynthesis titanium miniplate (KLS Martin: nr. 25-551-04-09; Dimensions: 26 mm length, 4.3 mm width, and 1 mm thickness) and a maxDrive 2.0 × 6 mm screw (KLS Martin, nr. 25-872-05-09; Screw dimensions: 2 mm diameter and 6 mm length) were modelled in Solidworks. The miniplate was used for all the fracture fixations (Fig. 2).

### Polymeric mandible mechanical testing (PMMT)

Various Synbone mandible (symphysis, parasymphysis, and angle) fractures were fixated with KLS Martin 2.0 mm 4-hole miniplates by an experienced OMF surgeon, using the LevelOne 2.0 mm KLS Martin surgical mini instrument system (2.0 mm Mini Module Regular Trauma set, KLS Martin, Gebrüder Martin GmbH & Co., Tuttlingen, Germany) (Supplementary Fig. S1). The miniplates were bent according to the shape of the mandible. The superior miniplates were placed at the ideal line of osteosynthesis, and the inferior miniplates at the lower border. Positioning the miniplate on the mandible (e.g., bending, drilling, and screwing) was performed with the manufacturer’s original instruments (KLS Martin, GmbH & Co., Tuttlingen, Germany).

**Figure 3:**
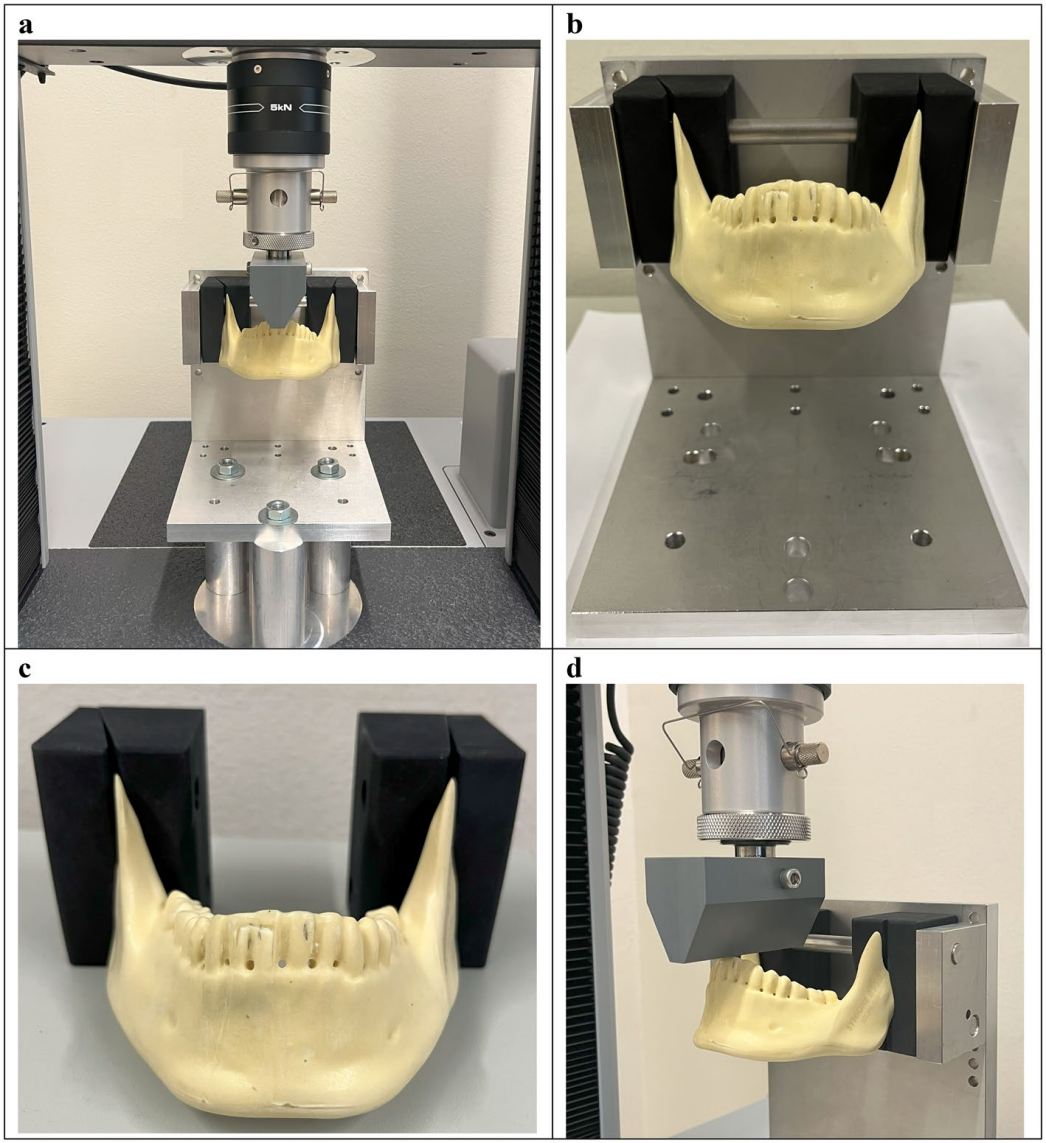
Polymeric mandible mechanical testing (PMMT). (**a**) The test set-up for positioning the mandible inside the mechanical test bench. (**b**) Custom fabricated apparatus for placing the mandible onto the mechanical test bench. (**c**) Custom 3D printed mandible holders for fixating the mandible inside the custom fabricated apparatus. (**d**) Load applied on the anterior of the mandible via a custom 3D printed part mounted on the mechanical test bench load cell.

An Instron mechanical test bench was used for conducting the PMMT (Instron 3400, 34TM-5 dual column table model, Norwood, USA) (Fig. 3a). The mechanical test bench was calibrated in advance, with a precision and accuracy of less than 1%. The PMMT was performed three times for each of the mandible replicas and each of the plate configurations whereupon a total of twenty-seven models were tested. This eliminated the risk of measurement bias. An automated test protocol was created inside the Instron’s software (BlueHill Universal) to get an identical test set-up for all the mandible replicas.

An apparatus was custom fabricated so that the mandible could be placed onto the mechanical test bench (Fig. 3b), made of AISI (the American Iron and Steel Institute) 316 stainless steel (screws and two rod bars) and aluminium alloy (rest of the apparatus). Each mandible was secured inside the custom apparatus by 3D printed mandible holders made of Nylon (polyamide type 12), from the condyle to mid-ramus, just before the mandibular foramen (Fig. 3c). The two holes in each mandible holder were the same size as the stainless steel rod bars of the custom made apparatus which held the mandible in a fixed position. This eliminated movement, rotation, or translation of the mandible during mechanical testing. Furthermore, since Nylon can deform during multiple loading, the mandible holders were replaced after 9 tests, resulting in a total of 3 mandible holders being used during the PMMT.

The force on the mandible was applied by a custom 3D printed part, which was mounted on the mechanical test bench load cell (capable of a maximum of 5 kilonewtons [kN]) (Fig. 3d). The custom 3D printed part was made of Nylon (polyamide type 12) reinforced with an AISI 316 stainless steel ring in the centre hole that was mounted on the mechanical test bench load cell.

The mandibles were placed at an identical position inside the mechanical bench for each test. The load cell was automatically positioned at a predefined zero position. When the testing started, the mandible was set at a preload of 5 Newtons [N]. Then the load started to increase continuously at a rate of 1 Newton per second [N/s] until the failure point where the mandible’s breaking point was reached. The mechanical test bench could only measure the displacement as an outcome. The displacement was recorded according to the mechanical test bench’s load cell movement from the zero-position until reaching the failure point (Appendix 1: PMMT outcomes at the failure point).

### Finite element analysis (FEA)

#### Assembly modelling

The FEA analysis started with positioning the 2.0 mm osteosynthesis miniplate at the fracture site. The miniplates were positioned at three configurations, namely: one superior plate, one inferior plate, and a two plate combination method with one miniplate located superiorly and the other inferiorly (Fig. 2).

#### Force and fixation

**Figure 4:**
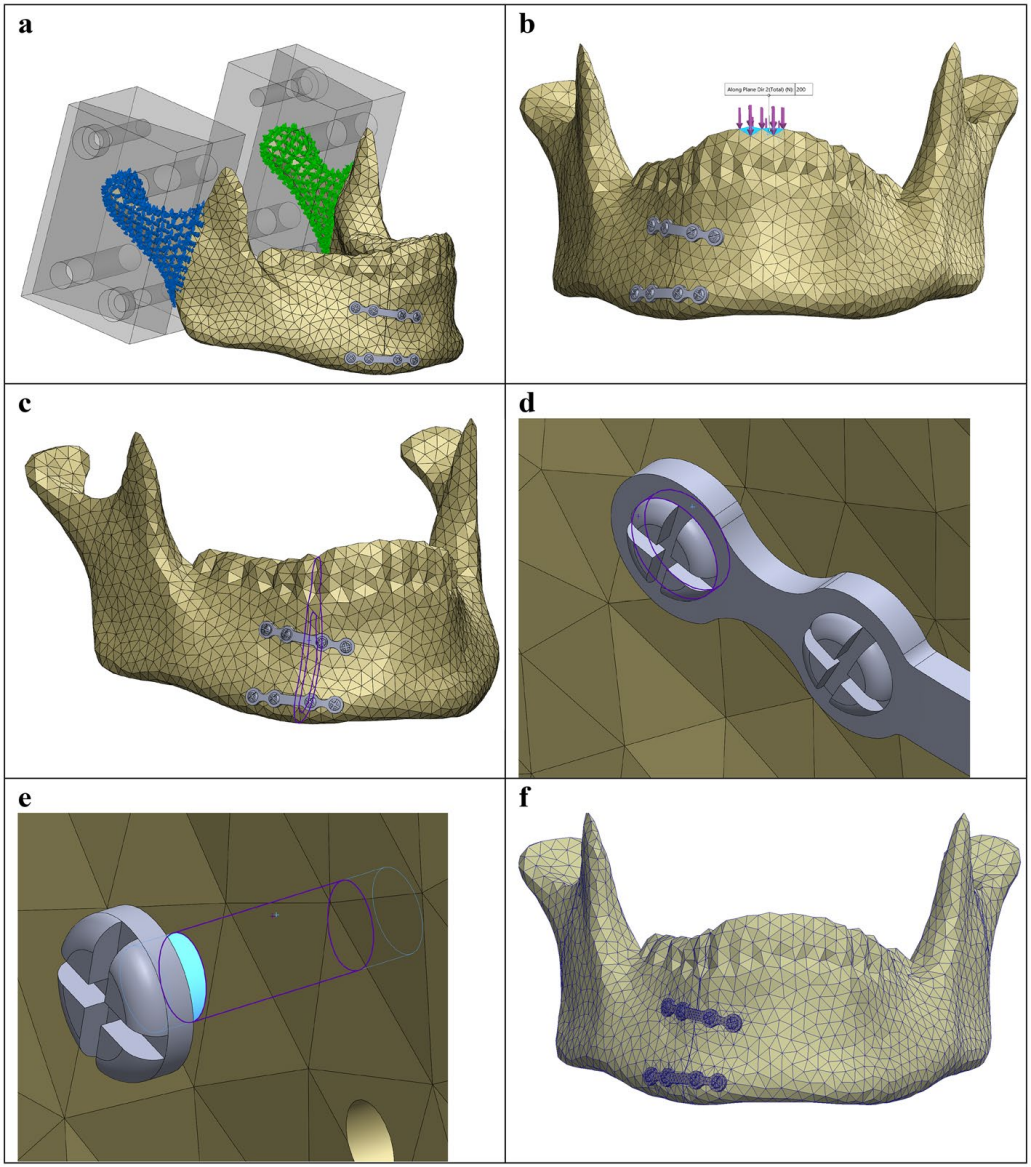
FEA set up in Solidworks. (**a**) Mandible fixation using fixed geometry (from the condyle to mid-ramus, just before the mandibular foramen), identical to fixation with the mandible holder in the PMMT. (**b**) Mastication force of 200 N applied downwards on the anterior of the mandible, identical to the load application in the PMMT. (**c**) Contact-Set boundaries between the fracture surfaces with a fracture distance of 0.1 mm and no penetration. (**d**) Contact boundary condition between the miniplate and screws. (**e**) Contact-Set boundary condition between the mandible and osteosynthesis. (**f**) Impression of the used converged mesh.

The mandible fixation and load application in the FEA was identical to the PMMT set-up (Fig. 3). The FEA fixation configuration was matched to the mandible holder’s fixation clamp system used in the PMMT (Fig. 4a). This was achieved by first placing the mandible holders on the mandible at the identical position as in the PMMT (from the condyle to mid-ramus, just before the mandibular foramen). Afterwards, the mandible fixation nodes were selected based on the mandible holder reference lines. The line indicates the border where the mandible is not being held anymore by the mandible holder. Furthermore, the fixation was defined by using the fixed geometry option in Solidworks.

An average mastication force of 200 N was applied downwards on the mandible at the two incisor teeth, corresponding to the exact same location as the force applied in the PMMT (Fig. 4b). The 200 N force was chosen for two reasons, namely: (1) it is an average mastication force based on the literature^25–28^; and (2) this force is the best for comparing the FEA outcomes with those of the PMMT since, beyond this force, some of the mandible samples started to break during the mechanical testing (Appendix 1).

#### Synbone mandible material properties

The study used Synbone polymeric mandibles for both the mechanical testing and creating a 3D mandible model. Synbone is made of polyurethane (PU) synthetic foam with varying density, mimicking cortical and trabecular bone^29–31^. A few studies in the literature have evaluated the material properties of Synbone and all these studies show differences in mechanical properties^29–33^. Therefore, to increase the accuracy of this study, it was decided to determine the mechanical material properties of the Synbone mandible. This was achieved by a series of mechanical tests of Synbone foam blocks, namely: Generic Block (GB) and Generic Block High Density (GBHD). The decision to use these foam blocks was taken because, according to the manufacturer, their PU density is similar to that of the cortical and trabecular bone sections of Synbone mandibles. Test strip samples were made from the foam blocks for mechanical testing according to the American Society for Testing and Materials (ASTM) dimensions (Appendix 2, Fig. A1). A calibrated Zwich/Roell mechanical test bench (TC-FR2, 5TS.D09, Z2.5 kN model; positioning accuracy 0.0001 mm, force accuracy 0.2%; Zwick/Roell, Venlo, the Netherlands) was used for the mechanical testing. The test strip samples were clamped onto the mechanical test bench using 3D printed grips made of Nylon (polyamide type 12) that were attached to the machine using screws (Appendix 2, Fig. A1b). The speed during the testing was set at 10 mm/min. The size of the test strips was measured before, during (at intervals of 20 N), and after testing. Multiple strips from each block were tested to get an accurate outcome measure and to eliminate measurement errors (Appendix 2, Fig. A1c,d). The mechanical properties were calculated for each test strip. Finally, all the data were analysed by a statistical expert with analysis of variance (Appendix 2, Table A1). The observed standard error was 3.47 for the cortical bone and 1.12 for the trabecular bone.

Finally, the material properties of the mandible were set according to the Synbone’s material property testing outcomes. The cortical mandible’s material properties were set at an elastic modulus of 196.86 megapascals [MPa], tensile strength of 6.68 MPa, yield strength of 48.12 MPa, mass density of 0.35 g/cm3, and Poisson’s ratio of 0.10. The trabecular mandible’s material properties were set at an elastic modulus of 60.98 MPa, tensile strength of 3.31 MPa, yield strength of 23.80 MPa, mass density of 0.19 g/cm3, and Poisson’s ratio of 0.09.

#### Osteosynthesis material properties

The titanium osteosynthesis’ (miniplate and screws) properties were: elastic modulus of 104,800 MPa, tensile strength of 1100 MPa, yield strength of 827.40 MPa, mass density of 4.43 g/cm3, and Poisson’s ratio of 0.31^34^.

#### Boundary conditions

The Solidworks contact-sets property manager tab enabled defining the boundary conditions and interactions between the mandible, miniplate, and screws (Fig. 4c–e). The interactions between the components were in accordance with the current opinion of mandibular fracture stabilisation based on a non-locking compression plate^25–28^. The cortical and trabecular bone segments were set as bounded with a 0 mm gap range using the component interaction tab in Solidworks. This meant the two fixed mandible bone segments behaved as one segment but, at the same time, kept their own identical mechanical behaviours during the simulation.

The rest of the component interactions were set by using the local interaction tab in Solidworks. The mandible fracture surfaces were defined by using contact-sets with a 0.1 mm fixed distance between the surfaces (Fig. 4c), representing optimal fracture reduction, and was in line with polymeric model testing. When fracture surfaces touch under loading, there was no friction and only forces normal to the surfaces could be exchanged. The connection between the miniplate and the screws were set as a contact in the contact-set properties (Fig. 4d). There was no friction between these components and only normal forces were exchanged between the components. The interaction between the screws and the mandible screw holes were defined as bounded (Fig. 4e). This means that the screw was tightened inside the screw hole in the mandible, keeping the miniplate fixed against the mandible surface. Finally, the connection between the mandible and the miniplate was set as the contact. This means that the miniplate was in contact with the surface of the mandible and was held firmly by the miniplate screws (the so called non-locking compression plating method).

#### FEA mesh convergence

The mesh size was investigated prior to running the FEA simulation analysis. Convergence of the solution was reached by reducing the mesh until the peak Von-Mises stress, in megapascals [MPa], became independent of the mesh size (Supplementary Fig. S2: mesh convergence plot). This led to a controlled mesh with a minimum element size of 1.68 mm and a maximum element size of 5 mm, which was used for the FEA studies (Fig. 4f).

## Data analysis

**Figure 5:**
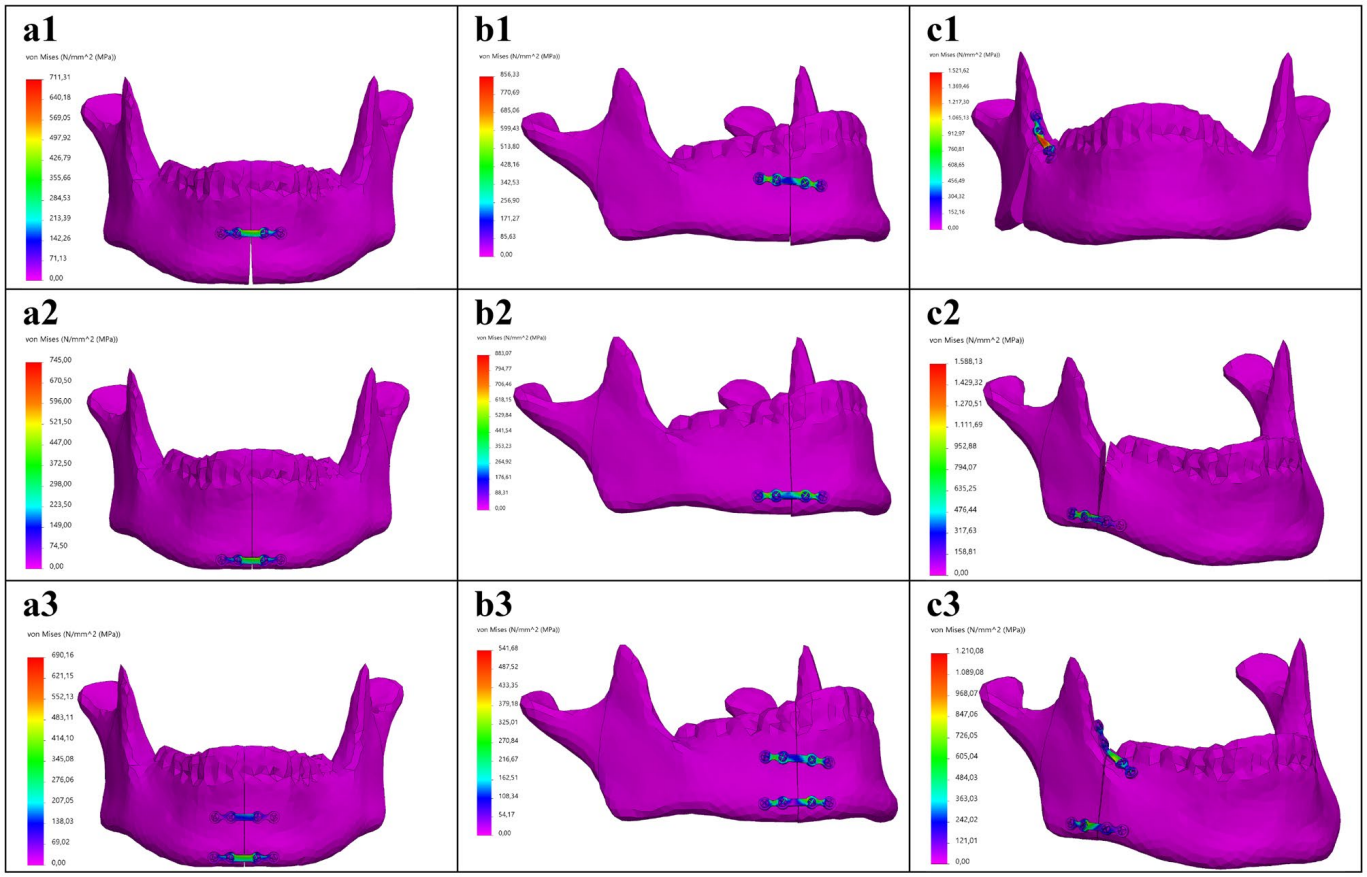
FEA Von-Mises stress pattern at 200 N load for (**a**) symphysis, (**b**) parasymphysis, and (**c**) angle fractures; with the miniplate positioned respectively at (**1**) the superior border, (**2**) inferior border, and (**3**) the two plate combination.

**Figure 6:**
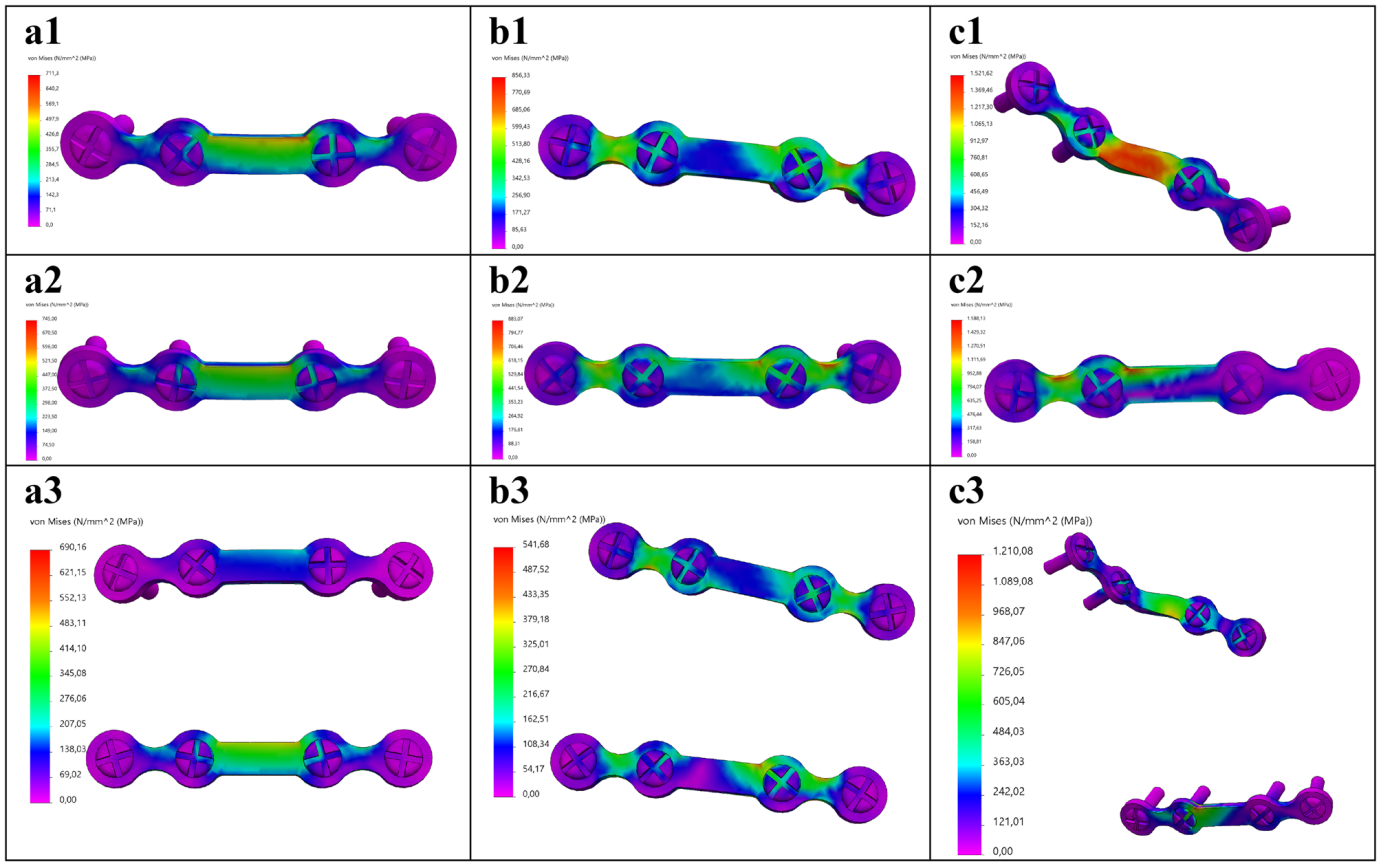
Frontal view of the FEA Von-Mises stress pattern on the osteosynthesis at 200 N load for: (**a**) symphysis, (**b**) parasymphysis, and (**c**) angle fractures; with the miniplate positioned respectively at (**1**) the superior border, (**2**) inferior border, and (**3**) the two plate combination.

**Figure 7:**
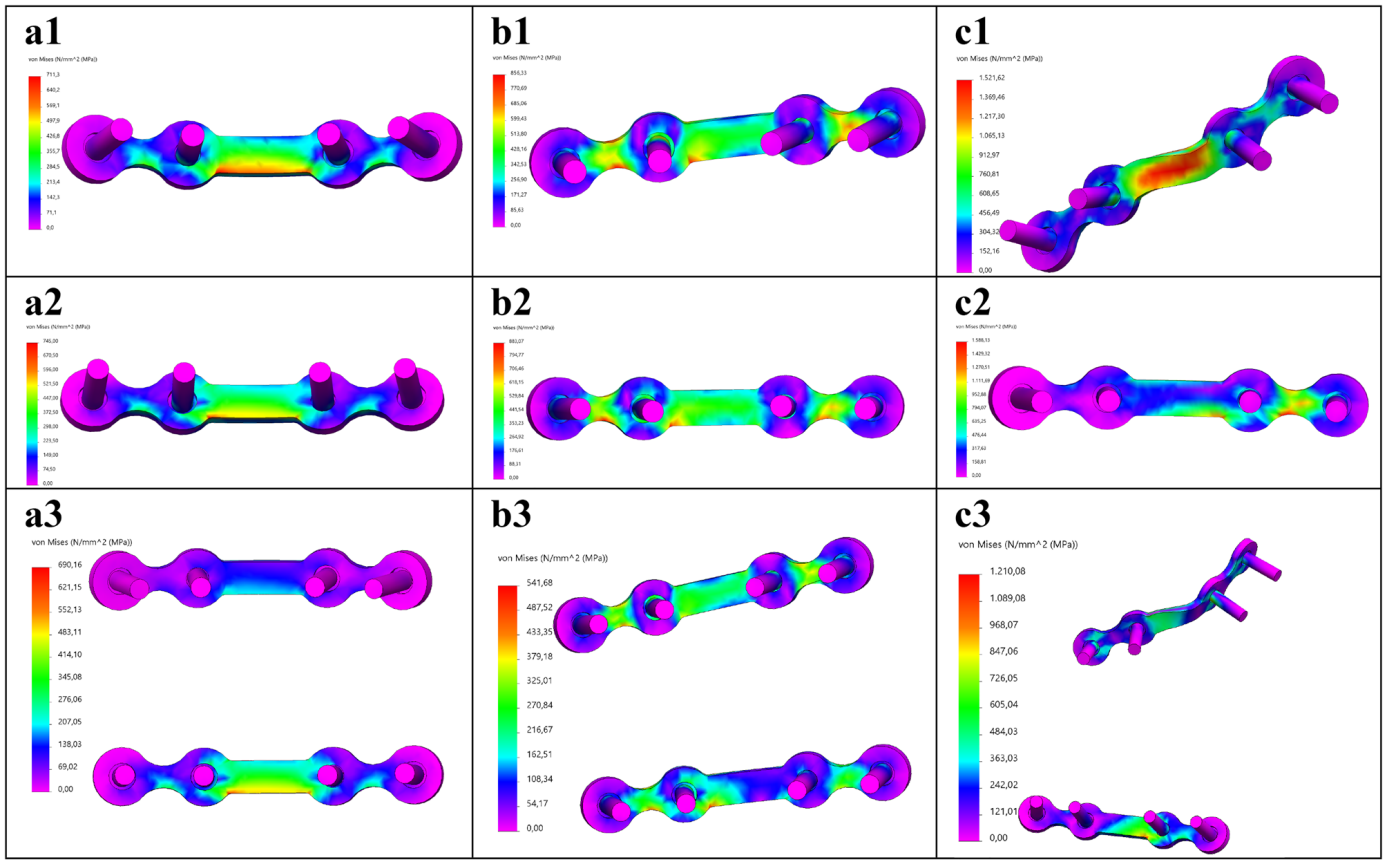
Back view of the FEA Von-Mises stress pattern on the osteosynthesis at 200 N load for: (**a**) symphysis, (**b**) parasymphysis, and (**c**) angle fractures; with the miniplate positioned respectively at (**1**) the superior border, (**2**) inferior border, and (**3**) the two plate combination.

The FEA outcomes were evaluated by firstly investigating the stress distribution, the maximum Von-Mises stress and its location (Table 1, Figs. 5, 6, 7, Fig. 9a). Secondly, the displacement was investigated in the FEA (Table 2). Furthermore, the outcomes of the mechanical testing were evaluated in terms of displacement in the PMMT (Table 2). Finally, the FEA outcomes were compared with the PMMT’s displacement patterns for the various fracture types and plate configurations under a 200 N force (Table 2, Fig. 9c,d). The FEA’s displacement in the Z-axis (same axis as the force applied in the mechanical testing) was used for comparison purposes with the PMMT’s displacement.

**Table 1:** The FEA maximum Von-Mises stress and displacement outcomes.

Mandibular fracture	Miniplate configuration	Von-Mises stress [MPa]
Symphysis	Superior	711.31
	Inferior	745.00
	Two plate	690.16
Parasymphysis	Superior	856.33
	Inferior	883.07
	Two plate	541.68
Angle	Superior	1521.62
	Inferior	1588.13
	Two plate	1210.08

**Table 2:** Polymeric mandible mechanical testing (PMMT) displacement compared to FEA displacement at 200 N force.

Mandibular fracture	Miniplate configuration	Test number PMMT*	Displacement [mm] at 200 N		Displacement Difference ***
			PMMT*	FEA**	
Symphysis	Superior	1	3.82		
		2	5.33		
		3	5.18		
		Mean ± SD [95% CI]	4.78 ± 0.83 [0.94]	5.82	1.05
	Inferior	1	5.06		
		2	3.99		
		3	4.44		
		Mean ± SD [95% CI]	4.50 ± 0.54 [0.61]	5.68	1.19
	Two plate	1	4.80		
		2	4.66		
		3	3.87		
		Mean ± SD [95% CI]	4.44 ± 0.50 [0.57]	5.55	1.11
Parasymphysis	Superior	1	4.92		
		2	4.93		
		3	4.64		
		Mean ± SD [95% CI]	4.83 ± 0.16 [0.18]	6.03	1.20
	Inferior	1	4.27		
		2	6.06		
		3	4.56		
		Mean ± SD [95% CI]	4.96 ± 0.96 [1.09]	6.06	1.10
	Two plate	1	3.96		
		2	4.55		
		3	4.54		
		Mean ± SD [95% CI]	4.35 ± 0.34 [0.39]	5.51	1.16
Angle	Superior	1	5.16		
		2	4.78		
		3	6.33		
		Mean ± SD [95% CI]	5.42 ± 0.81 [0.92]	7.25	1.83
	Inferior	1	7.08		
		2	6.50		
		3	7.18		
		Mean ± SD [95% CI]	6.92 ± 0.37 [0.42]	8.14	1.22
	Two plate	1	5.39		
		2	5.33		
		3	4.83		
		Mean ± SD [95% CI]	5.18 ± 0.31 [0.35]	5.48	0.30

*For the PMMT: each plate configuration was repeated three times for each fracture under exact conditions (Test numbers 1–3). Furthermore, the mean, standard deviation (SD), and 95% confidence interval (CI) of the three repeated tests are shown.

**The FEA displacements are the exact values from the computer simulation at 200 N force.

***Displacement difference between the FEA and PMMT in millimetres.

In terms of statistics, the FEA and the PMMT outcomes were analysed by statistical experts, using SPSS (version 26, IBM corporation, Armonk, New York). First, analysis of variance was applied to the Synbone material properties’ testing outcomes. This determined the mean, standard deviation, and standard error of each material property parameter (Appendix 2, Table A1). Secondly, descriptive statistics was applied to determine the mean displacement, standard deviation, and 95% confidence interval of the repeated PMMT (Table 2). Furthermore, the displacement difference between the FEA and the PMMT testing was calculated (Table 2, Fig. 9c,d). Finally, other statistics was not applicable, because the sample size was not big enough for a sensible statistical analysis.

## Results

### Finite element analysis (FEA)

The FEA outcomes illustrate the maximum Von-Mises stress in megapascals [MPa] (Table 1) and displacement in millimetres [mm] (Table 2). The stress distribution and the maximum stress varied according to the different fracture types and plate configurations (Figs. 5 and 9a). In all cases, the maximum stress was located on the osteosynthesis miniplate (see Figs. 6 and 7 for detailed frontal and back views of stress distribution on the miniplate). The Von-Mises stress was lower in the two plate combination for all the fracture types (symphysis, parasymphysis, and angle) compared to the single superior or inferior plate positioning (Figs. 5a3–c3, 9a). Compared to the single superior border (Fig. 5a1–c1), the single inferior border (Fig. 5a2–c2) plate positioning outcomes were not so satisfactory as the Von-Mises stress had increased, the fracture surfaces tended to open, and the fixation became unstable during loading. This effect was more visible for the angle fracture, where the amount of stress had increased dramatically compared to the symphysis and parasymphysis fractures.

**Figure 8:**
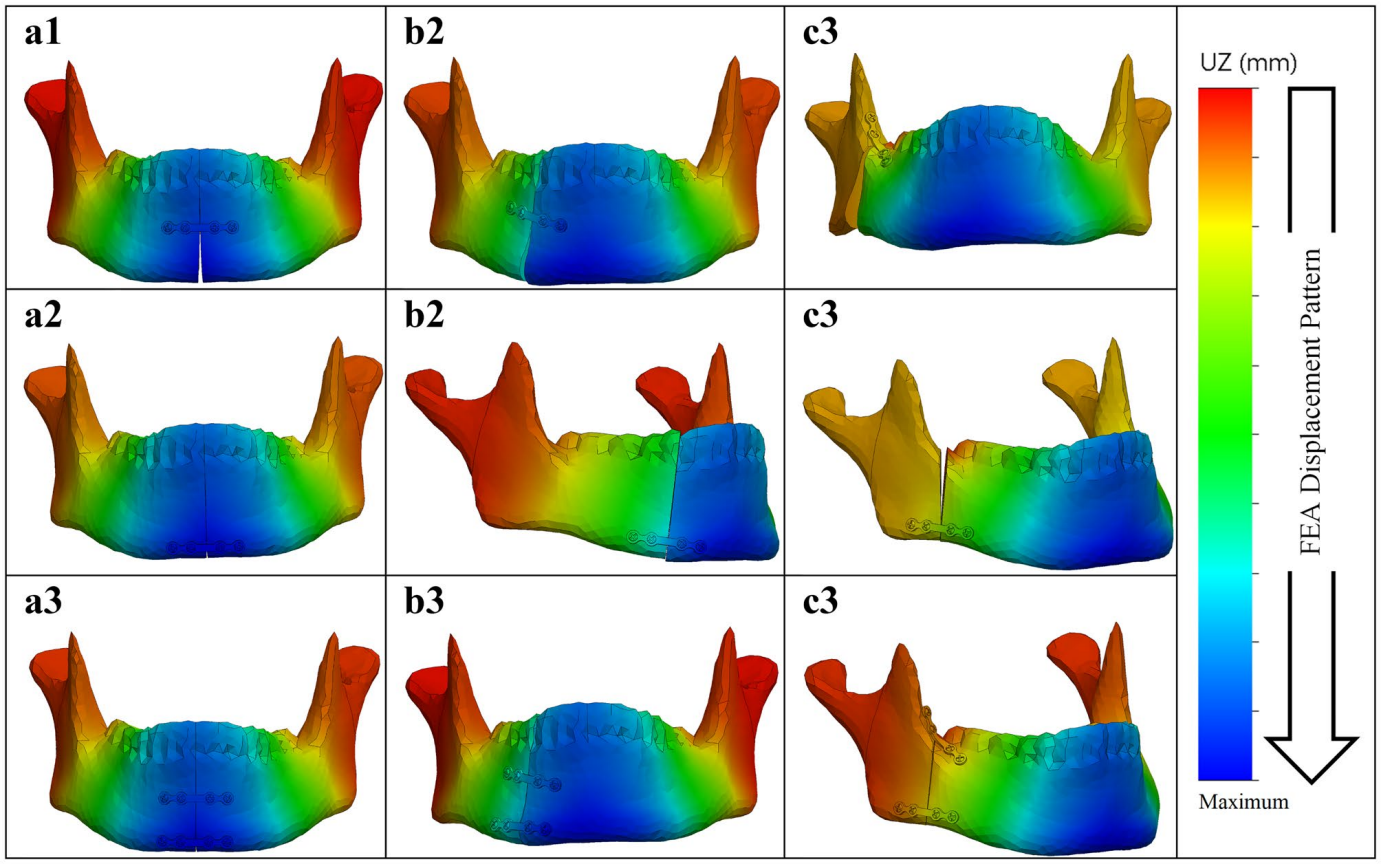
FEA displacement pattern with a 200 N load for (**a**) symphysis. (**b**) parasymphysis, and (**c**) angle fractures; with the miniplate positioned respectively at (**1**) the superior border, (**2**) inferior border, and (**3**) the two plate combination. *Note* The colour legend on the right side illustrates the assembly displacement pattern (with the blue colour representing the maximum displaced region).

**Figure 9:**
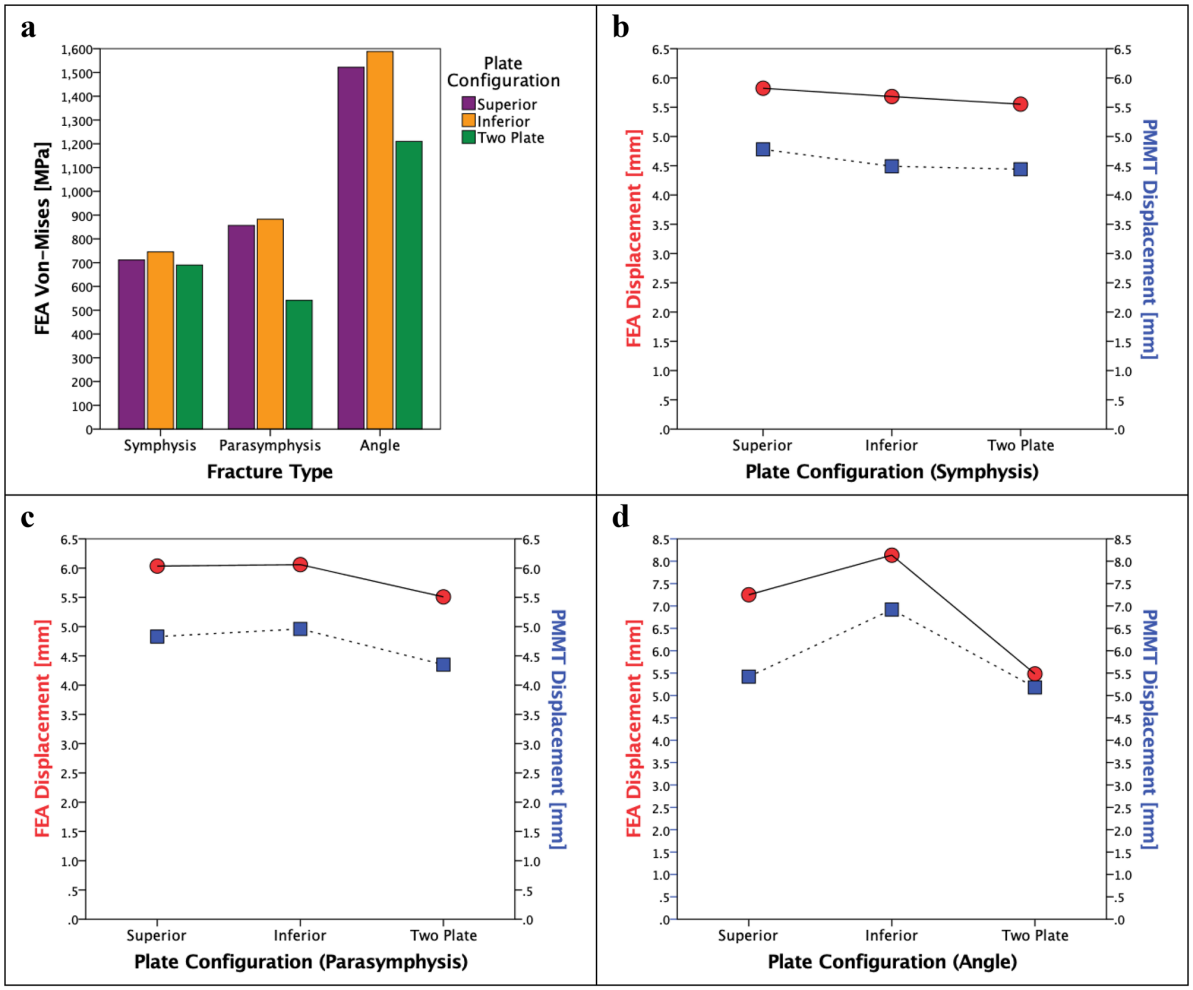
(**a**) FEA Von-Mises stress in [MPa]. (**b**–**d**) FEA (red) versus PMMT (blue) displacement for the three different plate configurations in [mm]: (**b**) symphysis, (**c**) parasymphysis, and (**d**) angle fractures.

In terms of displacement, the outcome patterns were similar to the stress outcomes (Table 2, Figs. 8, 9b–d). The displacement of the two plate combination was marginally lower compared to the single superior and the single inferior plate positioning in all the fracture types (Fig. 9b–d). Regarding the symphysis fracture, the superior border plate demonstrated slightly higher displacement compared to the inferior border plate (difference of 0.14 mm). Regarding the parasymphysis fracture, the inferior border plate exhibited slightly higher displacement than the superior plate (difference of 0.03 mm). Regarding the angle fracture, the difference became more obvious, where the superior border plate’s displacement was much lower than that of the inferior border plate (difference of 0.89 mm).

### Polymeric mandible mechanical testing (PMMT)

**Figure 10:**
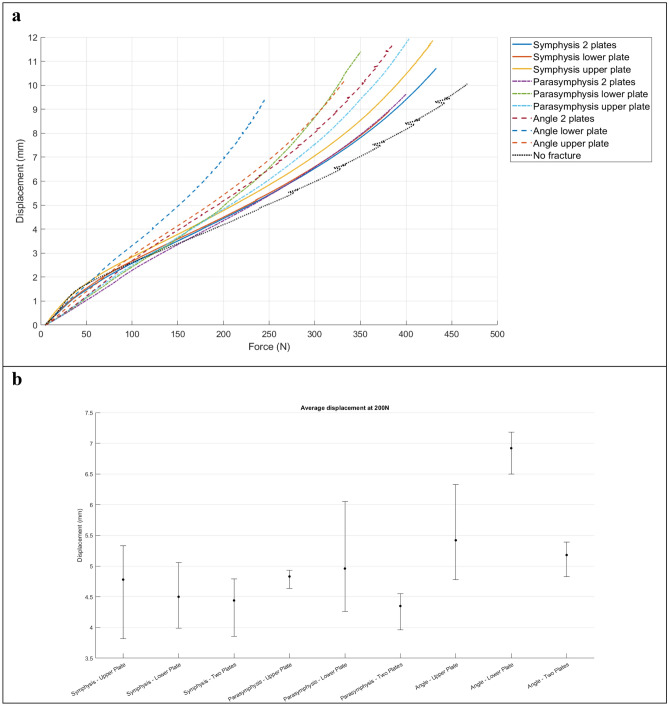
(**a**) Average force (x-axis) versus displacement (y-axis) of the PMMT for each mandible configuration. (**b**) Mean displacement and range for the 3 repeated tests at 200 N force. Upper plate (superior border), Lower plate (inferior border), Two plates (superior and inferior border plate combination), and Average (mean).

The PMMT outcomes are shown in Table 2 in terms of displacement [mm] on applying a 200 N force compared to the FEA displacement. The mechanical testing was performed until failure point was reached, where the polymeric mandible broke when applying the load (Fig. 10: PMMT load–displacement graph). At the failure point, all the mandible’s broke at the region where the mandible was fixated with the 3D printed Nylon mandible holders (Appendix 1, Figs. A1–A3: the PMMT break pattern at the failure point). In all cases, the mandible broke at a much higher force than 200 N, with some cases at just above a 200 N load (Appendix 1, Table A1: the PMMT failure force [N] and the maximum displacement [mm]). Therefore, displacement at a force of 200 N was chosen for comparison purposes between the mechanical testing versus FEA in the Z-axis (same direction as the force applied during the mechanical testing).

In the PMMT, the two plate combination displayed the lowest displacement of all the fractures (Fig. 9b–d). Regarding the symphysis fractures, the superior border plate position demonstrated slightly higher displacement compared with the inferior border plate position (difference of 0.29 mm). Displacement was slightly lower for the parasymphysis fractures when the plate was in a superior border plate position compared to an inferior border position (difference of 0.13 mm). Regarding angle fractures, the displacement was much smaller for the superior border plate position than the inferior border plate position (a difference of 1.50 mm).

### FEA versus PMMT

The displacement outcome differences between the FEA and the PMMT are shown in Table 2. In all the cases, the FEA displacement was higher compared to the PMMT displacement (a total mean difference of 1.13 mm); however, the displacement patterns of both testing methods were similar for the different fracture types and plate configurations (Fig. 9b–d).

## Discussion

This study strived to create a validated 3D modelling and FEA numerical simulation principle for mandibular fracture management. In the study, we observed four major outcomes. First, the FEA outcomes are in line with the current understating of mandibular fracture fixation. Earlier literature suggested that the use of a single superior border miniplate may result in the least morbidity based on fracture distraction tension lines^2,35^. However, this may become a problem during functional loading due to the anatomy and biomechanics of the mandible, whereas a second inferior border miniplate would be necessary to protect the fracture site against the bending and torsional movement forces^36–43^. Such an effect was observed in our study, where the two miniplate combination generated more stability compared to a single miniplate (Table 1, Fig. 9). In the FEA, the stress on the miniplate resulted from the mandible deformities caused by the fracture. This means the miniplate(s) must hold the fracture surfaces in a stable anatomical position when a load is applied, and therefore it must withstand a huge amount of stress (Figs. 5). In the two plate method, the superior border miniplate neutralised the tension forces whereas the inferior border miniplate stabilised the compression forces (Figs. 5, 6, 7, 8). Conversely, placing a single inferior border plate generated the least fracture stability during functional loading (Figs. 5, 6, 7, 8).

The FEA stress outcomes (Fig. 5) suggest that all the deformations occurred on the miniplate(s), since we observed that the stress distribution in the mandible was almost constant. This may insinuate that the whole or part of the mandible can be treated as a rigid body. However, the former can be excluded by the fact that a completely rigid mandible would lead to zero stresses in the plates. To get a first impression of where the mandible can be treated as a rigid body, we applied a 200 N load to a non-fractured mandible (Supplementary Fig. S3). The first observation was that the maximum peak Von-Mises stress of the non-fractured mandible was about 101.14 MPa (Supplementary Fig. S3a), which in Fig. 5 is in the lower range of the colour bar and hence hardly visible in those plots. However, carful observation of Fig. S3a1 illustrates that the stress in the red range of the colour bar can be traced in the exact spot in the blue range of the colour bar in Fig. 5. Secondly, the maximum stresses occurred at the fixation border with the mandible holders (Supplementary Fig. S3a1), indicating that this is the most vulnerable spot of the mandible in the study set-up. Using section clipping allows one to create a contour of the mandible regions, with the Von-Mises being equal or greater than 10 MPa (Supplementary Fig. S3a2). This shows that the stresses were smaller than in the remainder of the mandible which suggests that, apart from the fixation border area (in the mandibular ramus near the foreman mandibula) and possibly the area around the miniplate(s) regions, one could model the mandible with rigid bodies^44–47^. Therefore, we will investigate the possibilities in future studies regarding reducing the complexity of the FEA model.

Second, the outcomes of the FEA and the PMMT were consistent and comparable. The PMMT was used to verify the FEA outcomes. The study set-ups were identical in both the FEA and PMMT (e.g., identical miniplate positioning, load application, fixation location, and boundary conditions). Furthermore, the mechanical test bench in the study could only calculate the displacement as the output outcome. Therefore, only the FEA displacement in the Z-axis (same direction as the applied force in the mechanical testing) could be compared to the displacement outcome of the PMMT. However, both studies’ outcomes showed a similar displacement pattern (Table 2, Fig. 9b–d). In all the fracture scenarios, displacement of the two miniplate combination was lower compared to the single superior or inferior border plate (Fig. 9b–d). Furthermore, there was a small consistent displacement difference between the FEA (slightly higher) versus the PMMT (Table 2). In the symphysis and parasymphysis fracture cases, there was an average displacement difference of 1.10 mm between the FEA versus the PMMT. In the angle fracture cases, the displacement varied marginally more between the FEA versus the PMMT; namely: 0.30 mm for the two miniplate combination, 1.83 mm for the superior border plate, and 1.22 mm for the inferior border plate.

The displacement difference between the FEA and the PMMT seems to be due to structural differences caused possibly by several factors. The first is the environmental differences between the FEA and the PMMT. FEA applies a numerical simulation to calculate the amount of stress or displacements (Figs. 5, 6, 7, 8). Displacement in the PMMT, on the other hand, is measured according to the movement of the mechanical test bench load bar from the pre-determined zero position to the end position where the failure point is reached (Fig. 10; Appendix 1). Secondly, fracture reduction may influence the outcomes. In the FEA studies, a gap of 0.1 mm was used based on the measured fracture surfaces distance from the fixated mandible replicas used in the PMMT. Regarding the PMMT, mandible fracture reduction and fixation with a miniplate was done by an expert surgeon; however, in the Synbone models, the fracture resembled a saw cut instead of a true fracture. This may complicate the comparison with a true fracture reduction in the models. In addition, during the PMMT, the mandible was fixated using custom 3D printed mandible holders and placed in the custom apparatus for correct exact placement of the mandible onto the mechanical test bench (Fig. 3). This process might cause a minor alteration in the fracture surfaces distance in the different mandible replicas. We recommend that future studies should, if possible, measure the fracture gap after placing the mandible onto the mechanical test bench and use the corresponding distance in the FEA model. Thirdly, the composition of compact and trabecular bone may influence the outcomes. This is illustrated by the 95% confidence interval of the three repeated tests from each scenario (Table 2), illustrating a wide range in the PMMT sample data. Finally, we considered applying statistics for displacement comparison purposes between the FEA and the PMMT; however, it was not applicable due to the small sample size. Perhaps future studies could use a larger sample size.

Third, many studies in the literature have tested the Synbone mandible replicas mechanically to validate their FEA model^27,48,49^ ; however, Synbone mandible replicas might not be a suitable material for FEA model validation through mechanical testing. First of all, Synbone does not provide information regarding the exact material properties of their mandible replicas of the cortical and trabecular bone sections. Secondly, investigation of the Synbone material properties in the literature illustrates different values in the studies^27,48,49^, causing confusion regarding the true biomechanical material property values of Synbone mandible replicas^27,48,49^. Thirdly, this study’s investigation of the material properties of Synbone foam blocks with densities close to the cortical and trabecular bone segments (provided by the manufacturer) illustrates that the SD and the SE of the material properties (e.g., elastic modulus) are not close enough to say whether it can be applied in FEA studies (Appendix 2, Table A1). Finally, it seems that the moulding process of making the mandible replicas creates a marginal error in the composition of the cortical and trabecular bone sections, as observed in our mandible replicas during the PMMT. This means that the biomechanical behaviour of the mandible during mechanical testing may not be identical between each mandible replica. Therefore, in future studies, we aim to use 3D printed mandibles made of a material with known properties that are comparable to real human bone instead of Synbone mandible replicas.

Finally, according to the literature, FEA is a promising tool in the OMF surgery for investigating different types of fracture management and different osteosynthesis systems or implants^6–24,50^. This study shows that our FEA model is an applicable tool for analysing simple mandibular fracture problems; therefore, the outcome of this study is a promising step towards developing a validated FEA model suitable for many different situations (e.g., complex mandibular fractures or other bone fractures in the OMF region).

In conclusion, this study illustrates that the application of the FEA principle in OMF surgery has a lot of protentional. The surgeon can take a leap of faith in the FEA’s capabilities for analysing mandibular fractures (e.g., atrophic or comminuted) as well as for studying or developing new types of osteosynthesis implants (e.g., patient specific 3D printed or biodegradable). However, there is a need for more extensive studies to conclude whether FEA alone is sufficient without having to undertake mechanical testing and whether it can be standardly applied in the clinical setting.

### Supplementary Information

Supplementary Information 1.

Supplementary Information 2.

Supplementary Figure S1.

Supplementary Figure S2.

Supplementary Figure S3.

## Data Availability

Data is provided within the supplementary information files. Furthermore, all data are available from corresponding author upon reasonable request.

## References

[1] Bohner L (2020). Treatment of mandible fractures using a miniplate system: A retrospective analysis. J. Clin. Med..

[2] Champy M, Lodde JP (1976). Synthèses mandibulaires. Localization des synthèses en fonction des contraintes mandibulaires [Mandibular synthesis. Placement of the synthesis as a function of mandibular stress]. Rev. Stomatol. Chir. Maxillofac..

[3] Haerle, F., Champy, M. & Terry, B. C. *Atlas of Craniomaxillofacial Osteosynthesis* (Georg Thieme Verlag, 2009). 10.1055/b-002-72255.

[4] Ehrnfeld, M., Manson, P. N. & Perin, J. *Principles of Internal Fixation of the Craniomaxillofacial Skeleton* (Georg Thieme Verlag, 2012). 10.1055/b-002-85491.

[5] Sittitavornwong S (2018). Integrity of a single superior border plate repair in mandibular angle fracture: A novel cadaveric human mandible model. J. Oral Maxillofac. Surg..

[6] Huang C-M, Chan M-Y, Hsu J-T, Su K-C (2021). Biomechanical analysis of subcondylar fracture fixation using miniplates at different positions and of different lengths. BMC Oral Health.

[7] Trainotti S (2014). Locking versus nonlocking plates in mandibular reconstruction with fibular graft-a biomechanical ex vivo study. Clin. Oral Investig..

[8] Hart RT, Hennebel VV, Thongpreda N, Van Buskirk WC, Anderson RC (1992). Modeling the biomechanics of the mandible: A three-dimensional finite element study. J. Biomech..

[9] Anthrayose P, Nawal RR, Yadav S, Talwar S, Yadav S (2021). Effect of revascularisation and apexification procedures on biomechanical behaviour of immature maxillary central incisor teeth: A three-dimensional finite element analysis study. Clin. Oral Investig..

[10] Park B (2020). The stability of hydroxyapatite/poly-L-lactide fixation for unilateral angle fracture of the mandible assessed using a finite element analysis model. Materials.

[11] Lisiak-Myszke M (2020). Application of finite element analysis in oral and maxillofacial surgery: A literature review. Materials.

[12] Limjeerajarus N (2020). Comparison of ultimate force revealed by compression tests on extracted first premolars and FEA with a true scale 3D multi-component tooth model based on a CBCT dataset. Clin. Oral Investig..

[13] Merema BBJ, Kraeima J, Glas HH, Spijkervet FKL, Witjes MJH (2021). Patient-specific finite element models of the human mandible: Lack of consensus on current set-ups. Oral Dis..

[14] Aftabi H (2024). Computational models and their applications in biomechanical analysis of mandibular reconstruction surgery. Comput. Biol. Med..

[15] Sancar B, Çetiner Y, Day E (2023). Evaluation of the pattern of fracture formation from trauma to the human mandible with finite element analysis. Part 1: Symphysis region. Dent. Traumatol..

[16] Sancar B, Çetiner Y, Day E (2023). Evaluation of the pattern of fracture formation from trauma to the human mandible with finite element analysis. Part 2: The corpus and the angle regions. Dent. Traumatol..

[17] Xue R (2024). Finite element analysis and clinical application of 3D-printed Ti alloy implant for the reconstruction of mandibular defects. BMC Oral Health.

[18] Dario V, Michelangelo-Santo G, Roberto B, Fabio F (2023). Is All-on-four effective in case of partial mandibular resection? A 3D finite element study. J. Stomatol. Oral Maxillofac. Surg..

[19] Maintz M (2023). Parameter optimization in a finite element mandibular fracture fixation model using the design of experiments approach. J. Mech. Behav. Biomed. Mater..

[20] Falcinelli C, Valente F, Vasta M, Traini T (2023). Finite element analysis in implant dentistry: State of the art and future directions. Dental Mater..

[21] Schönegg D, Koch A, Müller GT, Blumer M, Wagner MEH (2023). Two-screw osteosynthesis of the mandibular condylar head with different screw materials: A finite element analysis. Comput. Methods Biomech. Biomed. Eng..

[22] Altuncu F, Kazan D, Özden B (2020). Comperative evaluation of the current and new design miniplate fixation techniques of the advanced sagittal split ramus osteotomy using three-dimensional finite element analysis. Med. Oral Patol. Oral Cir. Bucal..

[23] Gupta A, Dutta A, Dutta K, Mukherjee K (2023). Biomechanical influence of plate configurations on mandible subcondylar fracture fixation: A finite element study. Med. Biol. Eng. Comput..

[24] Daqiq O, Roossien CC, Wubs FW, Bos RRM, van Minnen B (2023). Optimisation of osteosynthesis positioning in mandibular body fracture management using finite element analysis. Eur. J. Transl. Clin. Med..

[25] Rentes AM, Gavião MBD, Amaral JR (2002). Bite force determination in children with primary dentition. J. Oral Rehabil..

[26] Ahmed S (2016). A comparative study on evaluation of role of 1.5 mm microplates and 2.0 mm standard miniplates in management of mandibular fractures using bite force as indicator of recommendation. Natl. J. Maxillofac. Surg..

[27] Schupp W, Arzdorf M, Linke B, Gutwald R (2007). Biomechanical testing of different osteosynthesis systems for segmental resection of the mandible. J. Oral Maxillofac. Surg..

[28] Kumar S (2014). Comparative evaluation of bite forces in patients after treatment of mandibular fractures with miniplate osteosynthesis and internal locking miniplate osteosynthesis. J. Int. Soc. Prev. Community Dent..

[29] Aymach Z, Nei H, Kawamura H, Bell W (2011). Biomechanical evaluation of a T-shaped miniplate fixation of a modified sagittal split ramus osteotomy with buccal step, a new technique for mandibular orthognathic surgery. Oral Surg. Oral Med. Oral Pathol. Oral Radiol. Endodontol..

[30] Bredbenner TL, Haug RH (2000). Substitutes for human cadaveric bone in maxillofacial rigid fixation research. Oral Surg. Oral Med. Oral Pathol. Oral Radiol. Endodontol..

[31] Brown AD (2019). The mechanical response of commercially available bone simulants for quasi-static and dynamic loading. J. Mech. Behav. Biomed. Mater..

[32] Zdero R, Djuricic A, Schemitsch EH (2023). Mechanical properties of synthetic bones made by synbone: A review. J. Biomech. Eng..

[33] Park Y-C (2021). Comparative pull-out performances of cephalomedullary nail with screw and helical blade according to femur bone densities. Appl. Sci..

[34] Gareb B (2020). Comparison of the mechanical properties of biodegradable and titanium osteosynthesis systems used in oral and maxillofacial surgery. Sci. Rep..

[35] Michelet FX, Deymes J, Dessus B (1973). Osteosynthesis with miniaturized screwed plates in maxillo-facial surgery. J. Maxillofac. Surg..

[36] Kroon FHM, Mathisson M, Cordey JR, Rahn BA (1991). The use of miniplates in mandibular fractures. J. Cranio-Maxillofac. Surg..

[37] Choi BH, Yoo JH, Kim KN, Kang HS (1995). Stability testing of a two miniplate fixation technique for mandibular angle fractures. An in vitro study. J. Cranio-Maxillofac. Surg..

[38] Braasch DC, Abubaker AO (2013). Management of mandibular angle fracture. Oral Maxillofac. Surg. Clin. North Am..

[39] Madsen MJ, McDaniel CA, Haug RH (2008). A biomechanical evaluation of plating techniques used for reconstructing mandibular symphysis/parasymphysis fractures. J. Oral Maxillofac. Surg..

[40] Raut R, Keerthi R, Vaibhav N, Ghosh A, Kamath Kateel S (2017). Single miniplate fixation for mandibular symphysis and parasymphysis fracture as a viable alternative to conventional plating based on Champy’s principles: A prospective comparative clinical study. J. Maxillofac. Oral Surg..

[41] Tams J, van Loon J-P, Otten E, Rozema FR, Bos RRM (1997). A three-dimensional study of bending and torsion moments for different fracture sites in the mandible: An in vitro study. Int. J. Oral Maxillofac. Surg..

[42] Siddiqui A, Markose G, Moos KF, McMahon J, Ayoub AF (2007). One miniplate versus two in the management of mandibular angle fractures: A prospective randomised study. Br. J. Oral Maxillofac. Surg..

[43] Gear AJL, Apasova E, Schmitz JP, Schubert W (2005). Treatment modalities for mandibular angle fractures. J. Oral Maxillofac. Surg..

[44] Lloyd JE, Tavares JMRS, Fernandes PR (2019). New techniques for combined FEM-multibody anatomical simulation. New Developments on Computational Methods and Imaging in Biomechanics and Biomedical Engineering.

[45] Rzymkowski C (2000). “Hybrid” approach to modelling of biomechanical systems. Human Biomechanics and Injury Prevention.

[46] Nispel K, Lerchl T, Senner V, Kirschke JS (2023). Recent advances in coupled MBS and FEM models of the spine: A review. Bioengineering.

[47] Putame G, Pascoletti G, Terzini M, Zanetti EM, Audenino AL (2020). Mechanical behavior of elastic self-locking nails for intramedullary fracture fixation: A numerical analysis of innovative nail designs. Front. Bioeng. Biotechnol..

[48] Koper DC (2021). Topology optimization of a mandibular reconstruction plate and biomechanical validation. J. Mech. Behav. Biomed. Mater..

[49] van Kootwijk A (2022). Semi-automated digital workflow to design and evaluate patient-specific mandibular reconstruction implants. J. Mech. Behav. Biomed. Mater..

[50] Patussi C (2019). Evaluation of different stable internal fixation in unfavorable mandible fractures under finite element analysis. Oral Maxillofac. Surg..

