# Supplementary material for: Biomechanical assessment of mandibular fracture fixation using finite element analysis validated by polymeric mandible mechanical testing
Source: Sci Rep. 2024 May 23;14:11795. doi: 10.1038/s41598-024-62011-4 (PMC11116419; doi:10.1038/s41598-024-62011-4)
Supplement: Supplementary file 1 — Supplementary Information 1. [file 41598_2024_62011_MOESM1_ESM.docx]

**Appendix 1: Polymeric mandible mechanical testing (PMMT) outcomes at the failure point.**

**Appendix 1 Table A1.** Polymeric mandible mechanical testing (PMMT) displacement at the failure force.

| **Mandibular fracture** | **Miniplate configuration** | **Test number*** | **Failure force [N]** | **Displacement [mm]**  **at failure force** |
| --- | --- | --- | --- | --- |
| **Symphysis** | **Superior** | 1 | 504 | 12.48 |
|  |  | 2 | 429 | 14.31 |
|  |  | 3 | 507 | 16.89 |
|  |  | *Mean* | *480* | *14.56* |
|  | **Inferior** | 1 | 381 | 10.03 |
|  |  | 2 | 525 | 12.68 |
|  |  | 3 | 425 | 11.59 |
|  |  | *Mean* | *443.67* | *11.43* |
|  | **Two-plate** | 1 | 467 | 12.38 |
|  |  | 2 | 433 | 12.27 |
|  |  | 3 | 522 | 12.80 |
|  |  | *Mean* | *474* | *12.48* |
| **Parasymphysis** | **Superior** | 1 | 455 | 14.82 |
|  |  | 2 | 403 | 11.85 |
|  |  | 3 | 435 | 14.37 |
|  |  | *Mean* | *431* | *13.68* |
|  | **Inferior** | 1 | 395 | 12.89 |
|  |  | 2 | 350 | 13.96 |
|  |  | 3 | 454 | 16.06 |
|  |  | *Mean* | *399.67* | *14.30* |
|  | **Two-plate** | 1 | 400 | 9.41 |
|  |  | 2 | 508 | 15.44 |
|  |  | 3 | 464 | 12.62 |
|  |  | *Mean* | *457.33* | *12,49* |
| **Angle** | **Superior** | 1 | 419 | 14.25 |
|  |  | 2 | 402 | 11.93 |
|  |  | 3 | 333 | 12.70 |
|  |  | *Mean* | *384.67* | *12.96* |
|  | **Inferior** | 1 | 245 | 9.82 |
|  |  | 2 | 307 | 13.91 |
|  |  | 3 | 250 | 10.13 |
|  |  | *Mean* | *267.33* | *11.29* |
|  | **Two-plate** | 1 | 384 | 12.41 |
|  |  | 2 | 410 | 13.88 |
|  |  | 3 | 449 | 14.08 |
|  |  | *Mean* | *414.33* | *13.46* |

* For the PMMT: each plate configuration was repeated three times for each fracture under exact conditions (Test numbers 1-3).

*Italics*: mean values.

| **a1**  **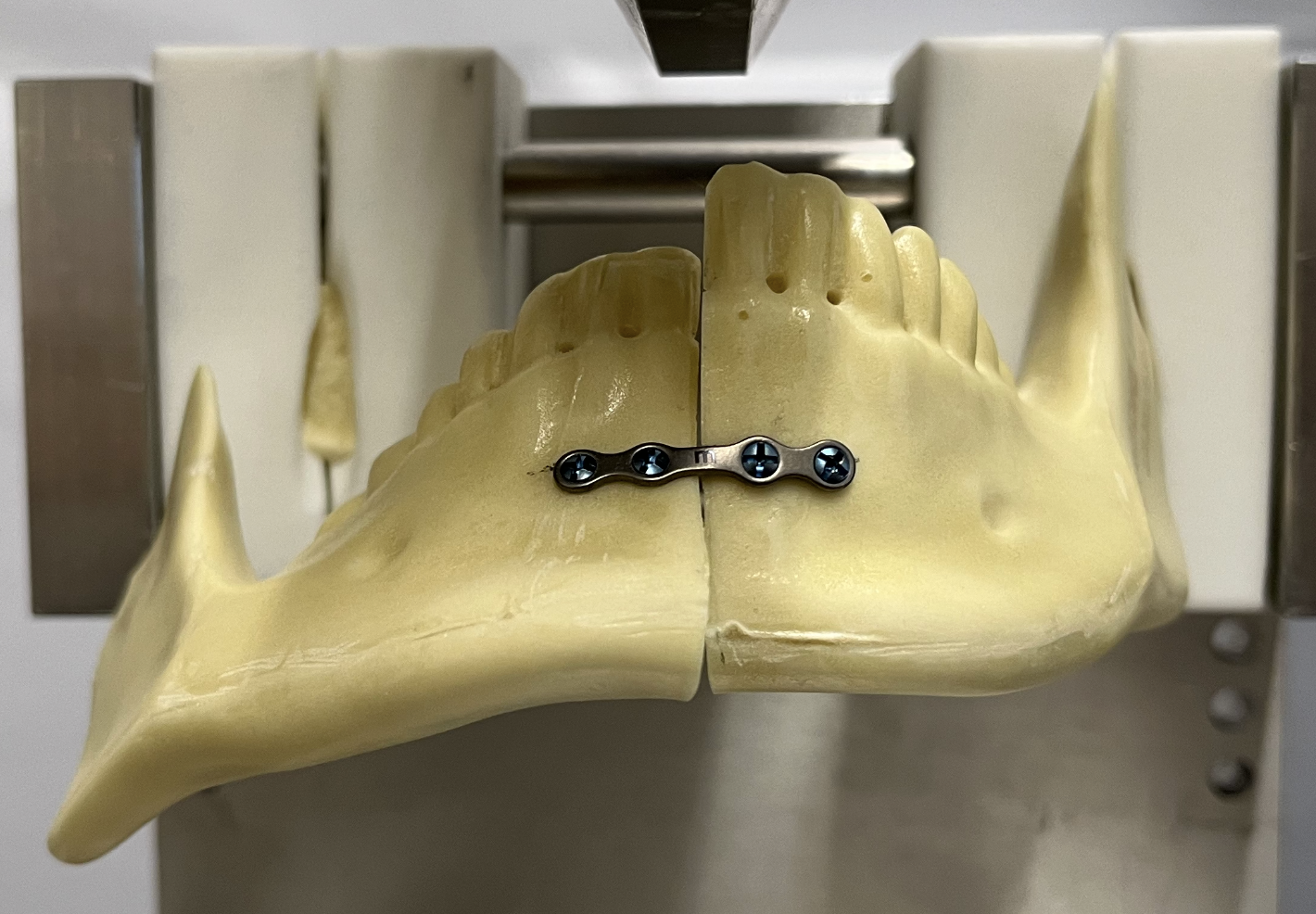** | **a2**  **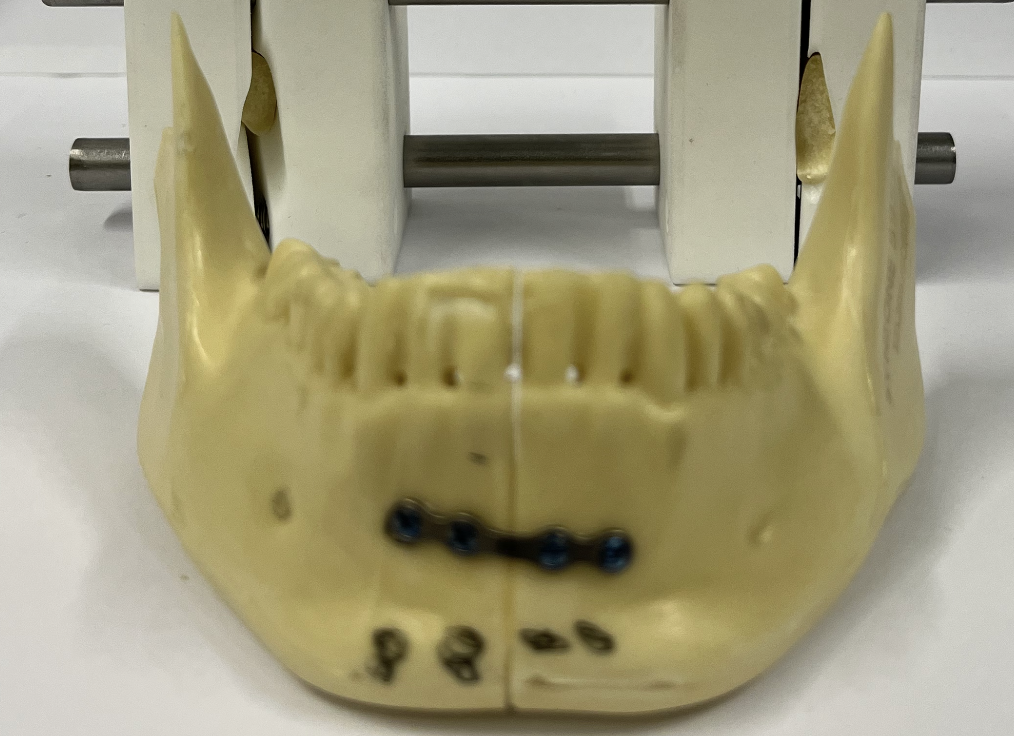** | **a3**  **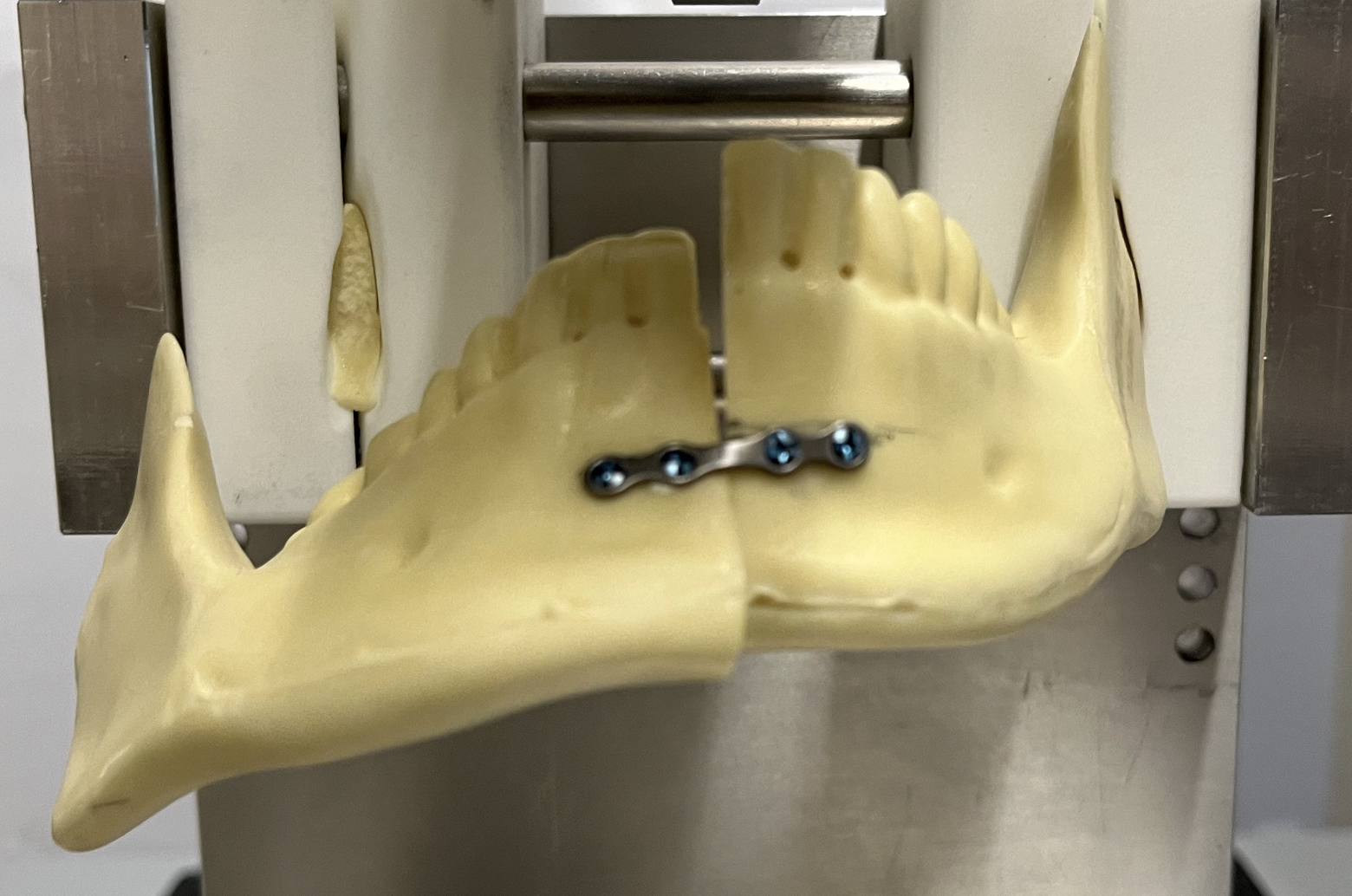** |
| --- | --- | --- |
| **b1**  **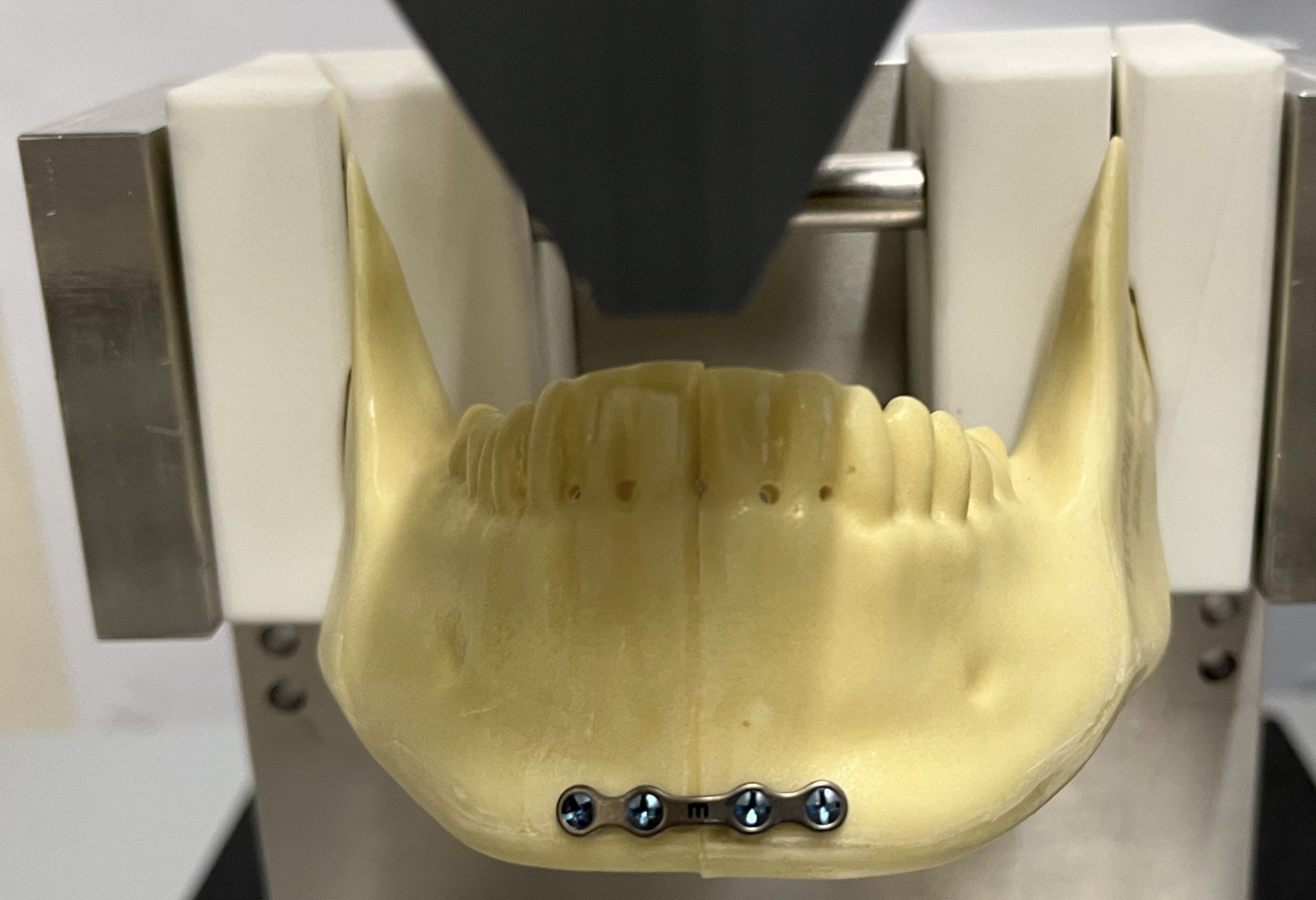** | **b2**  **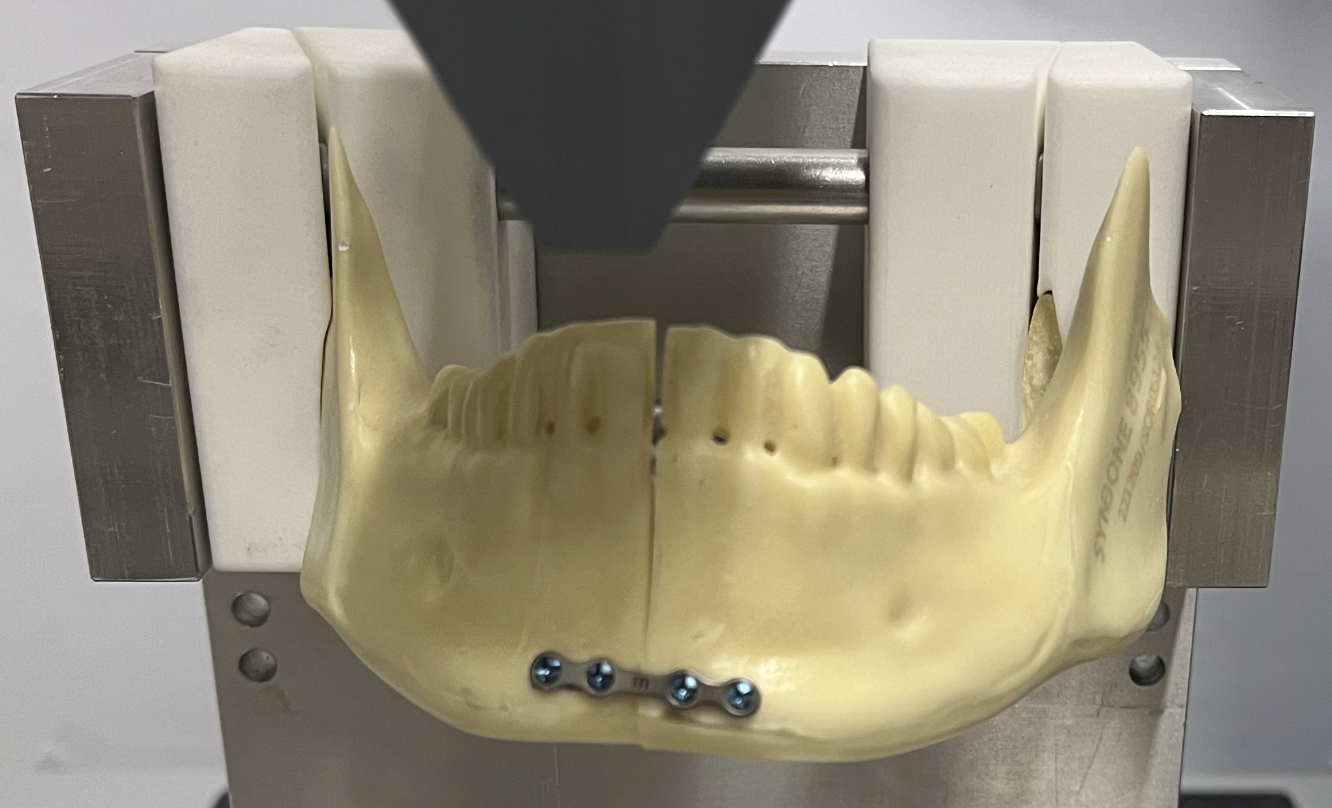** | **b3**  **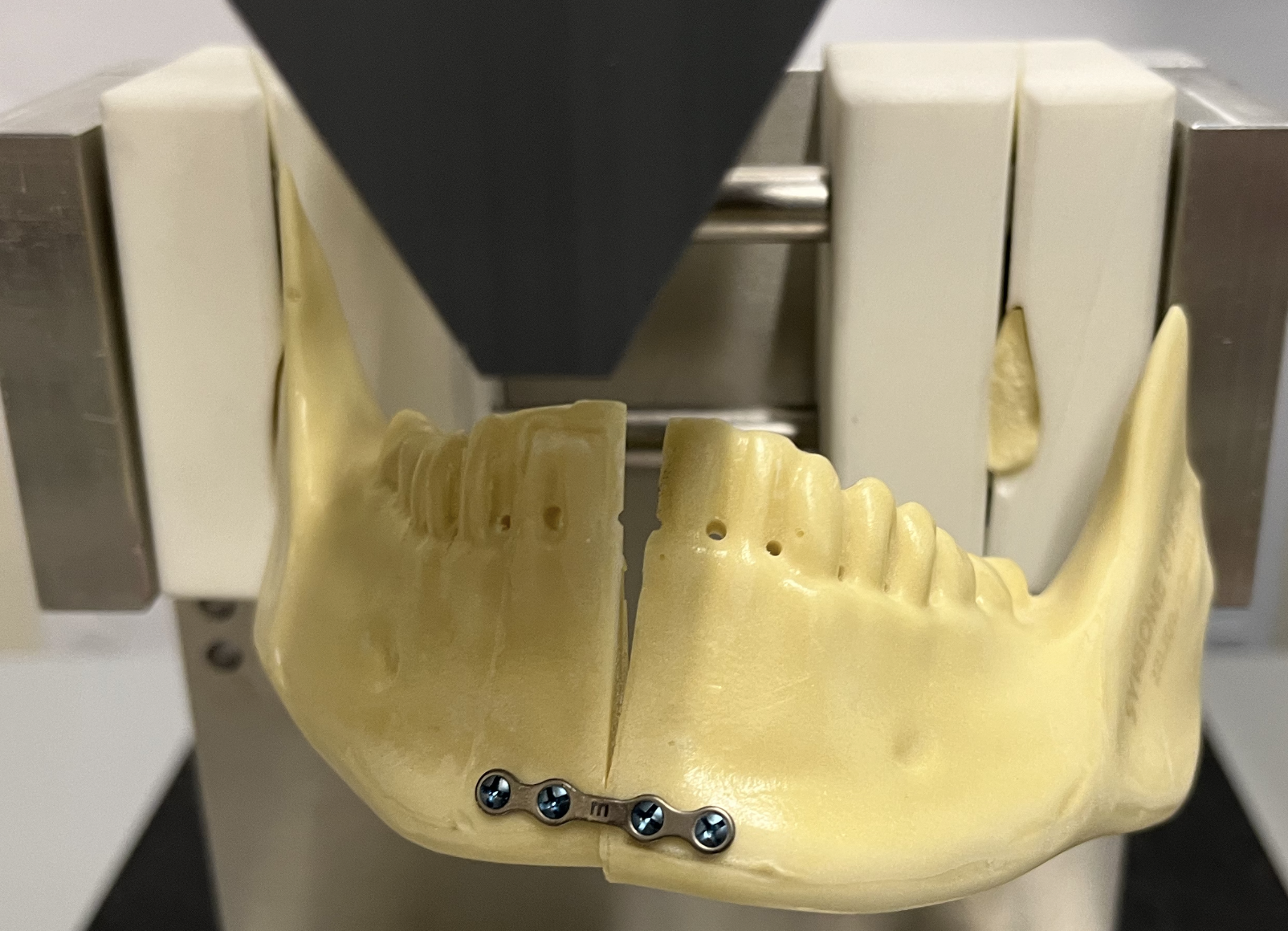** |
| **c1**  **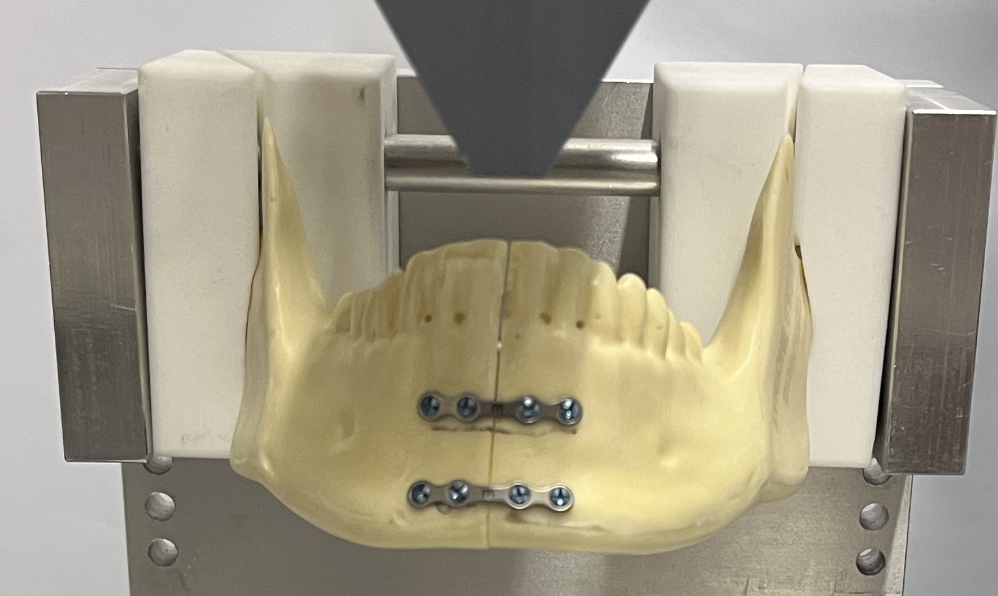** | **c2**  **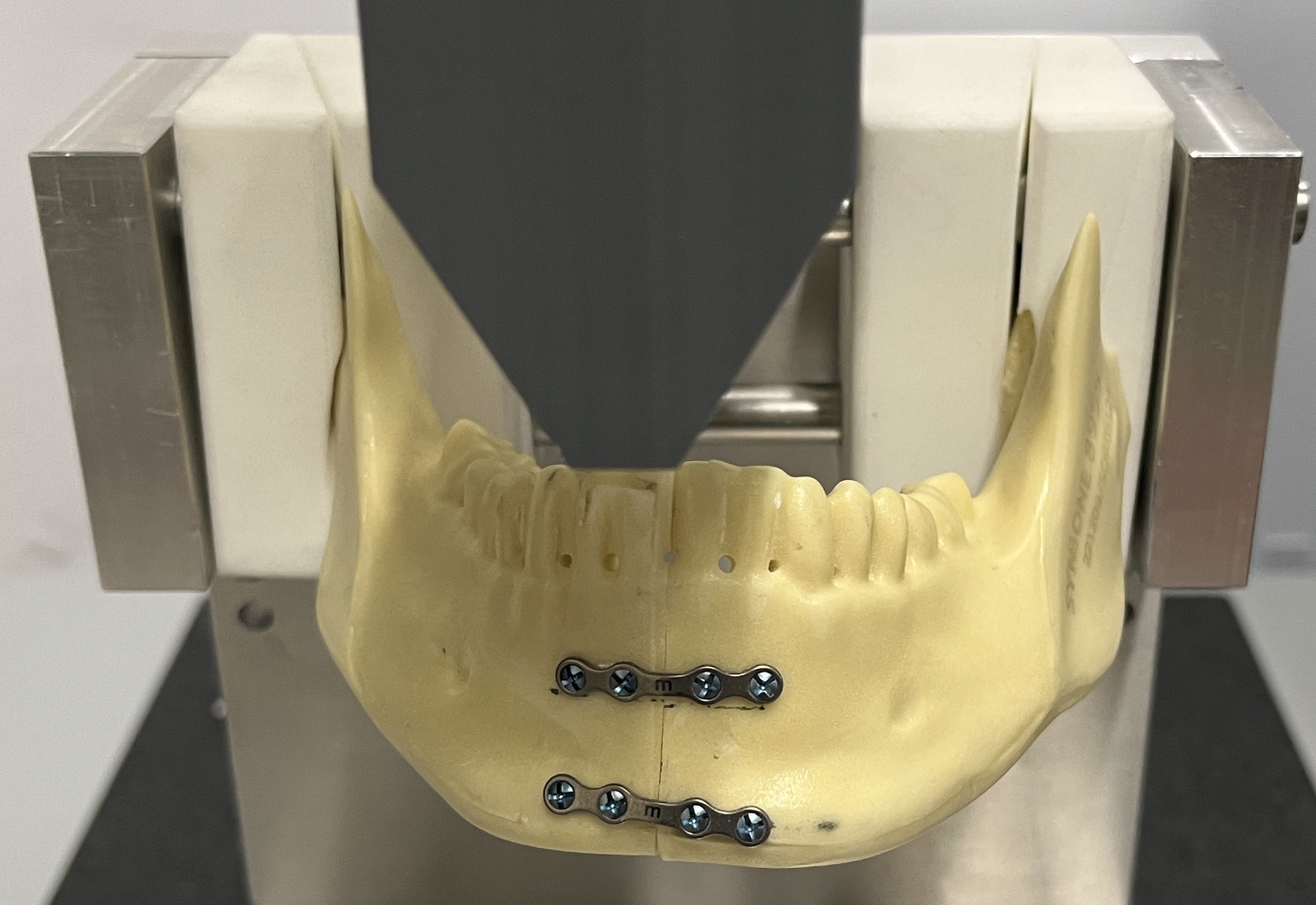** | **c3**  **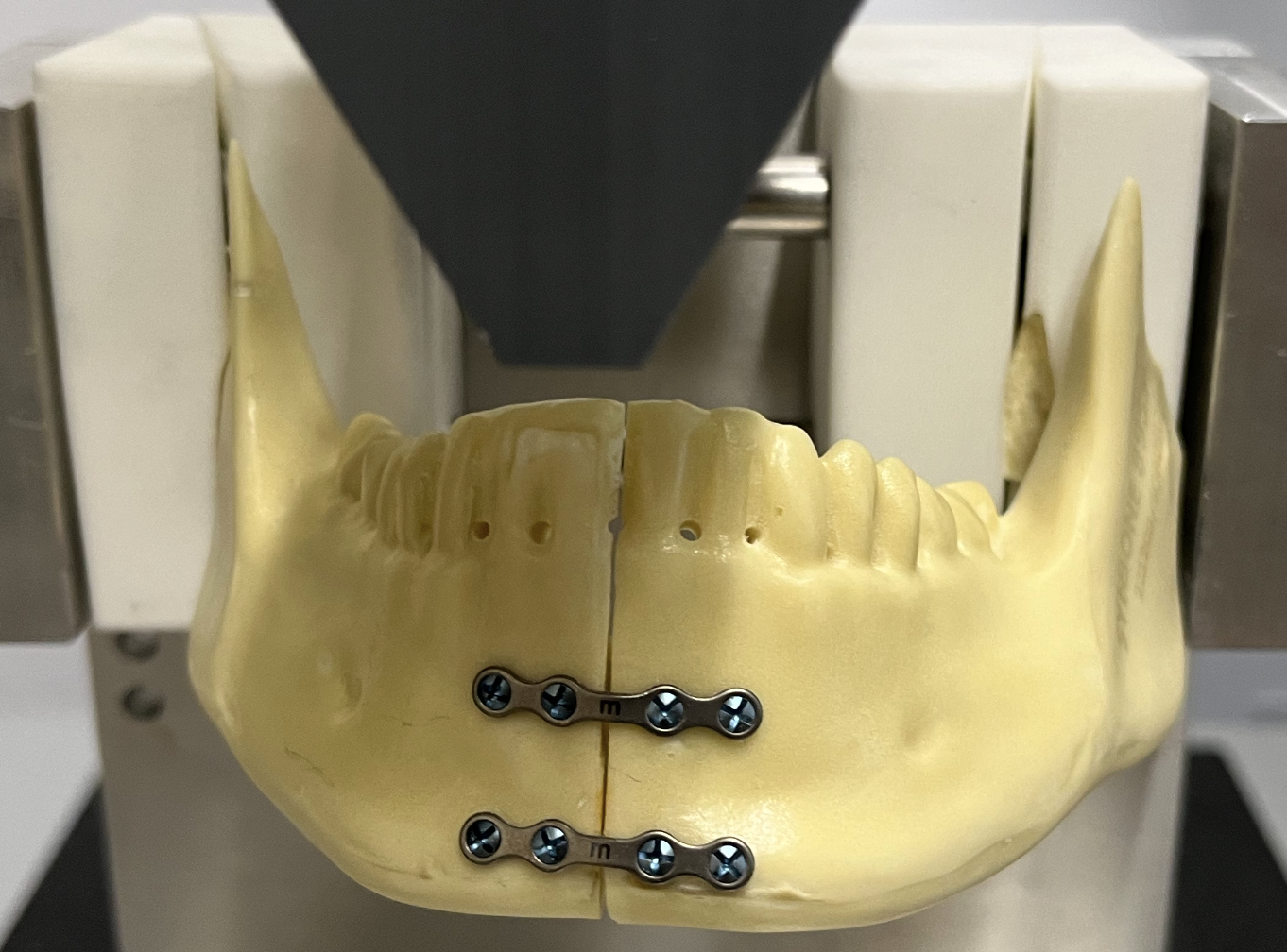** |

**Appendix 1 Figure A1.** The break pattern of mandible replicas with a symphysis fracture at the peak maximum force.

In all cases, the mandible broke at the fixated side where the mandible was fixed using the 3D printed Nylon (polyamide type 12) mandible holders inside the mechanical test bench. In all cases, the miniplate and screws remained intact on the mandible.

(**a**) Superior miniplate configuration: (a1, a2) broke on the right side (a1, a3), and (a2) broke on both fixation sides with the mandible holders.

(**b**) Inferior miniplate configuration: all the mandibles broke on the left side where the mandible was fixated by the 3D printed mandible holders inside the mechanical test bench

(**c**) Two miniplate configuration: all the mandibles broke on the left side where the mandible was fixated by the 3D printed mandible holders inside the mechanical test bench

| **a1**  **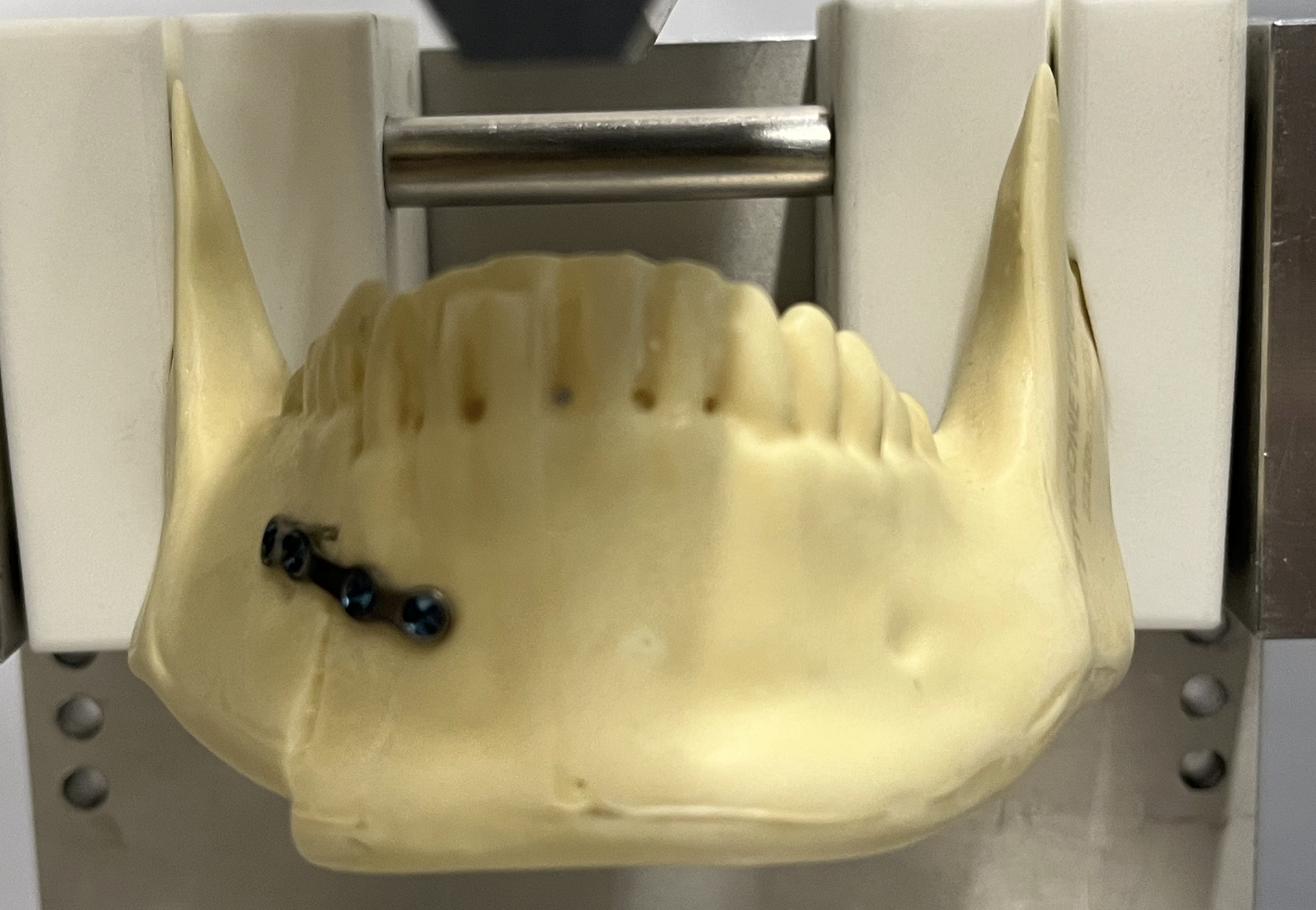** | **a2**  **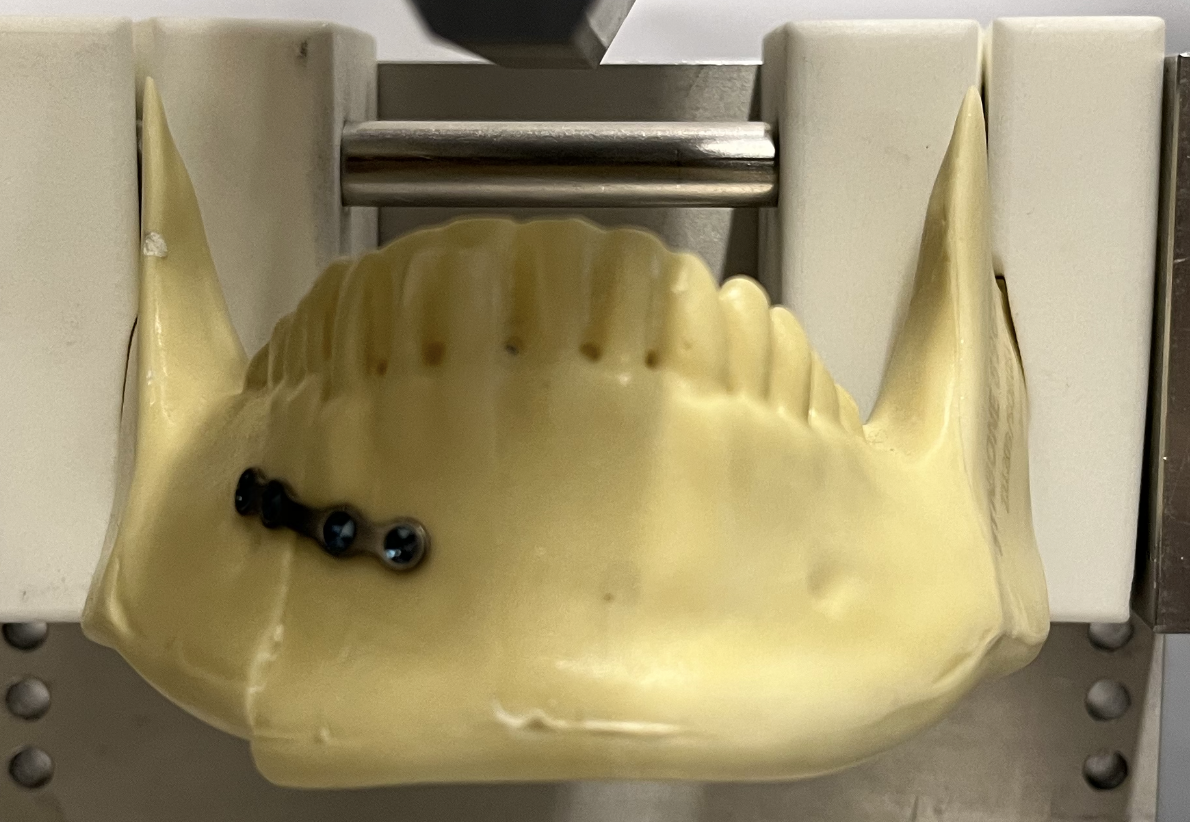** | **a3**  **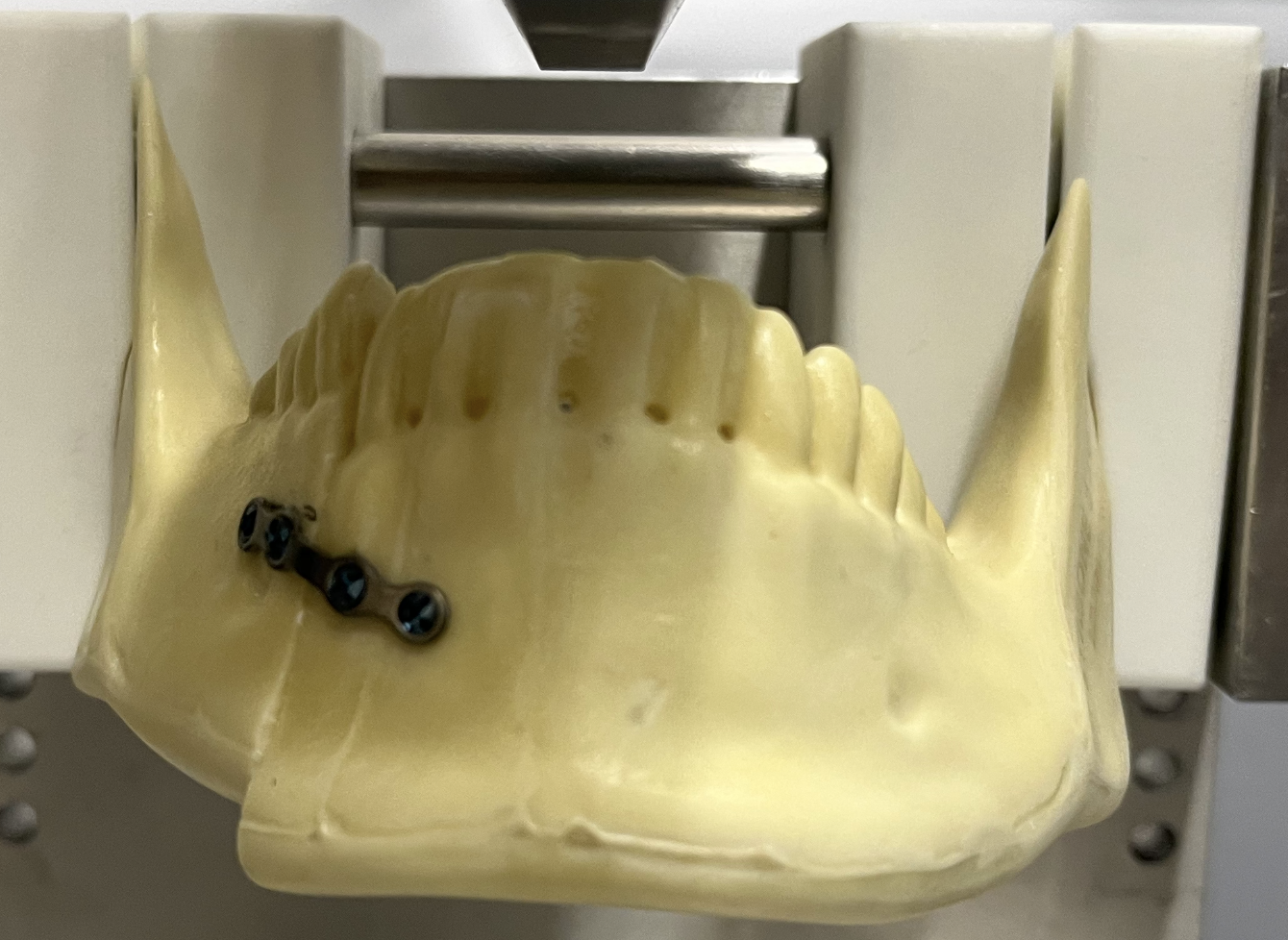** |
| --- | --- | --- |
| **b1**  **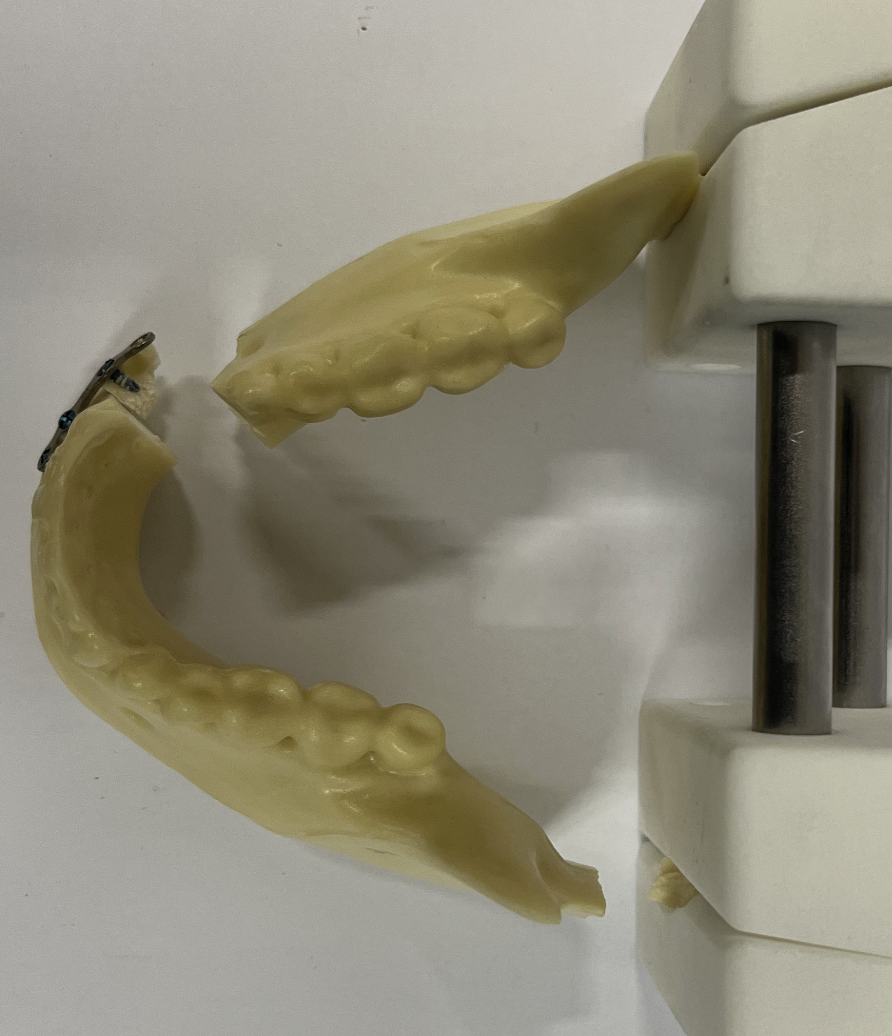** | **b2**  **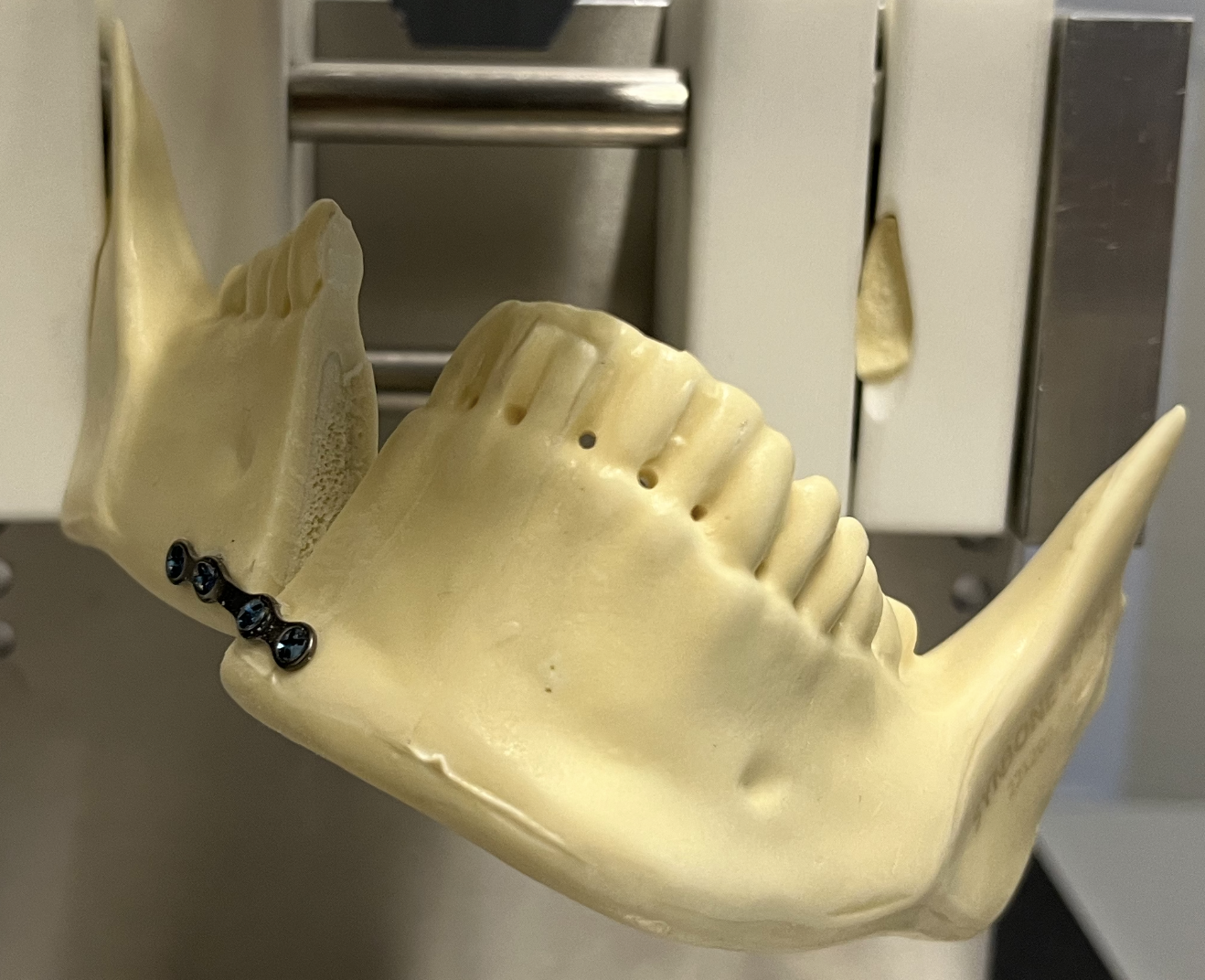** | **b3**  **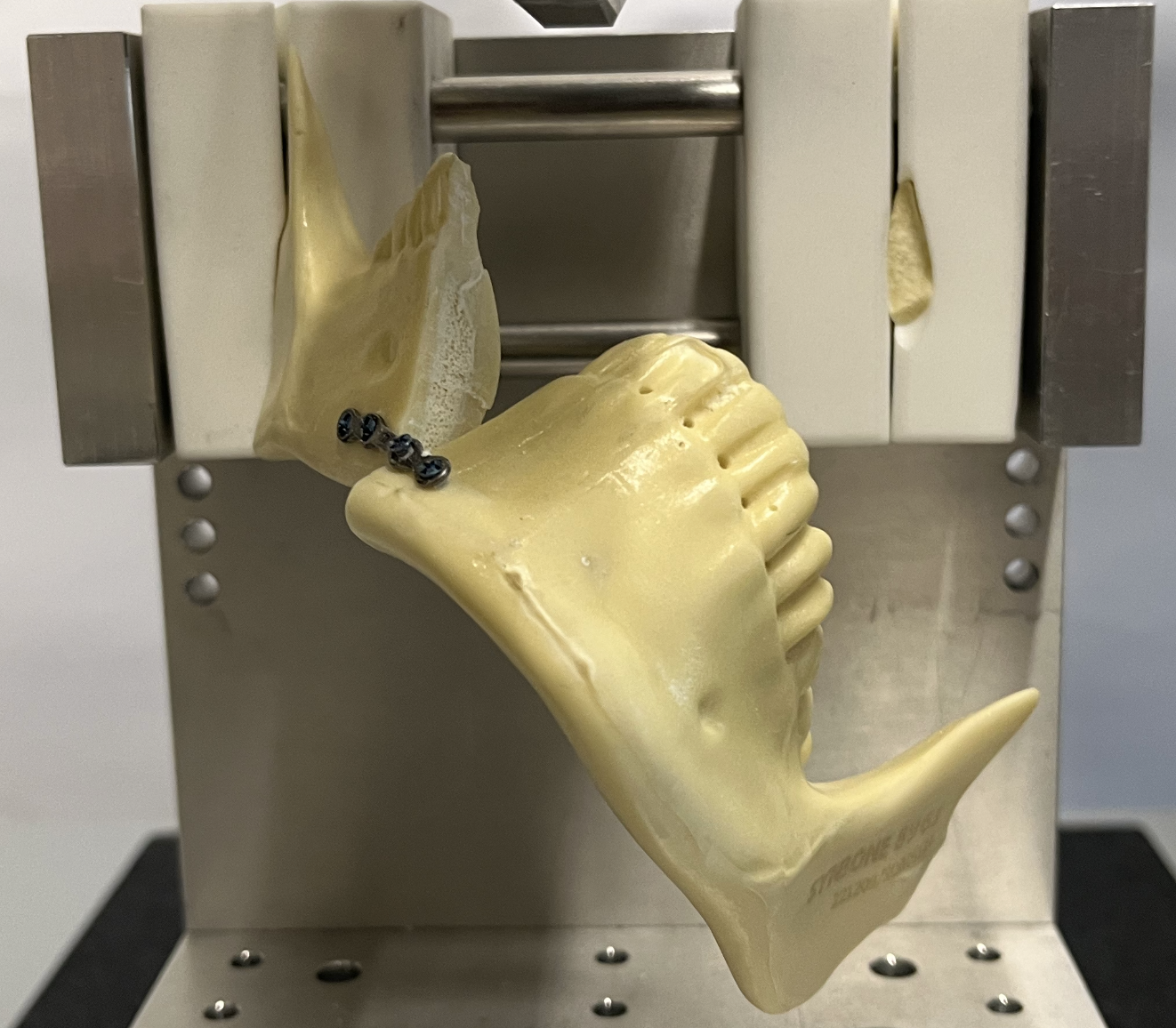** |
| **c1**  **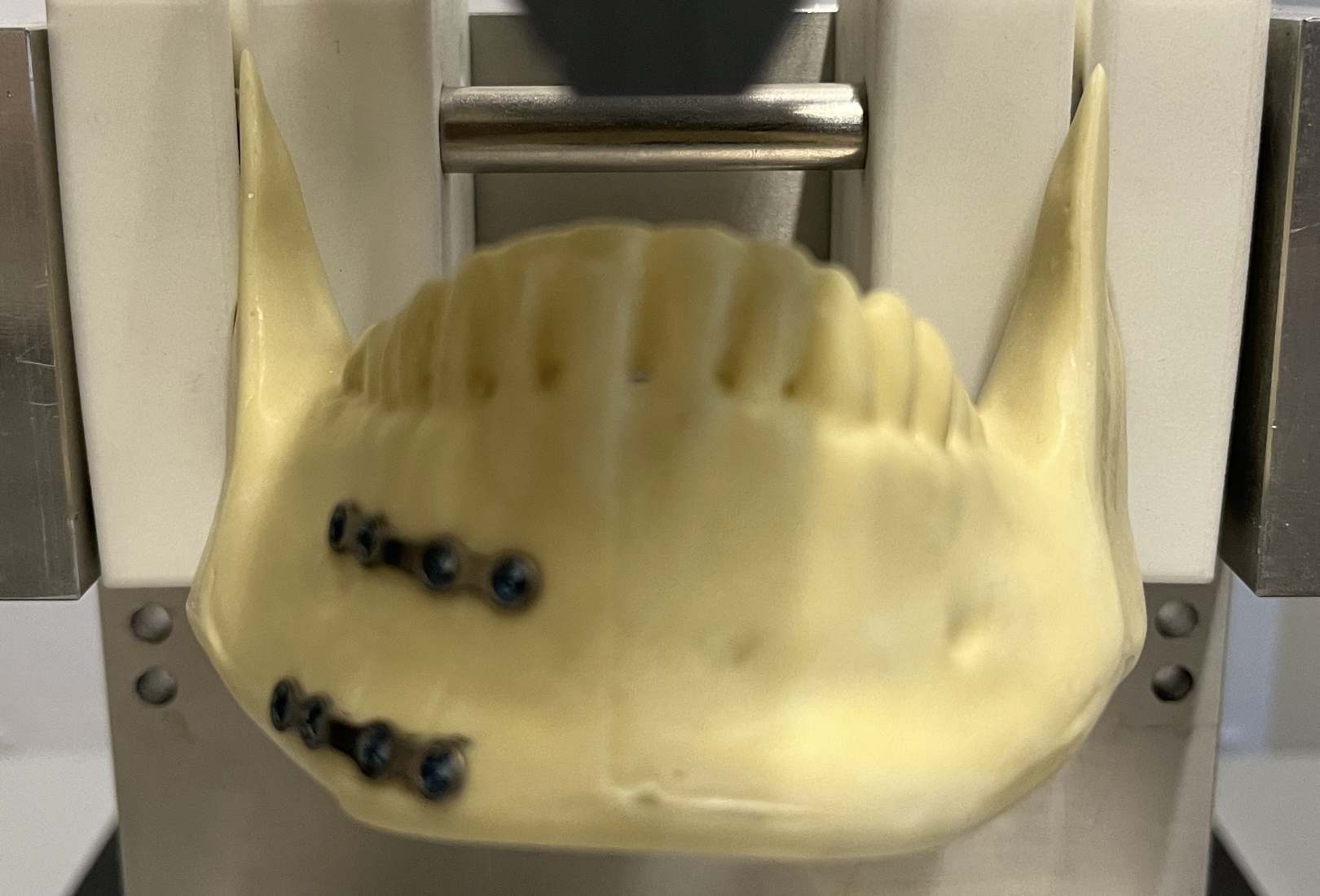** | **c2**  **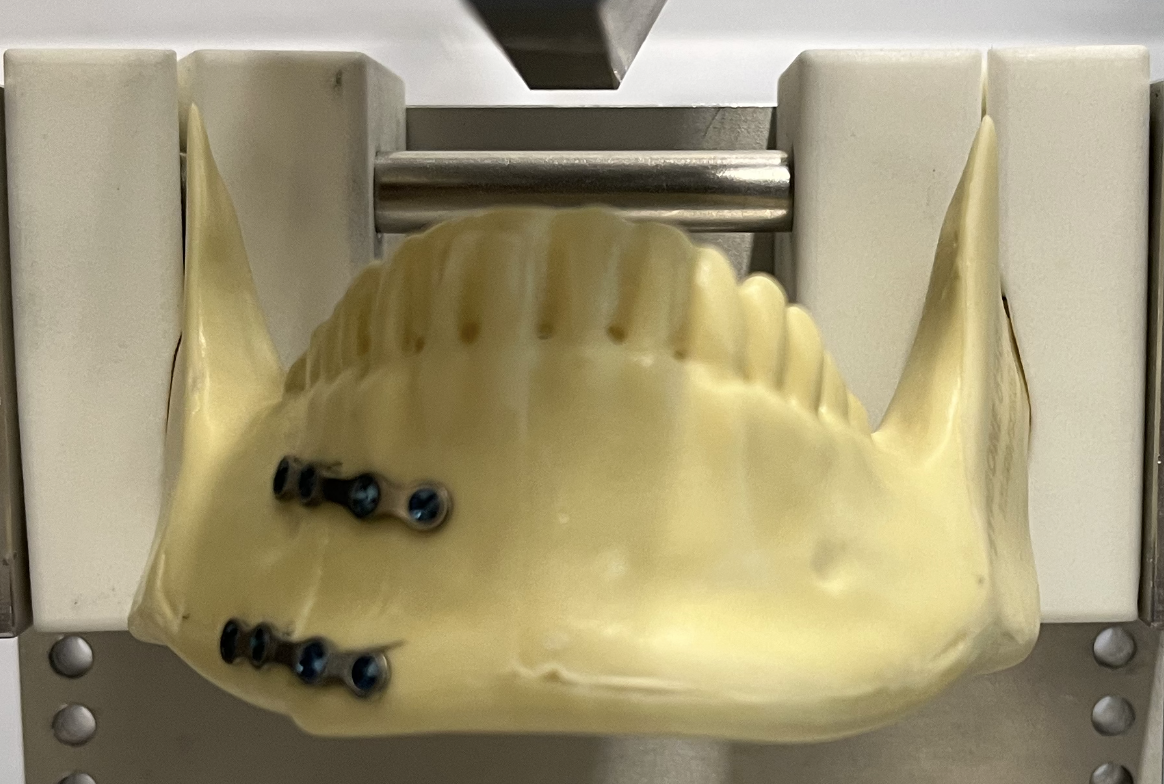** | **c3**  **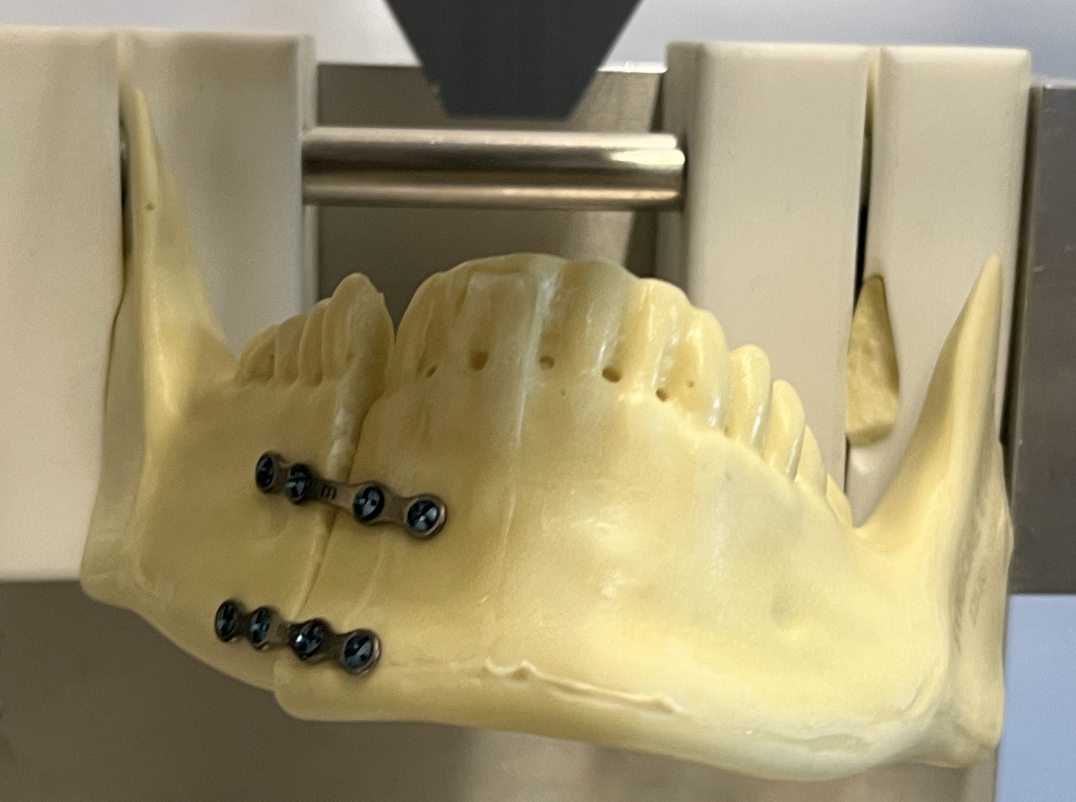** |

**Appendix 1 Figure A2.** The break pattern of mandible replicas with a parasymphysis fracture at the peak maximum force.

(**a**) Superior miniplate configuration: all the mandibles broke on the left fixated side where the mandible was fixated by the 3D printed mandible holders inside the mechanical test bench.

(**b**) Inferior miniplate configuration: all the mandibles broke on the left fixated side where the mandible was fixated by the 3D printed mandible holders inside the mechanical test bench. (b1) additional breakage at the parasymphysis fracture site into three fragments, and one screw was lost.

(**c**) Two miniplate configuration: all the mandibles broke on the left fixated side where the mandible was fixated by the 3D printed mandible holders inside the mechanical test bench.

| **a1**  **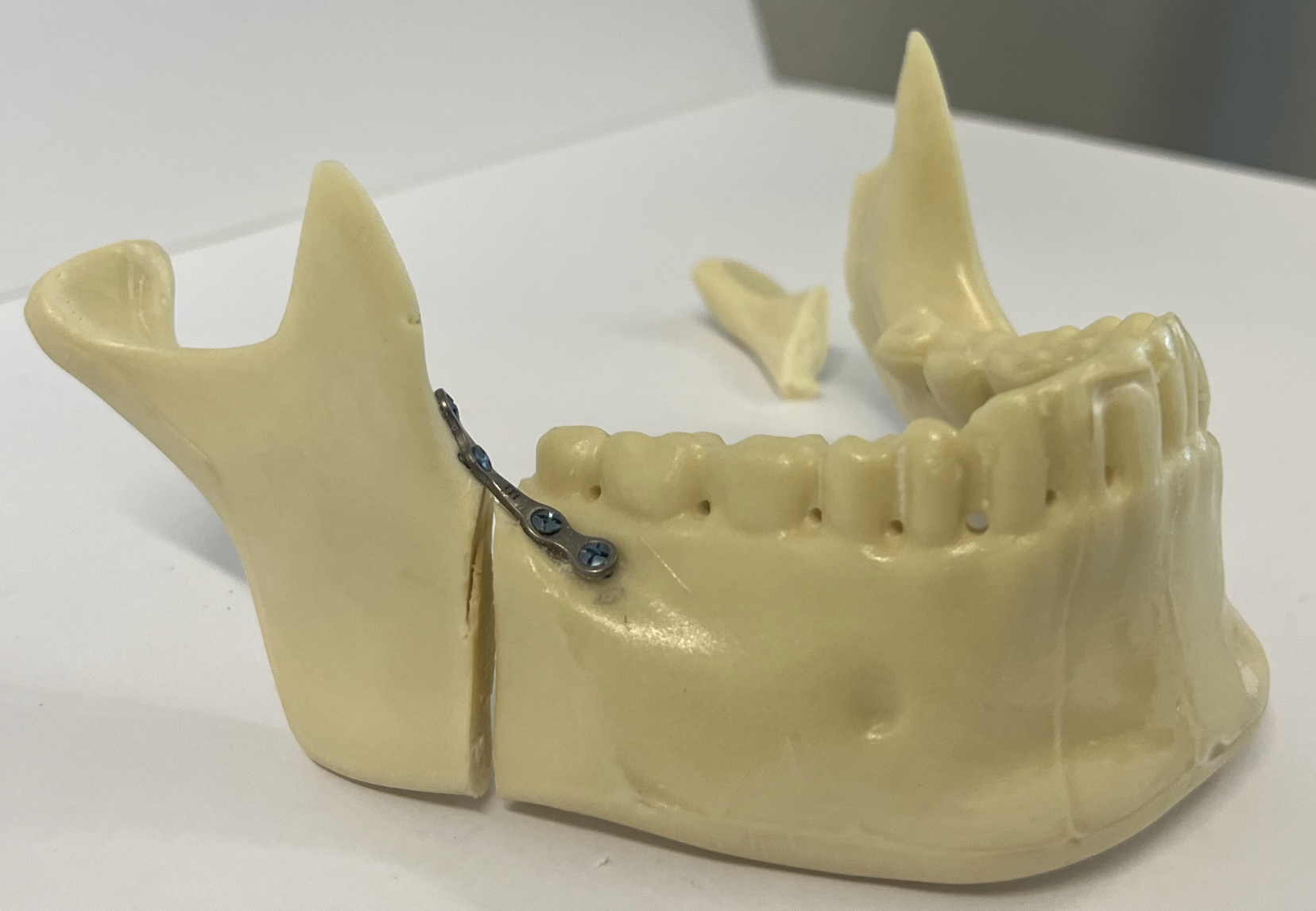** | **a2**  **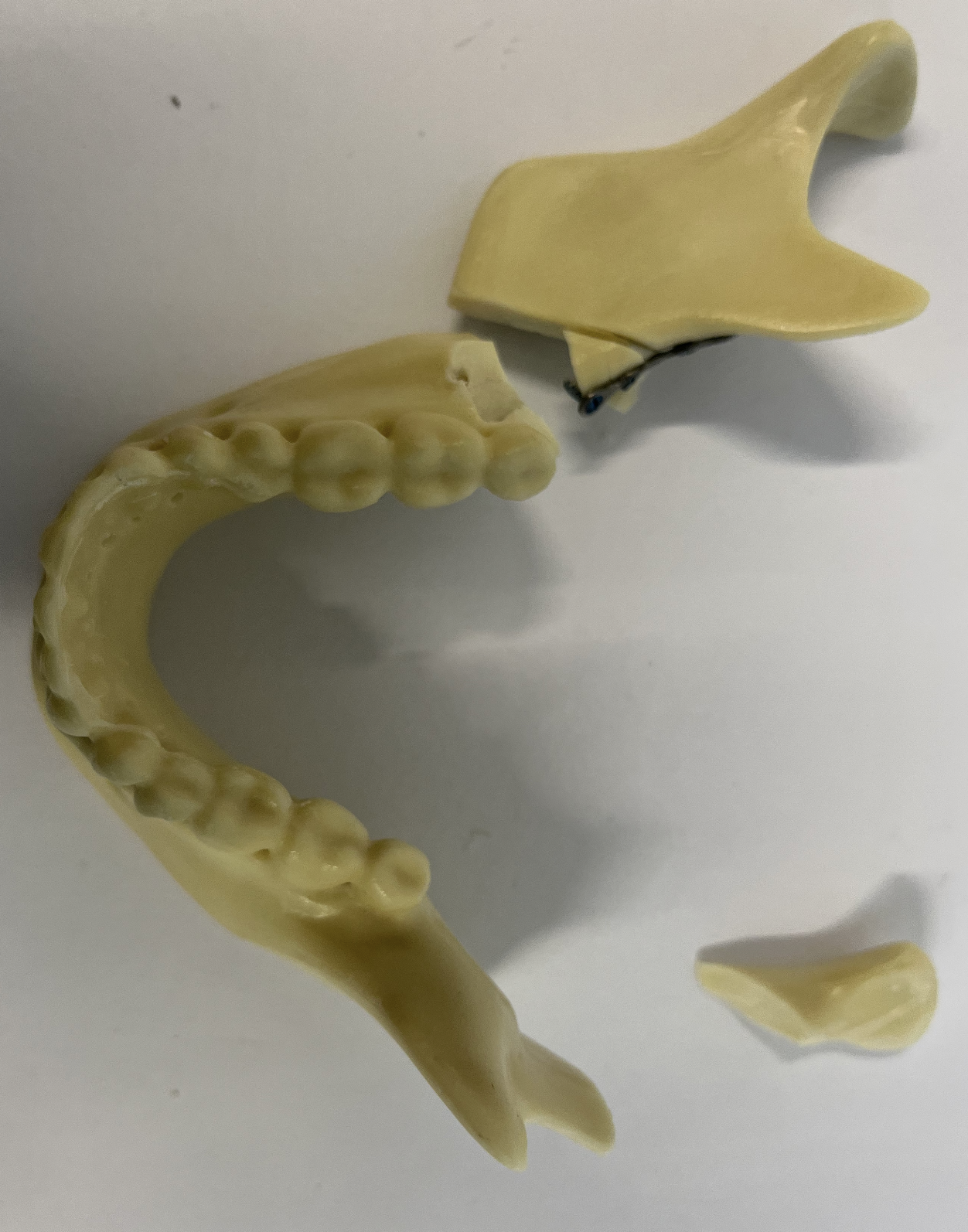** | **a3**  **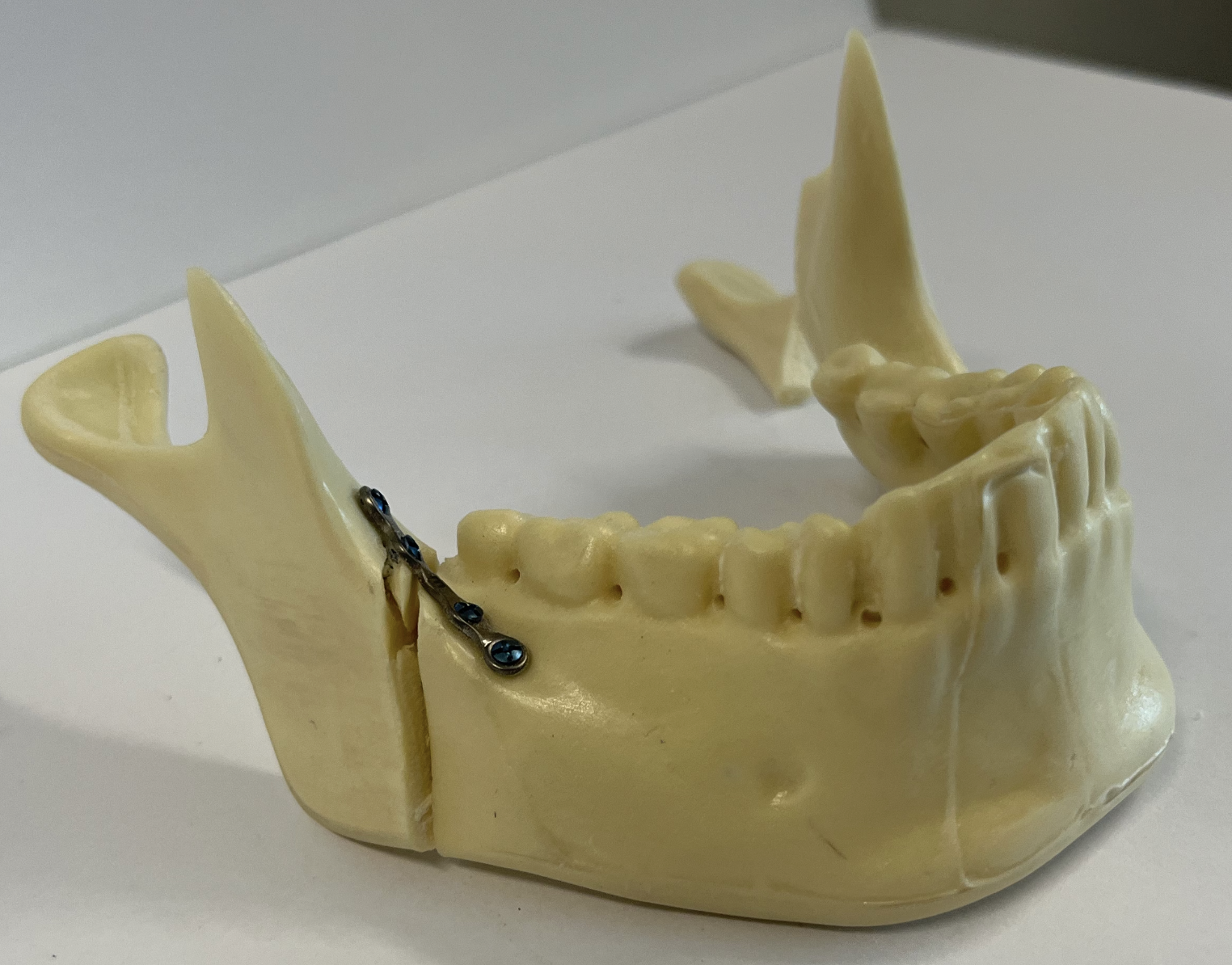** |
| --- | --- | --- |
| **b1**  **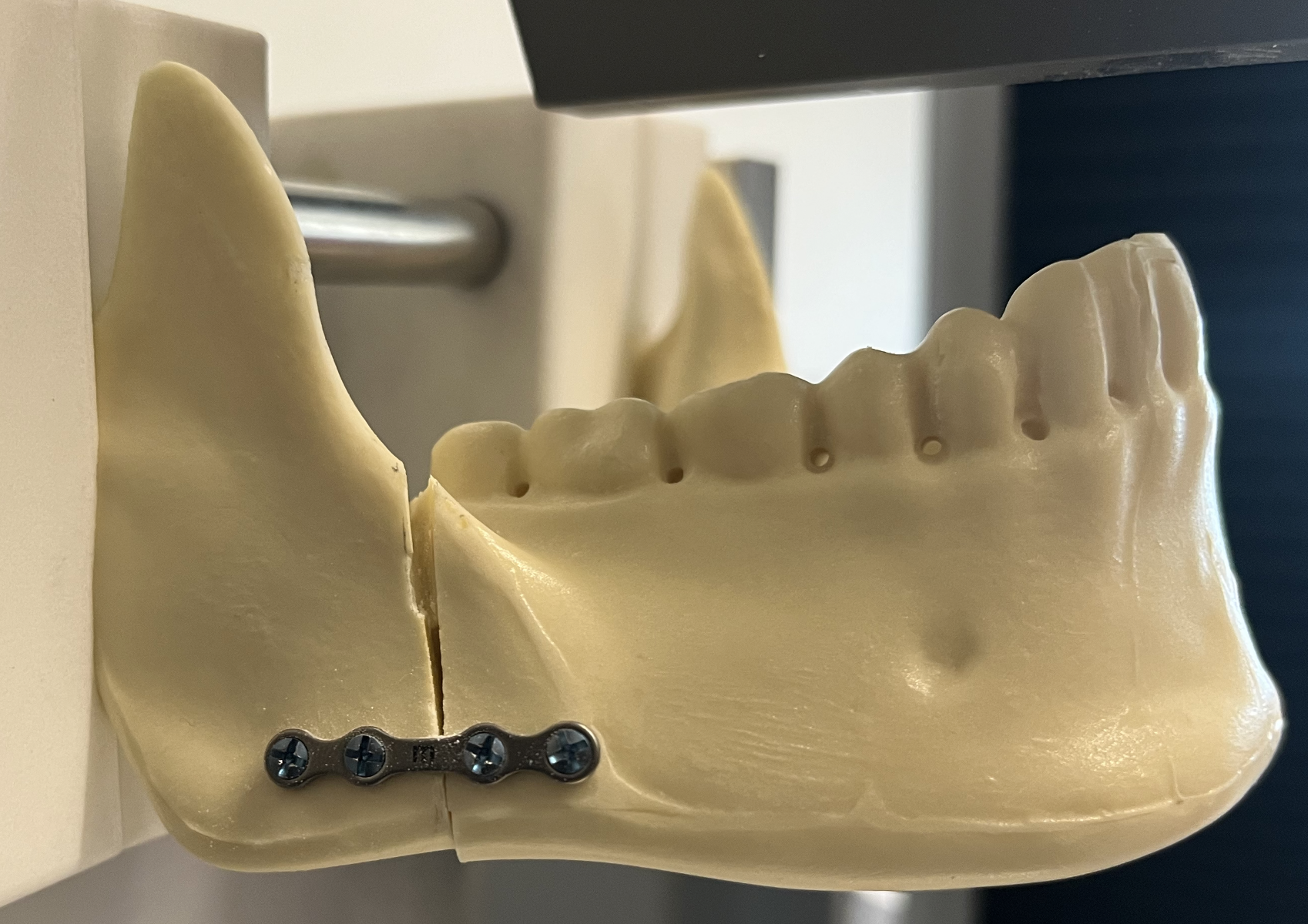** | **b2**  **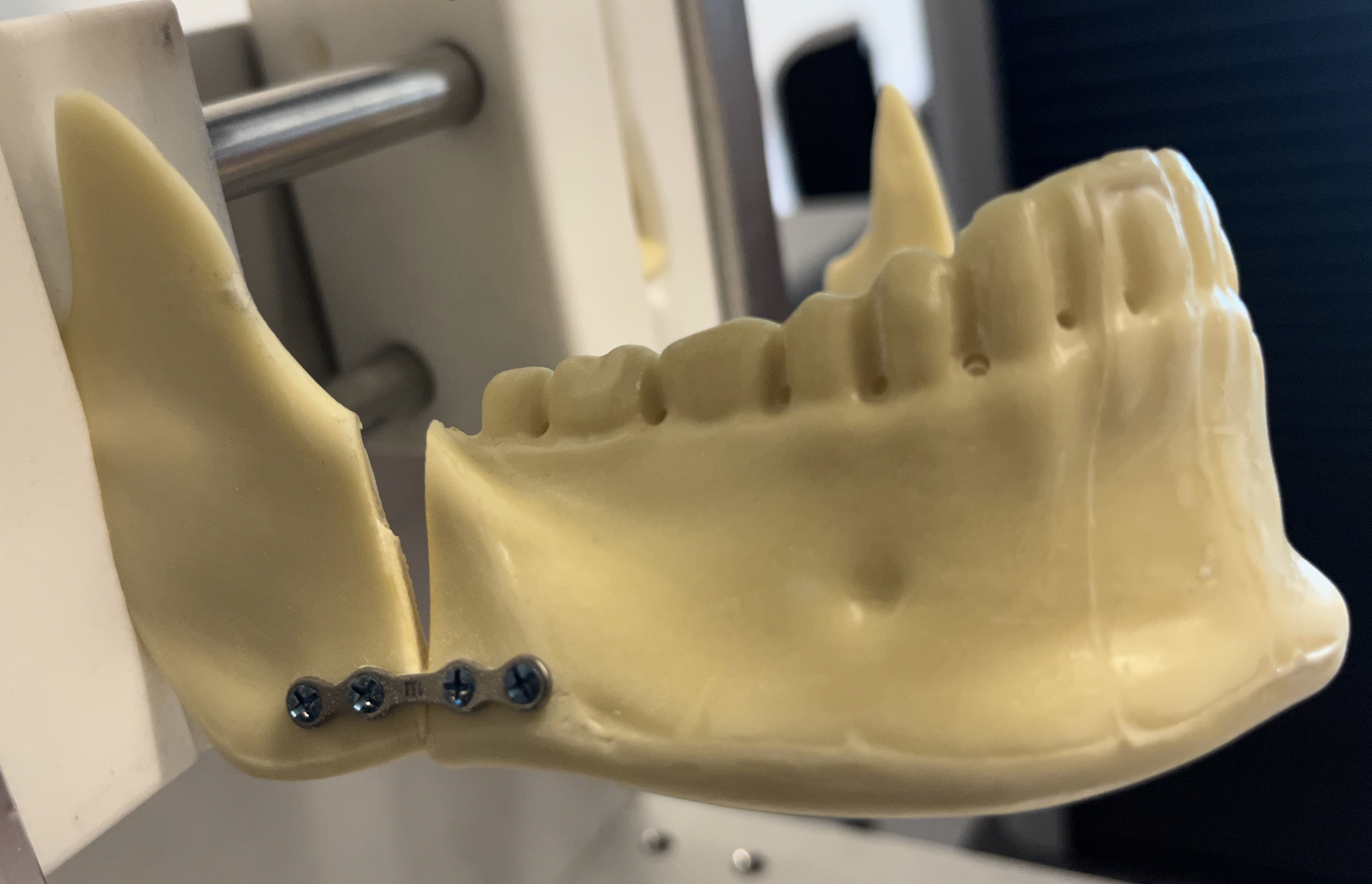** | **b3**  **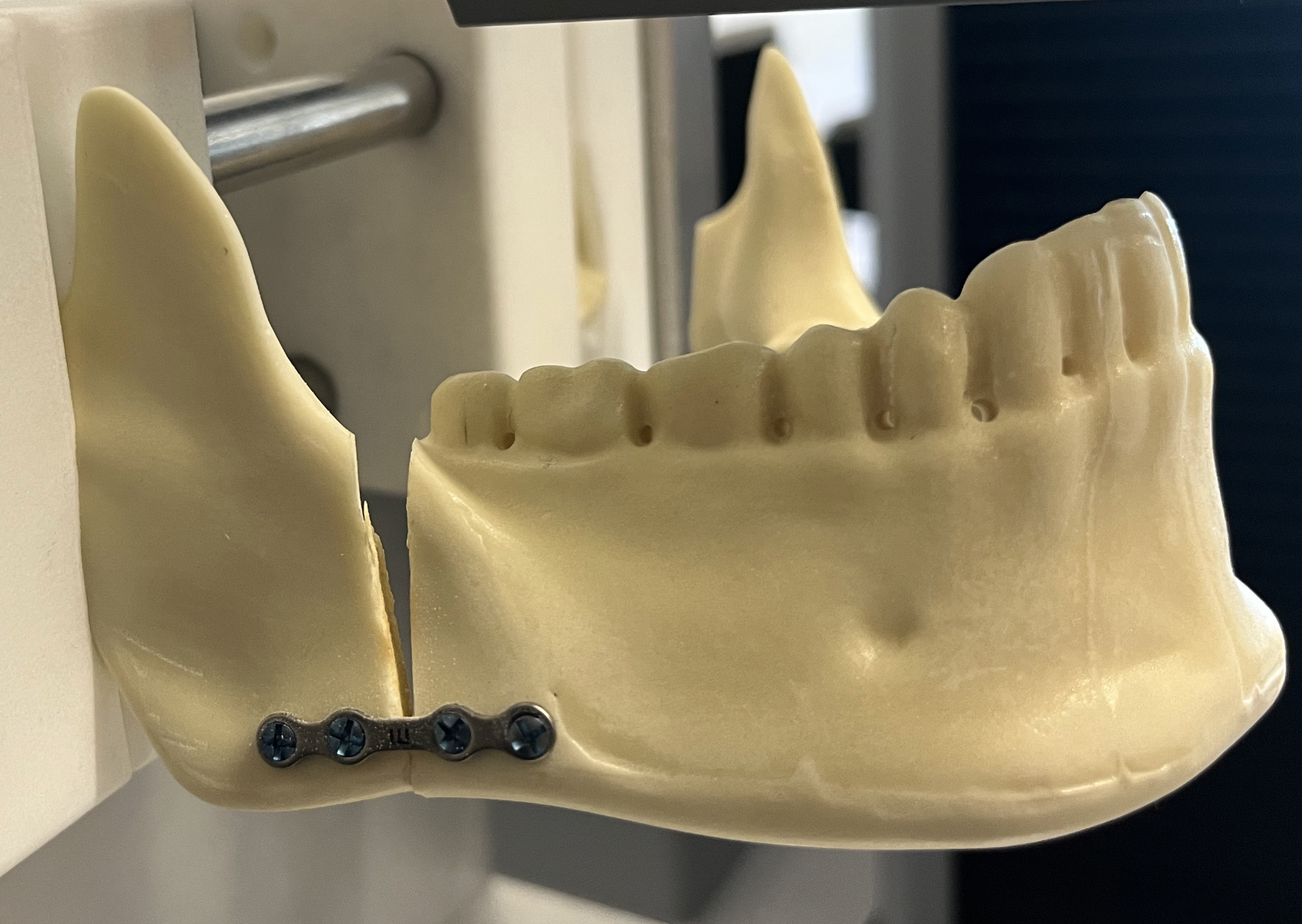** |
| **c1**  **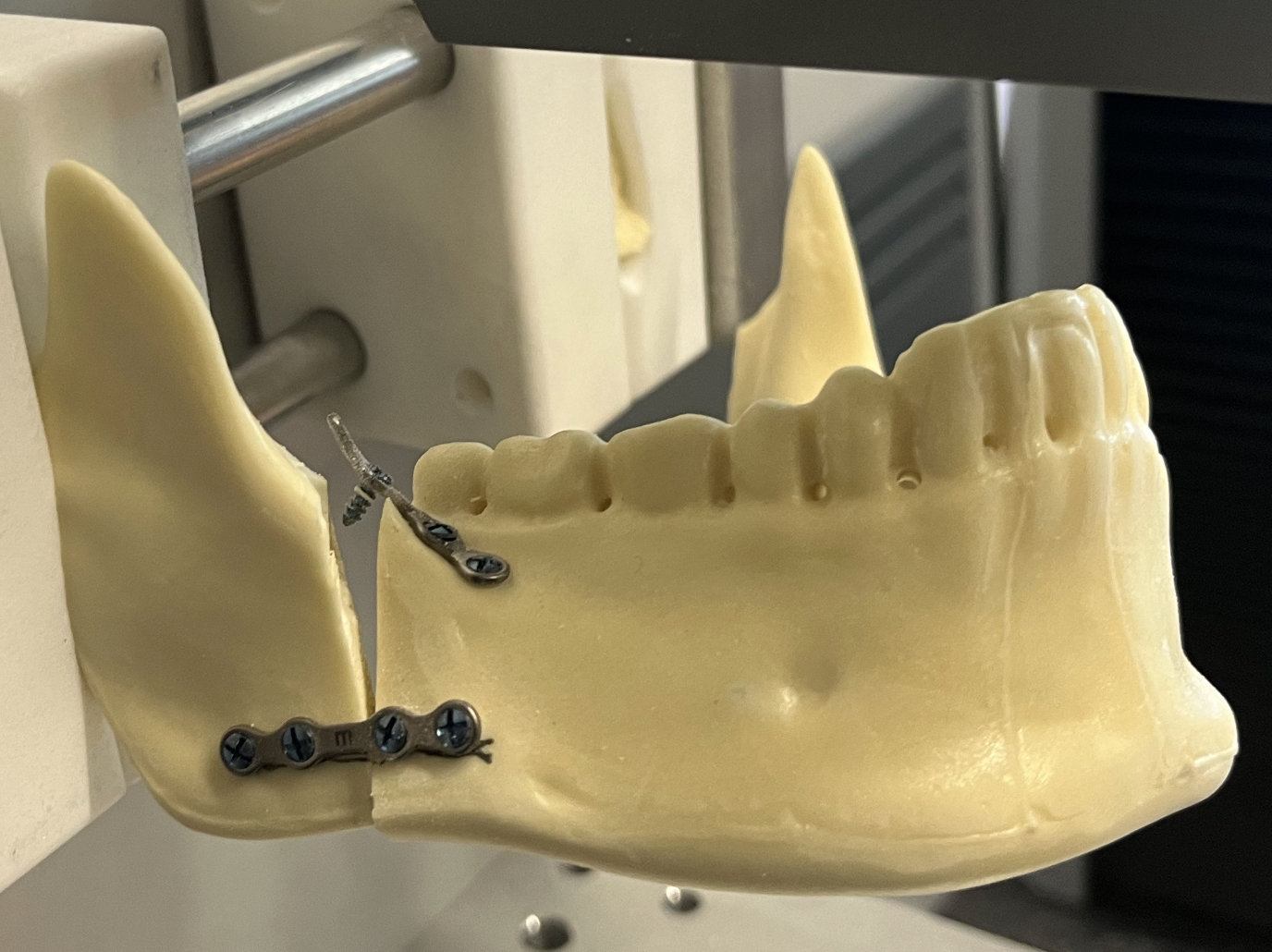** | **c2**  **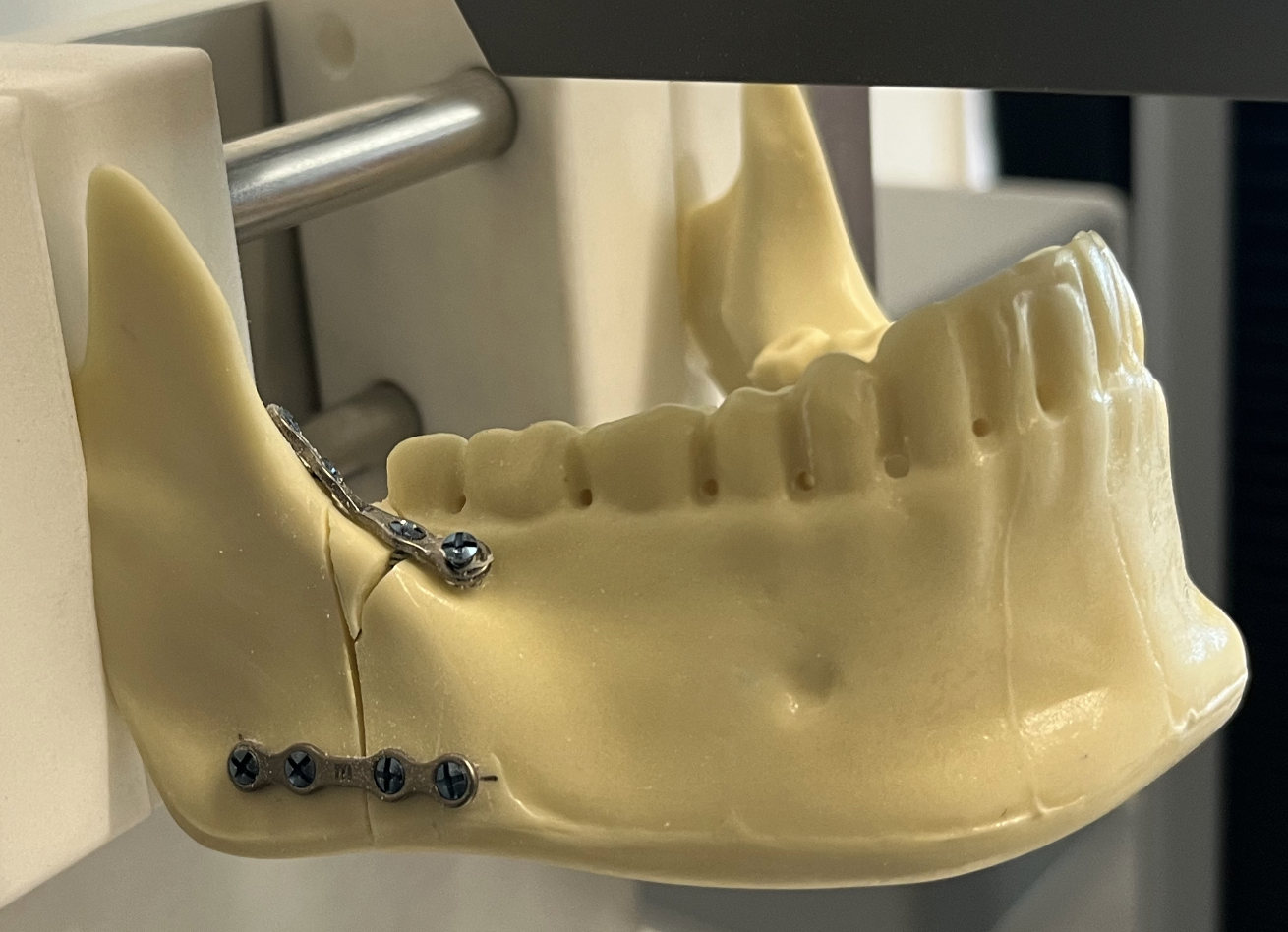** | **c3**  **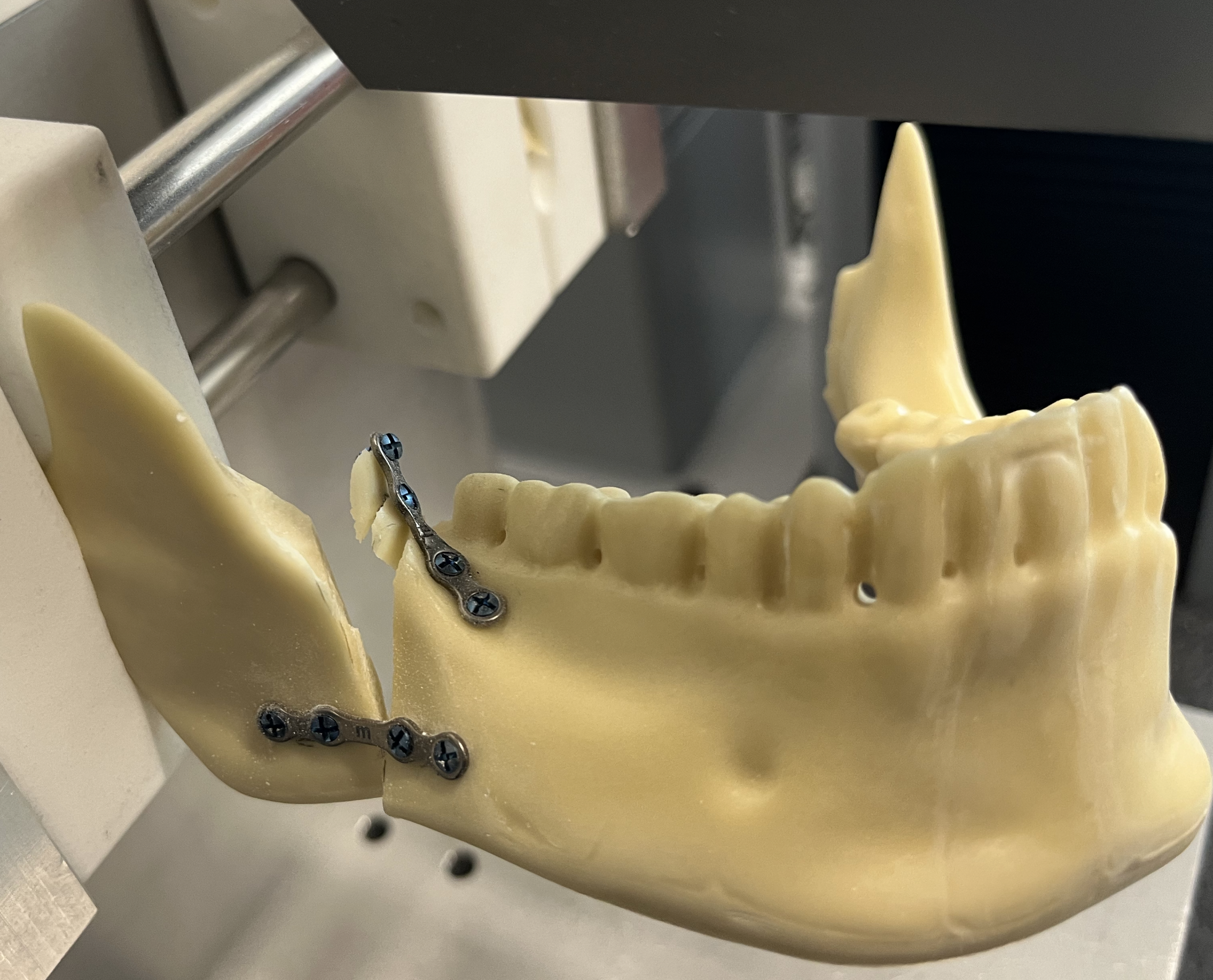** |

**Appendix 1 Figure A3.** The break pattern of mandible replicas with an angle fracture at the peak maximum force.

(**a**) Superior miniplate configuration: all the mandibles broke on the left fixated side where the mandible was fixated by the 3D printed mandible holders inside the mechanical test bench. (a2) second accrued break in the angle fracture region into three fracture fragments, and one screw was loose. (a3) second accrued break on the angle fracture side into three fragments and no loose screws.

(**b**) Inferior miniplate configuration: all the mandibles broke on the left fixated side where the mandible was fixated by the 3D printed mandible holders inside the mechanical test bench.

(**c**) Two miniplate configuration: all the mandibles broke on the left fixated side where the mandible was fixated by the 3D printed mandible holders inside the mechanical test bench. (c1) two screws from the superior miniplate became loose. (c2) second breakage on the angle fracture site resulting into three total fragments with no loose screws. The fragments accrued between the second and third screws. (c3) second accrued breakage on the angle fracture side, creating four total fragments.
