# Supplementary material for: Biomechanical assessment of mandibular fracture fixation using finite element analysis validated by polymeric mandible mechanical testing
Source: Sci Rep. 2024 May 23;14:11795. doi: 10.1038/s41598-024-62011-4 (PMC11116419; doi:10.1038/s41598-024-62011-4)
Supplement: Supplementary file 2 — Supplementary Information 2. [file 41598_2024_62011_MOESM2_ESM.docx]

**Appendix 2: Synbone material property testing**

**Appendix 2 Table A1.** Outcomes of the Synbone mechanical material properties chosen for the cortical and trabecular bone properties in the FEA.

| **Bone** | **Properties** | **Mean** | **SD** | **SE** | **SE-** | **SE+** |
| --- | --- | --- | --- | --- | --- | --- |
| **Cortical (GBHD)**  N = 5 | **Elastic modulus [MPa]** | 196.86 | 27.20 | 12.16 | 173.02 | 220.70 |
|  | **Tensile strength [MPa]** | 6.68 | 0.50 | 0.21 | 6.28 | 7.08 |
|  | **Yield strength [MPa]** | 48.12 | 3.61 | 1.47 | 45.23 | 51.00 |
|  | **Poisson’s ratio** | 0.01 | 0.11 | 0.05 | 0.01 | 0.19 |
|  | **Mass Density (g/cm3)** | 0.35 | - | - | - | - |
| **Trabecular (GB)**  N = 6 | **Elastic modulus [MPa]** | 60.97 | 9.36 | 3.82 | 53.48 | 68.46 |
|  | **Tensile strength [MPa]** | 3.31 | 0.19 | 0.08 | 3.15 | 3.46 |
|  | **Yield strength [MPa]** | 23.80 | 1.35 | 0.55 | 22.72 | 24.88 |
|  | **Poisson’s ratio** | 0.09 | 0.07 | 0.03 | 0.04 | 0.14 |
|  | **Mass Density (g/cm3)** | 0.19 | - | - | - | - |

*Abbreviations*: SD (standard deviation), SE (standard error), SE+ (standard error upper limit), SE- (standard error lower limit), and N (number of samples).

*Note*: the mass density was provided by the Synbone manufacturer.

| **a**  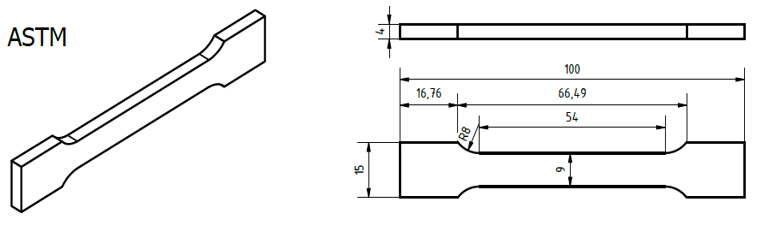 | **b**  **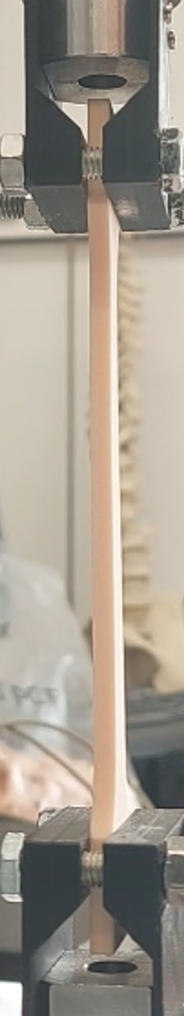** |
| --- | --- |
| **c**  **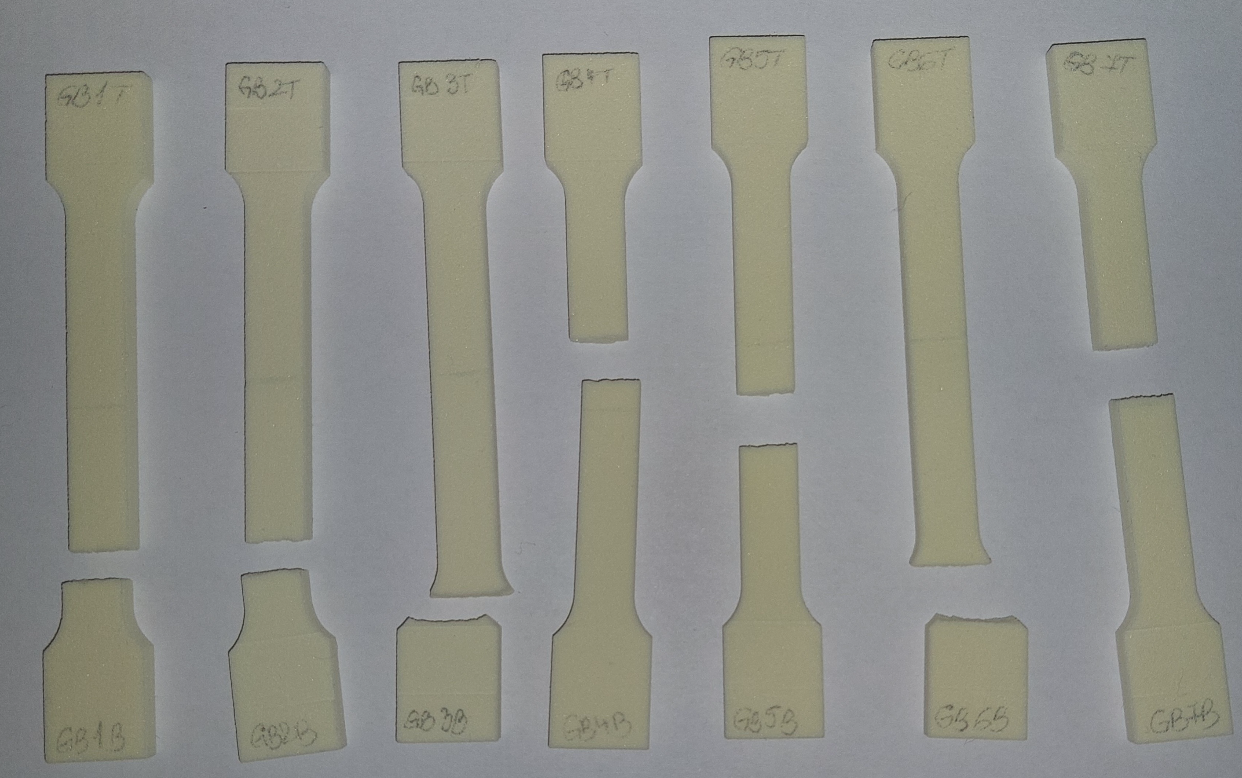** | **d**  **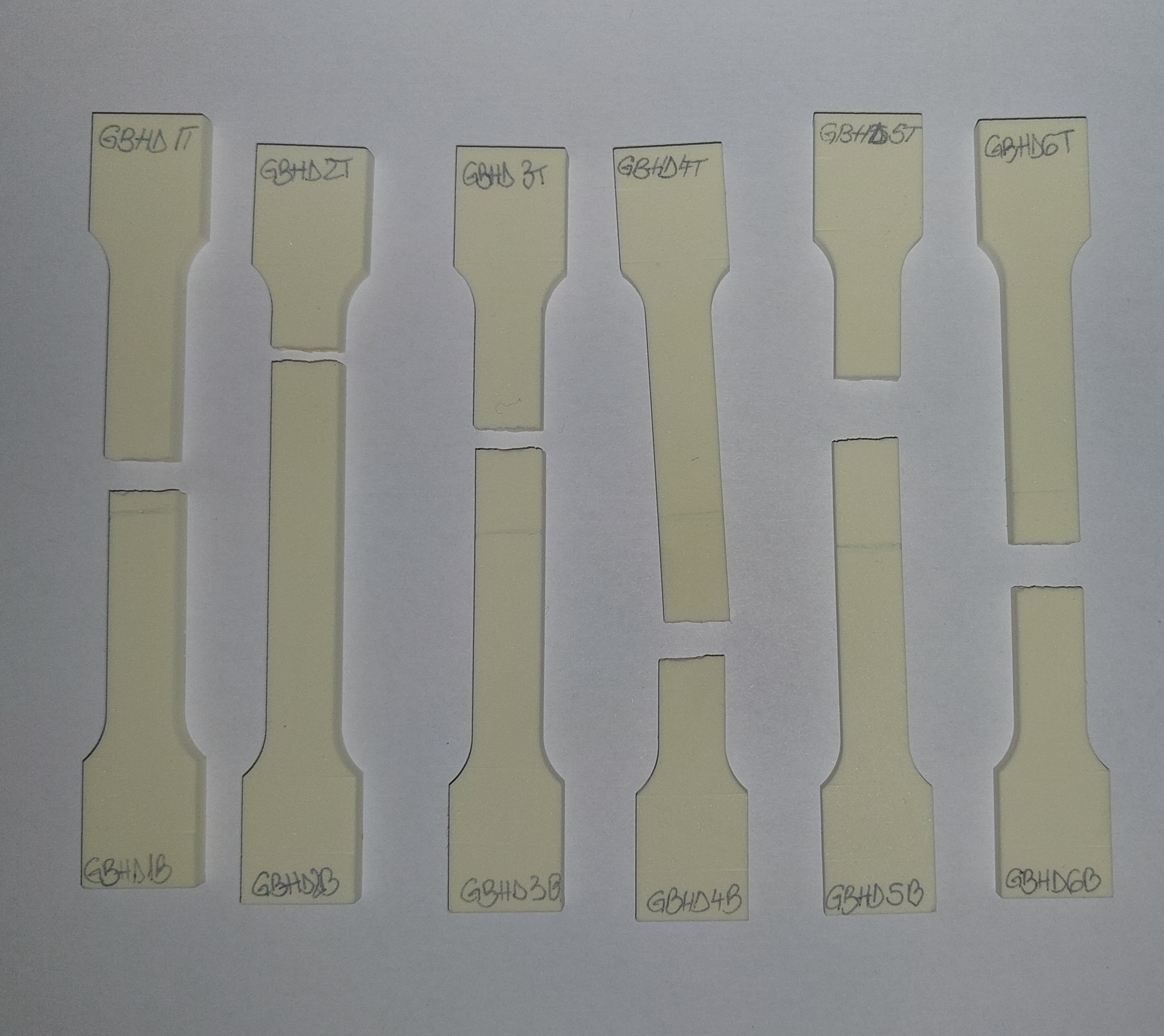** |

**Appendix 2 Figure A1.** (**a**) Test strips sizes according to the ASTM dimensions. (**b**) Test setup where the test strip was clamped inside the mechanical test bench. (**c-d**) Break pattern of the test strips: (**c**) GB, and (**d**) GBHD.

*Abbreviations*: ASTM (American Society for Testing and Materials), GB (Generic Block), and GBHD (Generic Block High Density).
