# Supplementary material for: Biomechanical assessment of mandibular fracture fixation using finite element analysis validated by polymeric mandible mechanical testing
Source: Sci Rep. 2024 May 23;14:11795. doi: 10.1038/s41598-024-62011-4 (PMC11116419; doi:10.1038/s41598-024-62011-4)
Supplement: Supplementary file 3 — Supplementary Figure S1. [file 41598_2024_62011_MOESM3_ESM.docx]

| **a1**  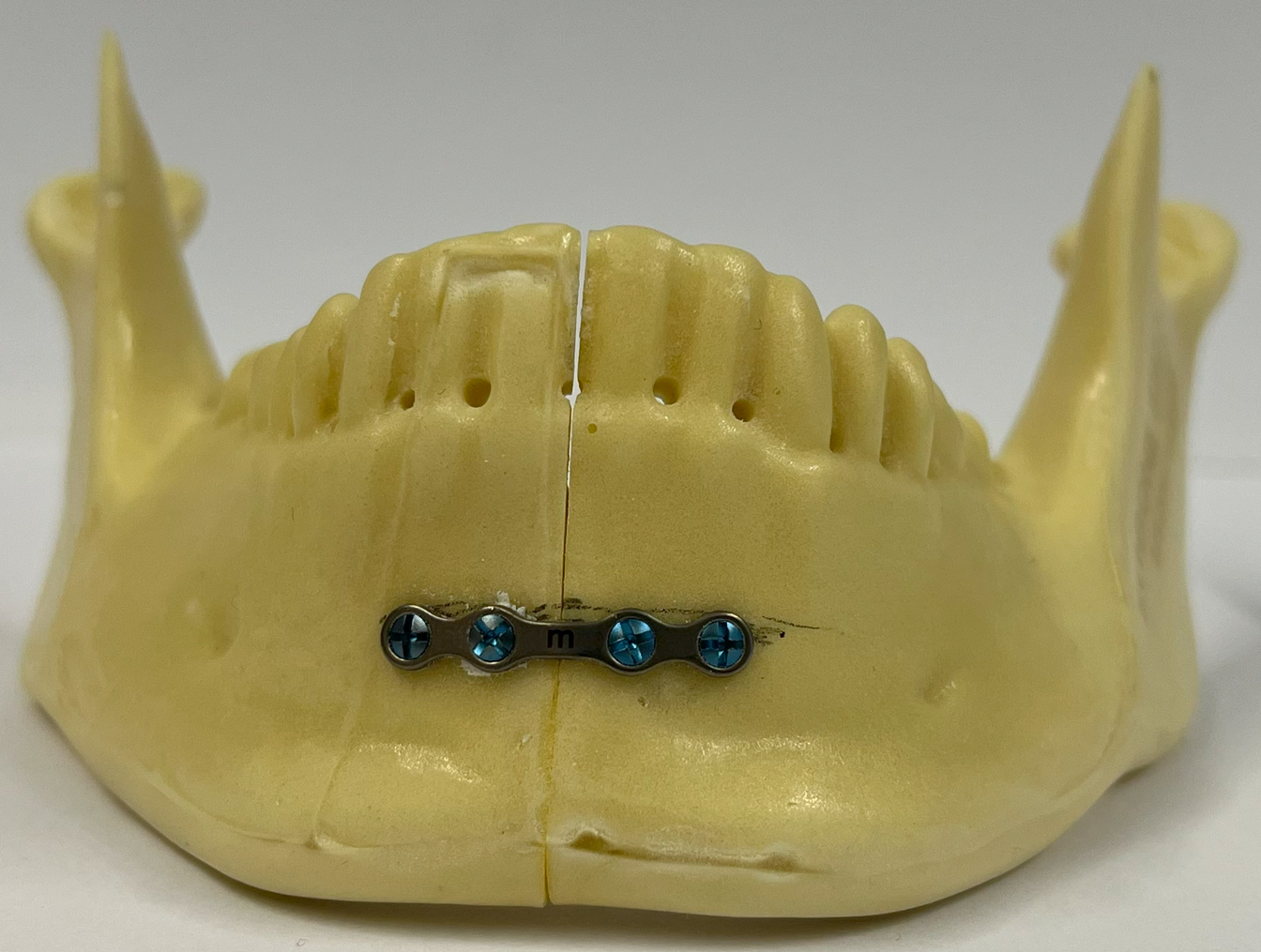 | **b1**  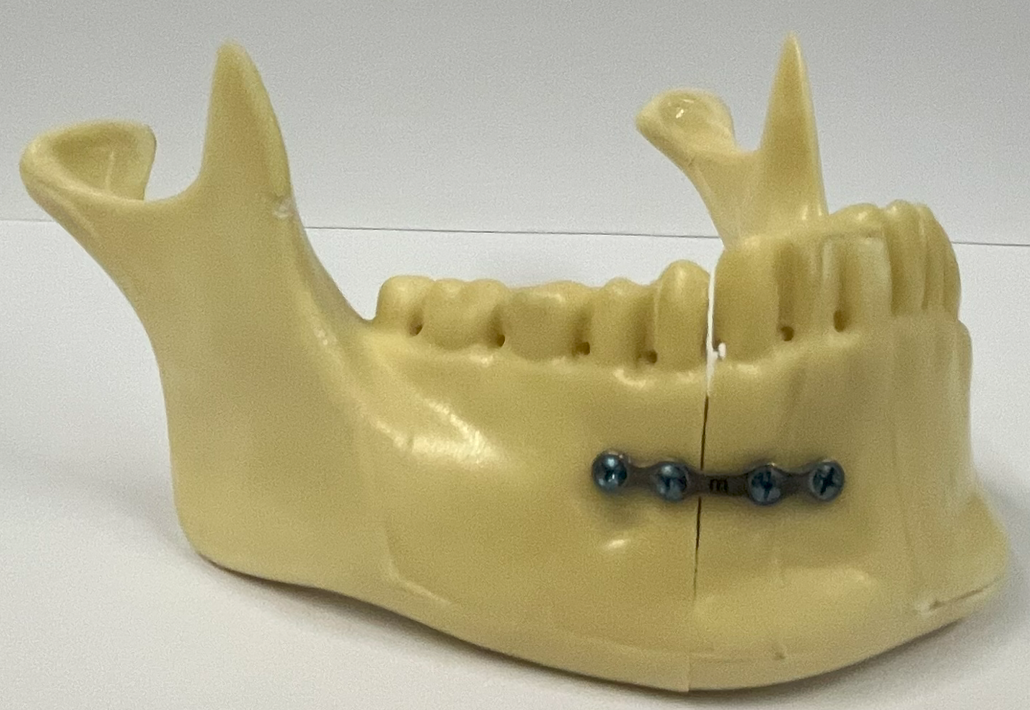 | **c1**  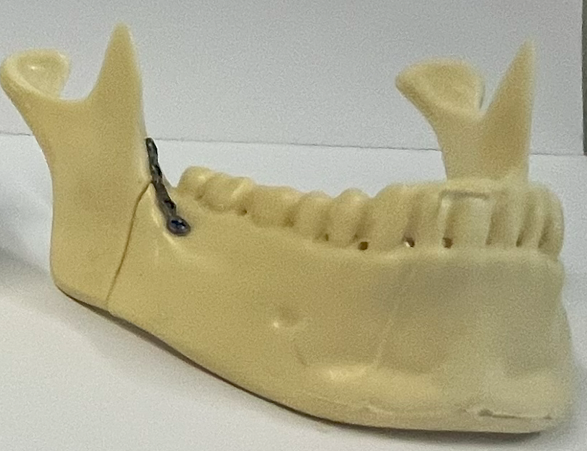 |
| --- | --- | --- |
| **a2**  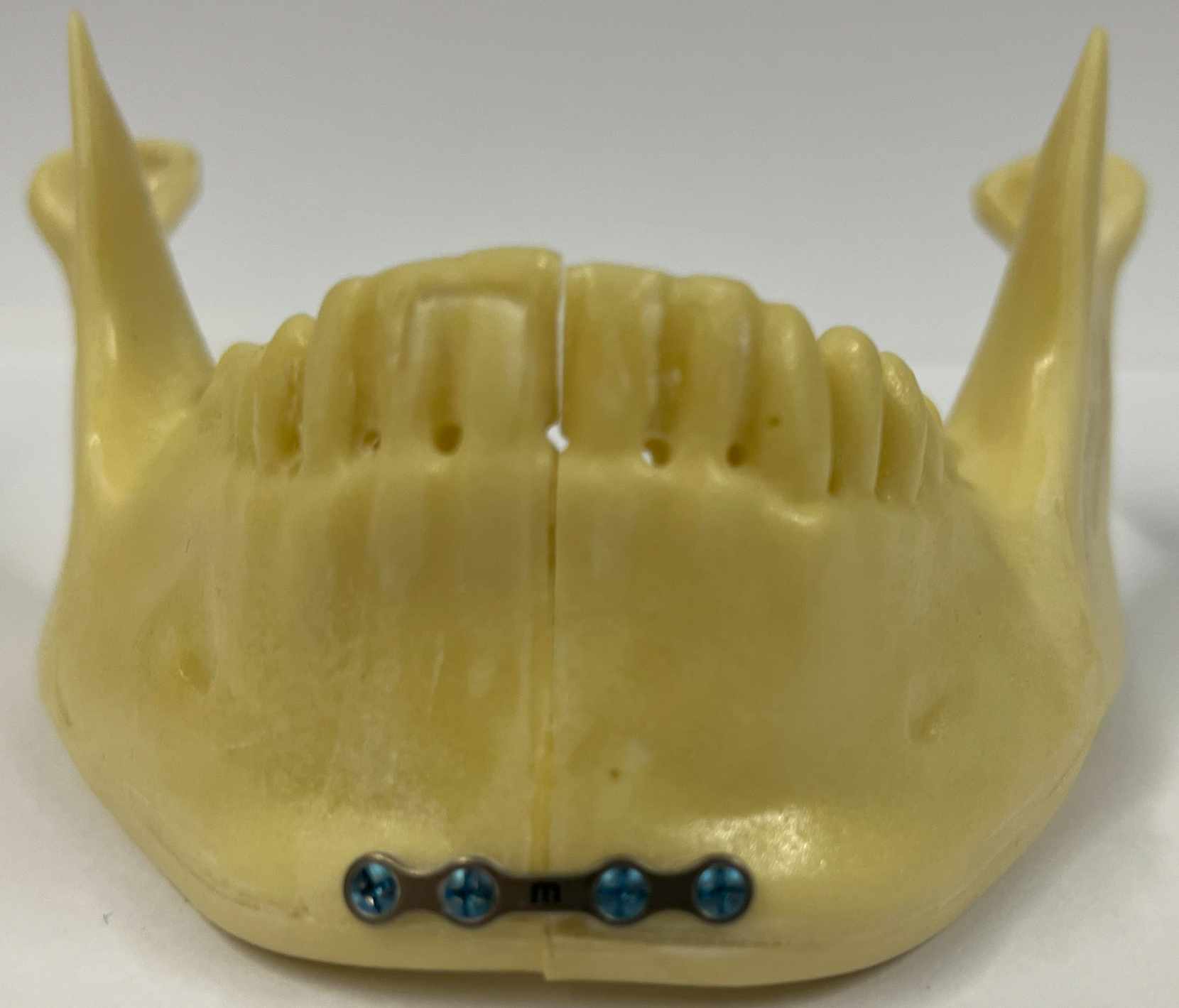 | **b2**  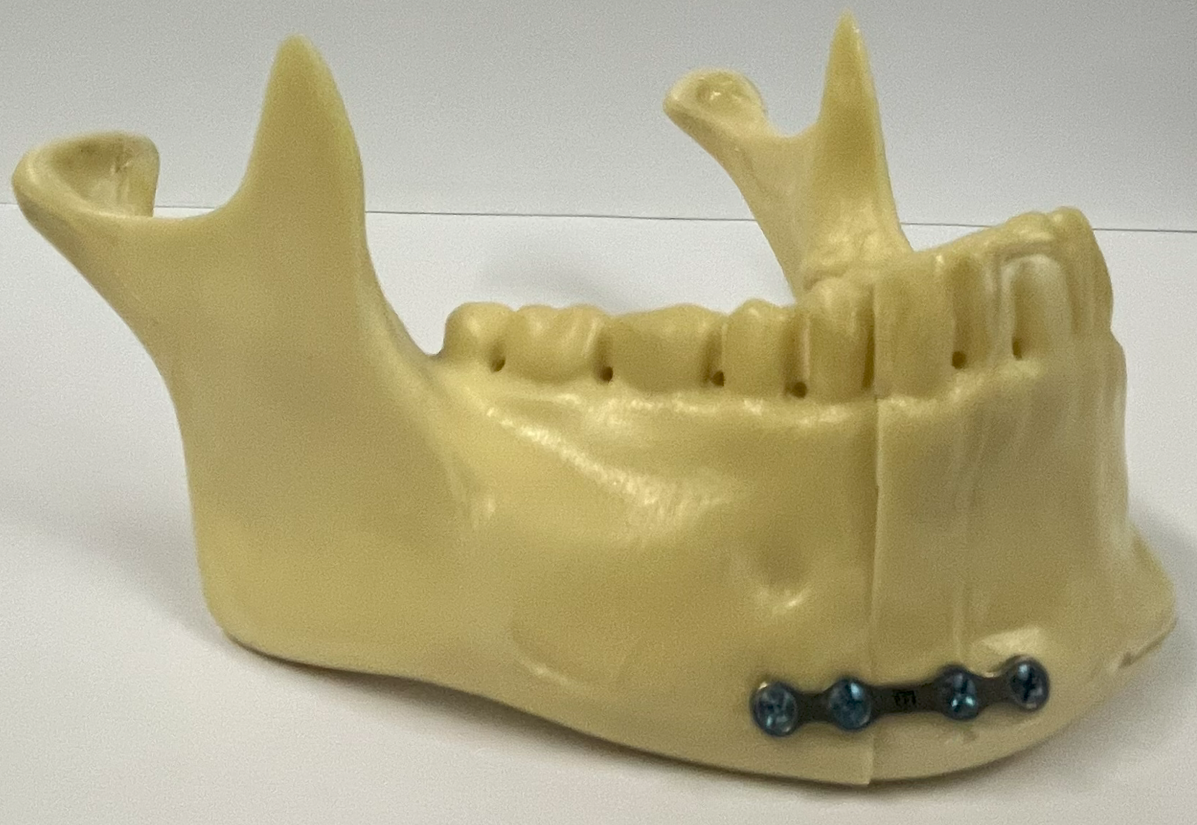 | **c2**  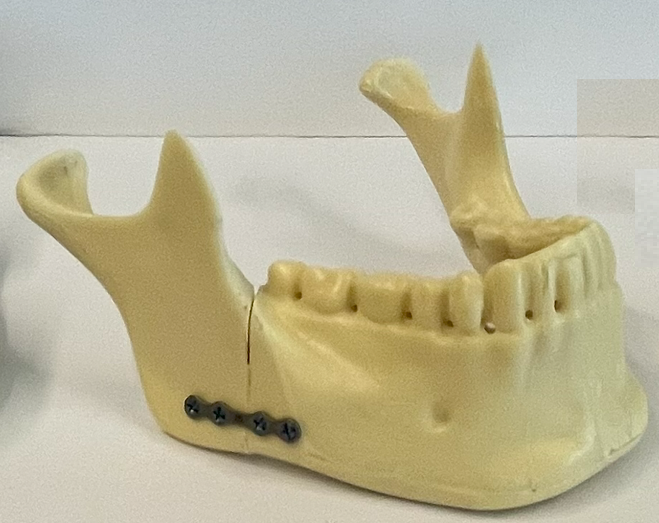 |
| **a3**  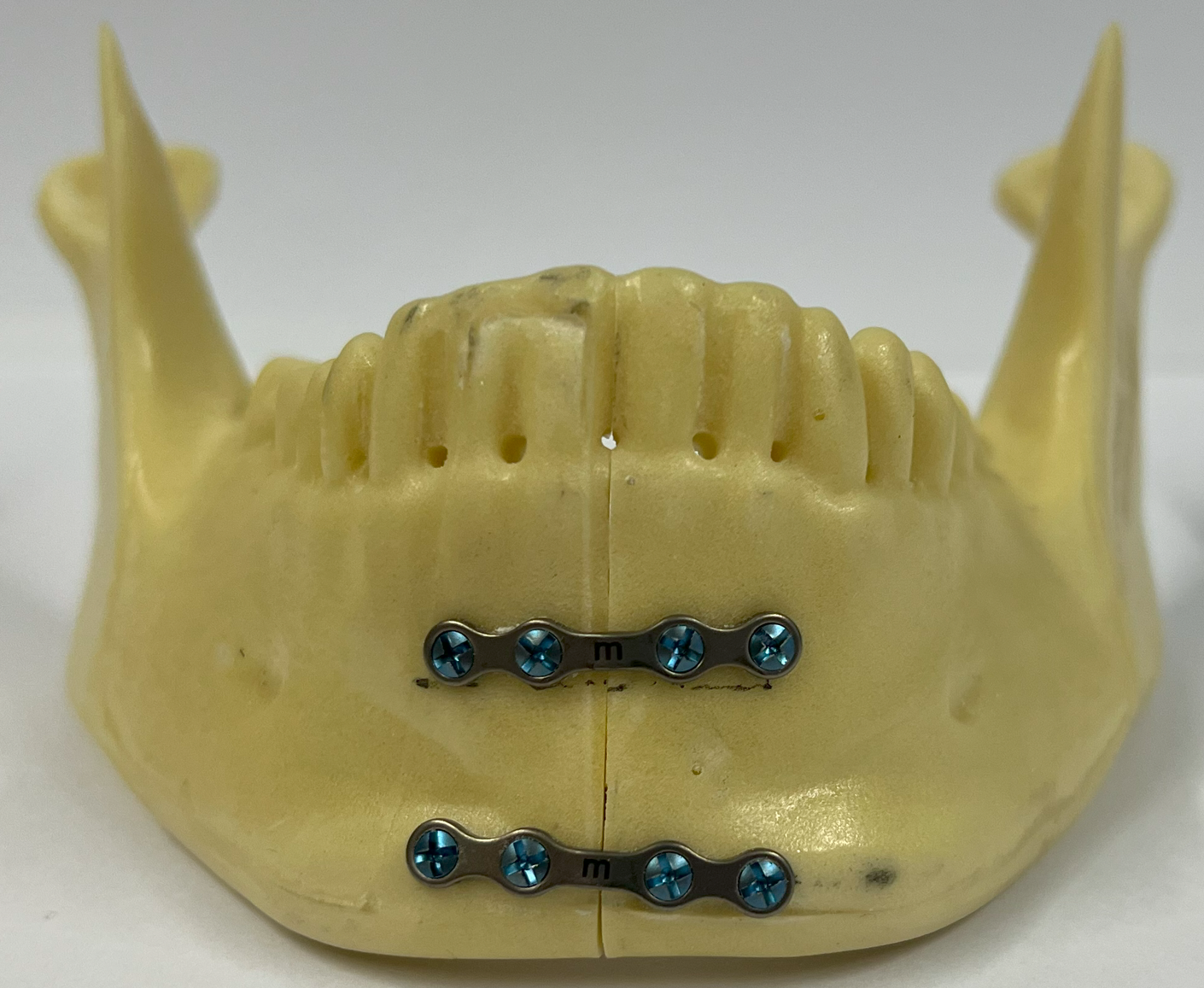 | **b3**  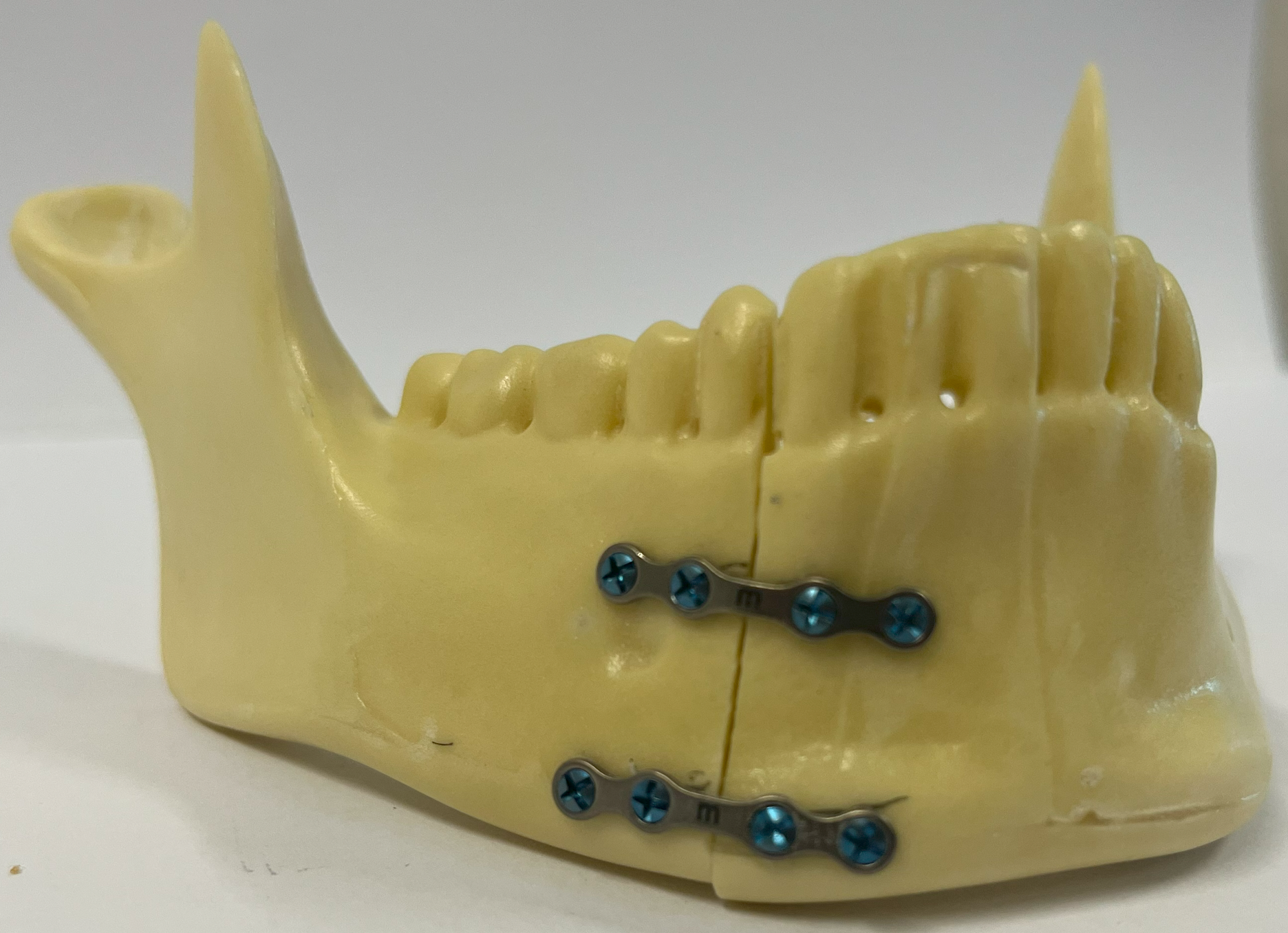 | **c3**  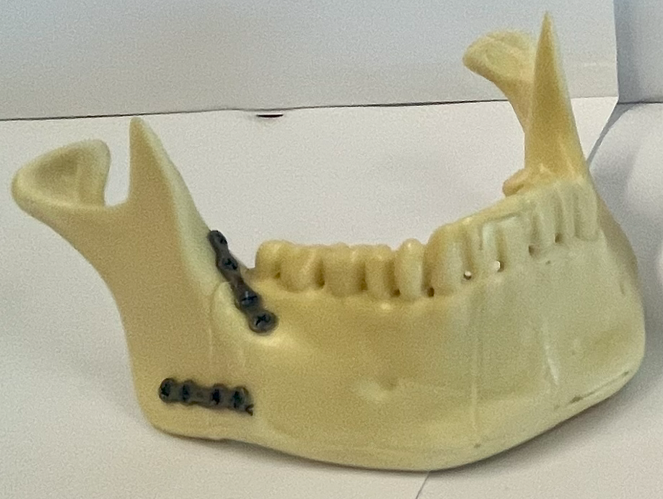 |

**Supplementary Figure S1.** PMMT miniplate positioning for mandibular (**a**) symphysis, (**b**) parasymphysis, and (**c**) angle fractures: with the miniplate positioned respectively at (**1**) the superior border, (**2**) inferior border, and (**3**) the two plate combination.
