# Supplementary material for: Biomechanical assessment of mandibular fracture fixation using finite element analysis validated by polymeric mandible mechanical testing
Source: Sci Rep. 2024 May 23;14:11795. doi: 10.1038/s41598-024-62011-4 (PMC11116419; doi:10.1038/s41598-024-62011-4)
Supplement: Supplementary file 4 — Supplementary Figure S2. [file 41598_2024_62011_MOESM4_ESM.docx]

**Supplementary Figure S2.** Mesh convergence: X-axis the number of elements and Y-axis peak Von-Mises stress in MPa. Note: The applied mesh had a minimum element size 1.68 mm and a maximum element size of 5 mm.
