# Supplementary material for: Biomechanical assessment of mandibular fracture fixation using finite element analysis validated by polymeric mandible mechanical testing
Source: Sci Rep. 2024 May 23;14:11795. doi: 10.1038/s41598-024-62011-4 (PMC11116419; doi:10.1038/s41598-024-62011-4)
Supplement: Supplementary file 5 — Supplementary Figure S3. [file 41598_2024_62011_MOESM5_ESM.docx]

| **a1**  **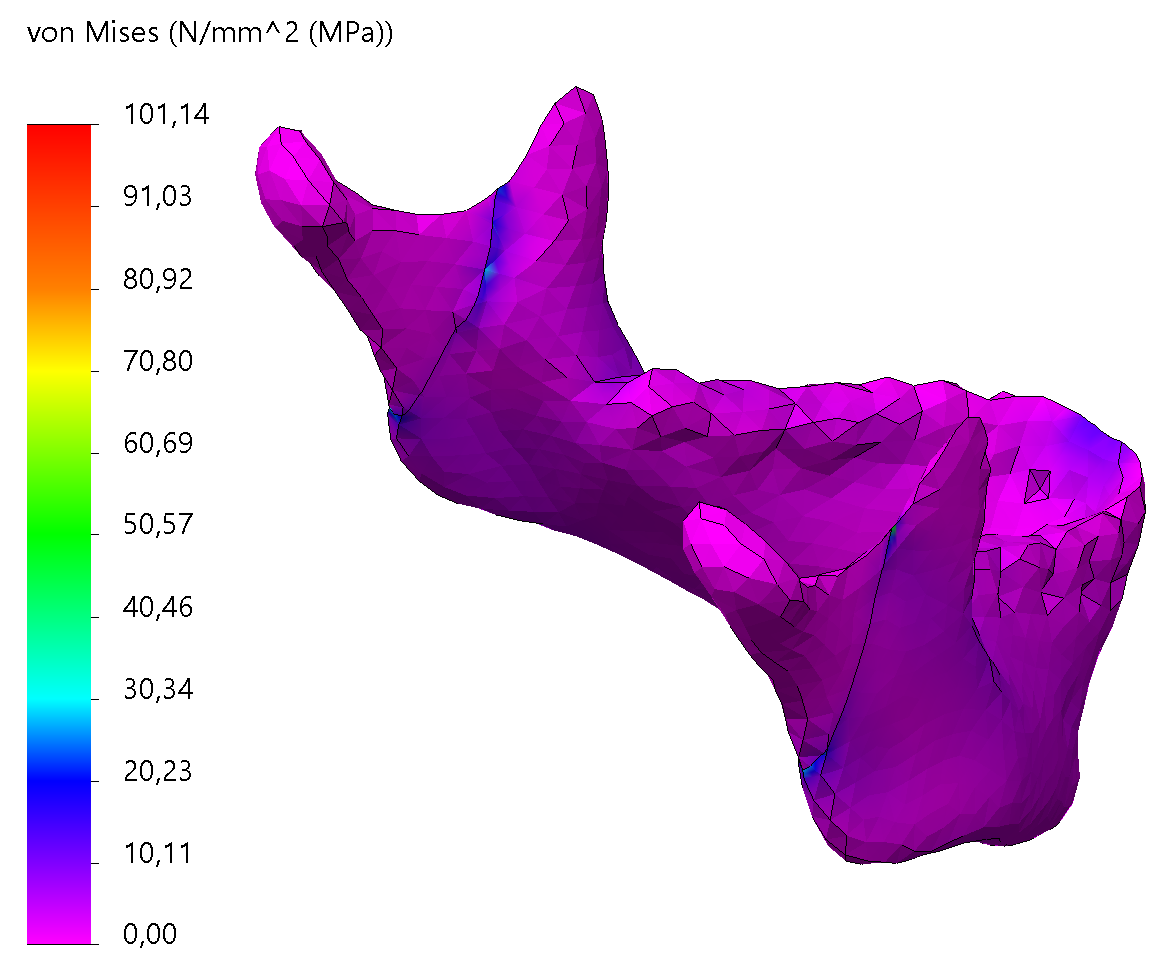** | **a2**  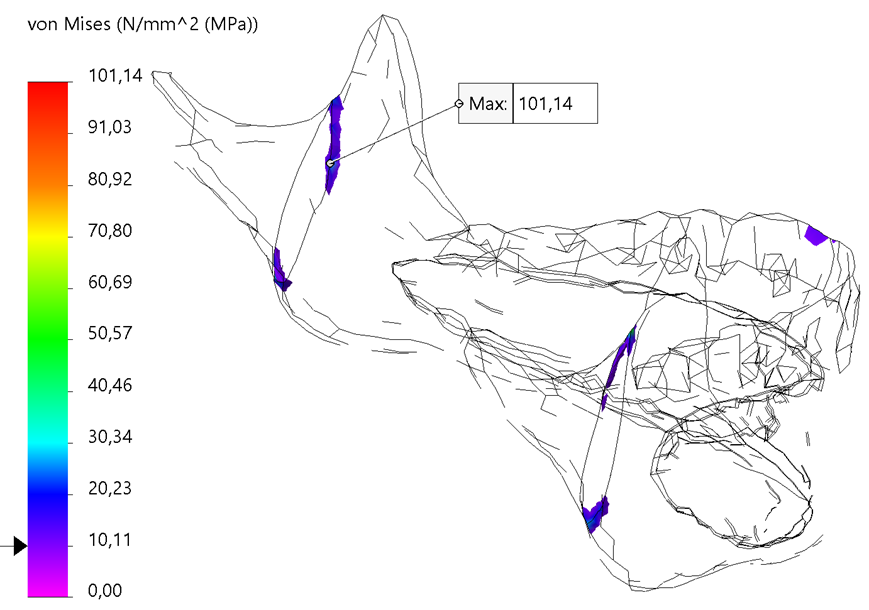 |
| --- | --- |
| **b1**  **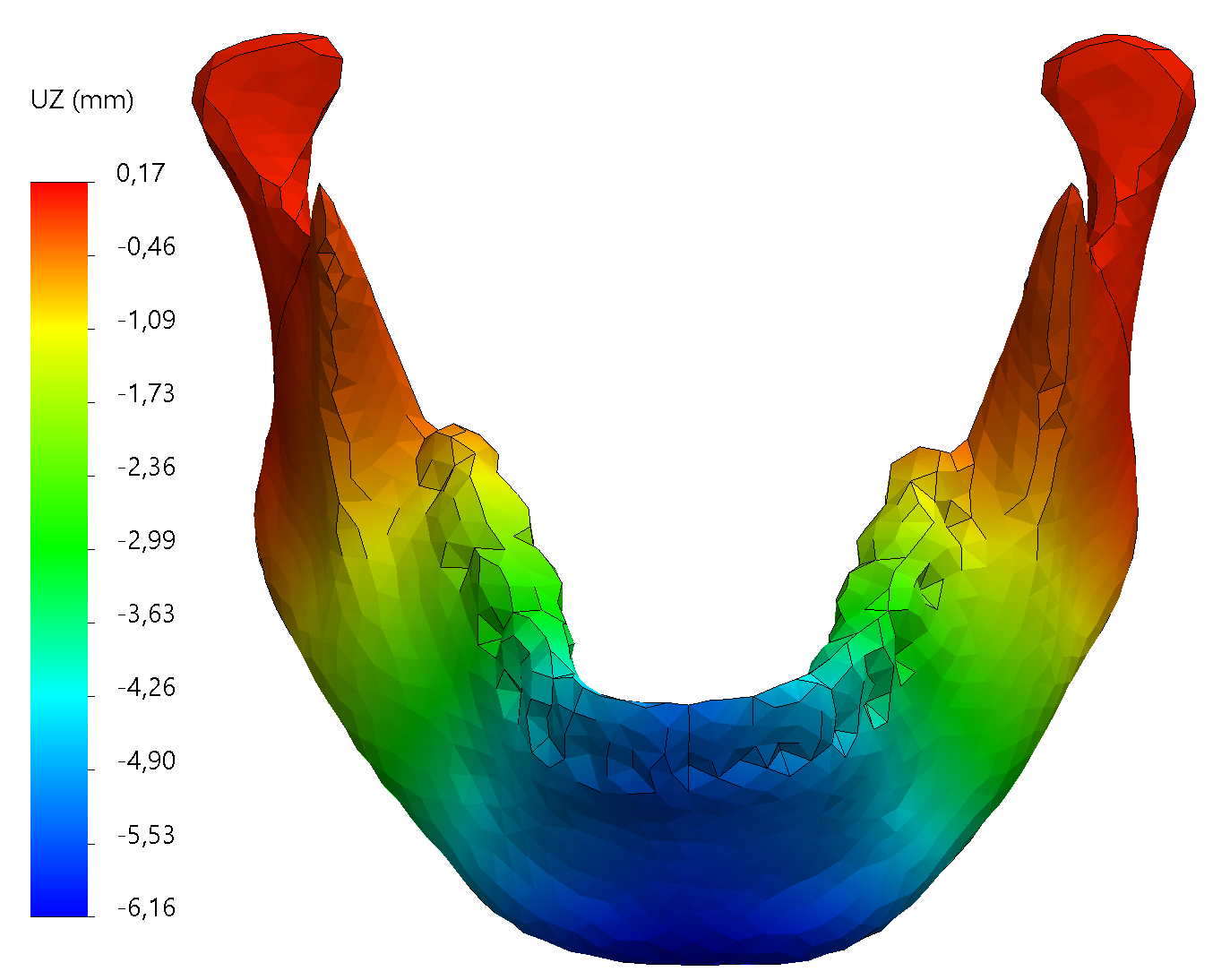** | **b2**  **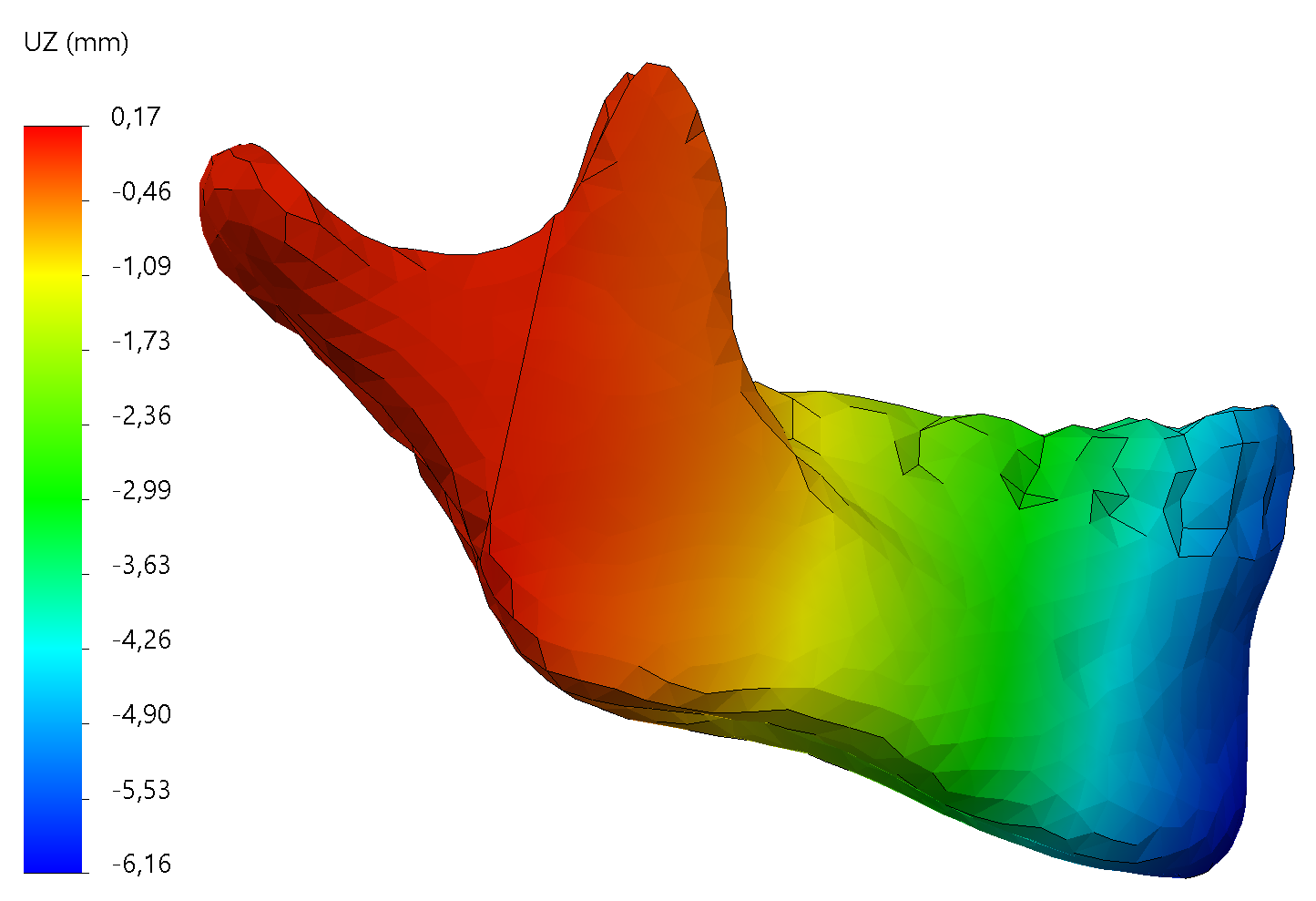** |

**Supplementary Figure S3.** FEA outcomes of a non-fractured mandible on applying a 200 N load. (**a**) Von-Mises stress in MPa: (**a1**) illustrating the stress pattern in the non-fractured mandible; (**a2**) focusing on the maximum stress region using the section clipping option in Solidworks (stress contour ≥ 10 MPa), illustrating the peak stress at the border of the fixation side with the mandible holders. (**b**) Illustrating the displacement pattern of the non-fractured mandible.
